# Genetics instability of wtAAV2 genome and AAV promoter activities in the Baculovirus/Sf9 cells system

**DOI:** 10.1371/journal.pone.0199866

**Published:** 2018-07-05

**Authors:** Adrien Savy, Minna U. Kaikkonen, Adrien Léger, Yohann Dickx, Lionel Galibert, Otto-Wilhelm Merten

**Affiliations:** 1 Généthon, Evry, France; 2 Université d'Evry Val d'Essonne, Evry, France; 3 Synpromics Ltd., Edinburgh, United Kingdom; 4 A. I. Virtanen Institute, University of Eastern Finland, Kuopio, Finland; 5 Atlantic Gene Therapies, Nantes, France; 6 Inserm, U1089 Nantes, France; 7 EMBL-EBI, Cambridge, United Kingdom; 8 FinVector Vision Therapies, Kuopio, Finland; University of Kansas Medical Center, UNITED STATES

## Abstract

The human Adeno-Associated Virus serotype 2 (wtAAV2) is a common non-pathological virus and its recombinant form (rAAV) is widely used as gene therapy vector. Although rAAVs are routinely produced in the Baculovirus/Sf9 cell system, wtAAV2 has never been studied in this context. We tried to produce wtAAV2 in the baculovirus/Sf9 cell system hypothesizing that the wtAAV2 may be considered as a normal recombinant AAV transgene. Through our attempts to produce wtAAV2 in Baculovirus/Sf9, we found that wtAAV2 p5 promoter, which controls the expression of large Rep proteins in mammalian cells, was active in this system. p5 promoter activity in the baculovirus/Sf9 cell system leads to the expression of Rep78 that finally excises wtAAV2 genome from the baculovirus genome during the earliest phases of baculovirus stock production. Via p5 promoter expression kinetics and strand specific RNA-Seq analysis of wtAAV2, rAAV and Rep2/Cap2 cassettes in the baculovirus context we could demonstrate that wtAAV2 native promoters, p5, p19 and p40 are all active in the context of the baculovirus system and lead to the expression of different proteins and peptides. In addition, this study has proven that the baculovirus brings at least some of the helper functions needed in the AAV replication/life cycle.

## Introduction

Wild type adeno-associated virus (wtAAV) has first been described as a contaminating virus in an adenovirus preparation [1] and was later developed as gene therapy vector because of its favorable features such as non-pathogenicity or its capacity to transduce dividing and non-dividing cells/tissues [2]

WtAAV2 is characterized by a 4,679 bases single-stranded DNA genome including Inverted Terminal Repeats (ITRs) [3] at both ends. ITRs are fundamental to the AAV biology and take part, amongst other functions, in the initiation of DNA replication [4]. The AAV genome encodes for 2 well described protein families as well as AAP [5] and the hypothetical X protein [6, 7]. The *cap* gene codes for the 3 structural elements VP1, VP2 and VP3, which are overlapping sequences. Their expression is driven by the unique p40 promoter. Alternative splicing with two different acceptor sites results in two transcripts. The larger one is translated into the virion protein 1 (VP1, 87 kDa). The shorter mRNA encodes the two other structural elements, VP2 (72 kDa) which originates from a non-canonical start codon (ACG) and VP3 (62 kDa) which is translated from a downstream conventional start codon (AUG). These three structural proteins differ from each other at their N-terminus sequences. VPs assembly leads to an icosahedral capsid composed of 60 VP proteins displaying a VP1:VP2:VP3 ratio about 1:1:10 [8]. This ratio reflects the relative efficiency of translation initiation of the three start codons and abundance of the two transcripts.

The four replicase proteins are encoded by the *rep* gene, under the control of two promoters located in position 5 (p5) and 19 (p19). Transcription from p5 and p19 resulting in two overlapping transcripts (4.2 and 3.6 kb), combined to internal splicing lead to four non-structural proteins, two large (Rep78 and Rep68) and two small (Rep52 and Rep40) named after their molecular weight. The large Rep proteins are involved in AAV DNA replication through fixation to the Rep-Binding Element (RBE) and the terminal resolution site (trs) sequences on ITR coupled with helicase and endonuclease activities. Large Rep proteins also mediate wtAAV2 genome integration into the host genome. They catalyze the resolution of the covalently closed AAV termini [9] which involves sequence specific DNA binding, strand specific nicking, and DNA unwinding mediated by an ATPase helicase domain [10]. Their functions are also required for the rescue of the wtAAV genome from its site of integration (excision of the wtAAV genome upon infection of the host cell by a helper virus (adenovirus, herpes virus [1]). Small Rep proteins are involved in the accumulation of single-stranded DNA from double stranded replicative intermediates and contribute to genome encapsidation into pre-formed capsids [11].

The baculovirus insect cell system was described as a very efficient and scalable production system for rAAV vectors [12,13]. Thus, we used the baculovirus/Sf9 expression system to produce various rAAVs, including one consisting of wtAAV2 sequence as recombinant genome (with the erroneous assumption of its inertness in the context of the baculovirus system). We discovered that wtAAV2 production was not possible in the baculovirus/Sf9 cell system due to undescribed and unanticipated activity of native AAV promoters leading to early wtAAV2 genome excision from the baculovirus backbone. The results obtained suggest that AAV promoters could be used for AAV production in context of the baculovirus system.

## Materials and method

**wtAAV2** sequence (NC_001401.2) has been obtained by gene synthesis from Genewiz company and cloned into pGRG25 plasmid [14].

### Recombinant Bacmid DNA

wtAAV2 sequence and other constructs have been transposed into modified Bac-to-Bac (Invitrogen) system (where *chitinase* and *cathepsin* genes were deleted) at Tn7 [15] site though pGRG25, pBF pPac 1 or pFastBac donor plasmids. Bacmid insertion was validated by PCR with M13 pUC primers (S1 Table).

### Generation of baculovirus stock

2 μg of bacmid DNA were transfected into 10^6^ Sf9 cells using Cellfectin II (Invitrogen) according to the manufacturer's instructions. Plates were incubated at 27°C. 5 days post-transfection, 10-fold serial dilutions of the transfection supernatant were used to perform lysis plaque assays. 10 days later, 5 isolated plaques were picked up and amplified in T25 cells flasks seeded with 5 million of Sf9 cells. After 5 days, baculovirus integrity and functionality was checked by qPCR and western blot, respectively. Two clones were then amplified in shake flasks (Corning). Baculovirus titrations were performed using the lysis plaque method as described in [16].

### Baculovirus DNA extraction from lysis plaque

Have been performed as described by McCarthy [17].

### Baculovirus sequencing

Sanger sequencing of the bacmid Tn7 site was performed by Beckman Coulter Genomics, using primers M13 pUC Fw and Rv (S1 Table).

### Fused Rep78-eGFP protein

pGRG25 p5-Rep78eGFP was built by Gibson Assembly (NEB). The first fragment coming from the 3'-end of Rep78 was amplified by PCR from the pGRG25-AAV2wt with primers Fragment_1_Fw and Fragment_1_Rv. Fragment 2 was obtained by amplification of the eGFP with Fragment_2_Fw and Fragment_2_Rv (S1 Table. Upper case bases represent overlapping sequences between fragments). pGRG25-AAV2wt was digested with BstBI and XcmI (NEB) restriction enzymes (WT AAV genome base 1,624 and 4,013). The remaining plasmid backbone was assembled with fragments 1 and 2, following the manufacturer’s protocol. The sequence was subsequently verified by Sanger sequencing.

### Rep78-eGFP ΔITR

The Rep78-eGFP ΔITR construct was built by PCR amplification of the pGRG25 p5-Rep78eGFP with the following primers Rep78eGFP_Fw and Rep78eGFP_Rv. The purified PCR product was cloned into pBF pPacI plasmid.

### p5-eGFP

The p5-eGFP construct with the ITR was built by Gibson Assembly (NEB). The p5 promoter sequence was generated by PCR on the pGRG25-AAV2wt with primers p5_Fw and p5_Rv. eGFP sequence was generated with primers eGFP_Fw and eGFP_Rv. The pGRG25-AAV2wt was digested with PpuMI and XcmI (NEB) restriction enzymes. Remaining plasmid backbone was assembled with p5 and eGFP fragments, following manufacturer’s protocol. The obtained DNA construct were asserted by sequencing.

### Tn7 Site PCR

Was performed using the Q5 hot Start Polymerase (NEB) with M13 pUC Fw and Rv primers following manufacturer’s protocol.

### Western blot

One million of transfected Sf9 cells were pelleted and the supernatant was removed. Pellets were resuspended in 100 μL of lysis buffer (Tris/phosphate 25 mM, pH 7.8, Glycerol 15%, DTT 1 mM, EDTA 1 mM, MgCl_2_ 8 mM, Triton 0.2%) with Protease Inhibitor Cocktail (04693116001—Roche). Samples were kept on ice for 30 minutes. 20 μL was mixed with LDS 4X (NP0007—Invitrogen) and 10X reducing agent (NP0004—Invitrogen). Samples were then heated 30 minutes at 94°C and ran on a polyacrylamide gel (NuPAGE 4–12% Bis-Tris Gel—Invitrogen). After migration, proteins were transferred onto a nitrocellulose membrane using the iBlot transfer system (Invitrogen, following manufacturer protocol). Membranes were saturated for 1h with blocking buffer (Odyssey). Rep detection was performed with Ig 259.5 (65158—Progen) whereas eGFP detection was performed using ab1218 (9F9.F9—Abcam) as primary antibody. Both were diluted 1/250. Antibody 680 LT (925–68020 Li-cor) diluted 1/20.000 was used as secondary Ab. WB against baculoviral p35 protein was performed with NB100-92383 rabbit antibody (Novus biologicals) diluted 1/5.000 as primary Ab and Ab 800 CW diluted 1/20.000 (925–32211 Li-cor) as secondary Ab. Images were acquired with Odyssey imager (Li-cor).

### Viral DNA extraction

Viral DNA extractions were done in triplicate from cell pellet or cellular culture. 5 μL of sample were added to 45 μL of DNase I buffer (Tris HCl 13 mM, CaCl_2_ 0.12 mM and MgCl_2_ 5 mM) with 10 U of DNase I (Invitrogen—90083), and digested for 30 minutes at 37°C. Capsid degradation was performed with proteinase K digestion (Roche) and purification of viral genomes were made using the MagNa Pure 96 DNA and Viral NA Small Volume kit and the MagNa Pure 96 instrument (Roche) following the manufacturer’s protocol. Elution volume was set to 50 μL.

### RNA extraction

Transfected cell medium was discarded; cells were washed once with PBS. After PBS removal, cells were lysed with Trizol. RNAs were purified with chloroform, then precipitated with isopropanol and washed with 70% ethanol. RNAs were then resuspended in RNAse free water. 5 μg of RNA were digested with 2 unit of DNase I (#18068–015—Invitrogen) for 3 hours at 37°C to remove residual DNA. DNase I was inactivated by addition of 1μL of EDTA 25 mM and heated 10 minutes at 65°C.

### Ribosomal RNA removal

Ribosomal RNAs were removed with Ribo-Zero Kit (Epicentre—Illumina) following manufacturer's instructions.

### RNA-Seq

RNA-Seq libraries were prepared from ribosome depleted RNAs as described [18]. Briefly, RNAs were fragmented and end-repaired using PNK-mediated dephosphorylation. After clean-up using the RNA Clean & Concentrator™-5 kit (Zymo Research), RNAs were polyadenylated and cDNA synthesis was performed using oligo dT-stretch and Illumina small RNA library adapter sequences. DNA-RNA hybrids were purified using the ChIP DNA Clean & Concentrator™ kit (Zymo Research), RNase H treated and circularized. cDNAs were amplified for 11–16 cycles and a product of 200–375 bp was extracted from 8% TBE Gel (Thermo Fisher Scientific). Libraries were quantified (Qubit dsDNA HS Assay Kit on a Qubit fluorometer, Thermofisher, Carlsbad, CA, USA) and pooled for 50 bp single-end sequencing with Illumina HiSeq2000 (GeneCore, EMBL Heidelberg, Germany).

### Bio-informatic pipeline and analysis

Libraries were sequenced for 50 cycles on an Illumina HiSeq 2000 according to manufacturer’s instructions. Fastq sequences were cleaned using FastqSweeper 0.1 (https://github.com/a-slide/FastqSweeper). FastqSweeper was run with default options and the following adapter sequences were trimmed: PolyA: AAAAAAAAAAAAAAAAAAAAAAAAAAAAAAAAAAAAAAAAAAAAAAAAA, Custom_adapter: AAAAAAAAAAAAAAAAAAAATCGTATGCCGTCTTCTGCTT and Truseq_indexed_adapter: GATCGGAAGAGCACACGTCTGAACTCCAGTCACNNNNNNATCTCGTATGCCGTCTTCTGCTT. After trimming, reads shorter than 25 nt were removed. The remaining reads were aligned against the draft genome assembly of Spodoptera frugiperda Sf21 [19], and only the non-matching reads were extracted in a new fastq file. FastqSweeper report is provided in S2 Table.

Alignment was performed using bowtie to the custom genomes (constructed based on known vector sequences, bacmid genome sequence, S4 .txt file). HOMER 4.3 (http://homer.ucsd.edu/homer/) was used to prepare bedGraphs and quantify the reads for all annotations in reads per kb per million reads (RPKM). RNA-Seq results were visualized with Integrative Genomic Software (Broad Institute).

### qPCR

Quantitative PCR (qPCR) was performed by TaqMan hydrolysis in LC480 (Roche). Baculovirus qPCR titration was performed on baculovirus DNA polymerase sequence with Bac_DNA_Pol_Fw, Bac_DNA_Pol_Rv primers and Bac_DNA_Pol_Probe.

For WT AAV titration wtAAV_Fw, wtAAV_Rv primers were used with the wtAAV_Probe.

For γ-SGC titration, SGC_Fw, SGC_Rv were used with SGC_Probe.

Primers are described in S1 Table.

### FACS

FACS analysis were performed with a BD FACSCalibur. Transfected cell medium was removed, cells were washed and resuspended in PBS before analysis. Data sets were analysed with FlowJo software.

## Results and discussion

### p5 promoter is active in baculovirus/Sf9 context and drives the expression of Rep78 leading to rAAV transgene excision

In mammalian cells, plasmid containing the wtAAV2 genome has to be co-transfected with a plasmid encoding adenovirus helper functions to enable efficient replication of the virus [20]. In the baculovirus expression system [15], recombinant baculoviruses are generated by transfection of bacmid DNA into Sf9 insect-cells. Baculovirus clones are then isolated by plaque lysis and amplified in culture flasks followed by analysis of transgene integrity using qPCR. On the other hand, recombinant AAV vectors are produced by co-infection of two baculoviruses bringing in the rAAV vector sequence and the Rep and Cap functions under the control of very-late baculovirus polyhedrin and p10 promoters, respectively. Following the amplification steps of the baculovirus encoding the wtAAV2 cassette, we were unable to detect any wtAAV2 genome by qPCR while the bacmid DNA used for transfection did contain the wtAAV2 genome. In parallel, recombinant baculoviruses harboring the gamma-sarcoglycanopathy gene (*γ*-SGC used as a reference gene [21]), had conserved their genetic integrity (i.e. equivalent copy number of *γ*-SGC amplicon and baculovirus DNA polymerase amplicon are obtained by qPCR).

**Fig 1:**
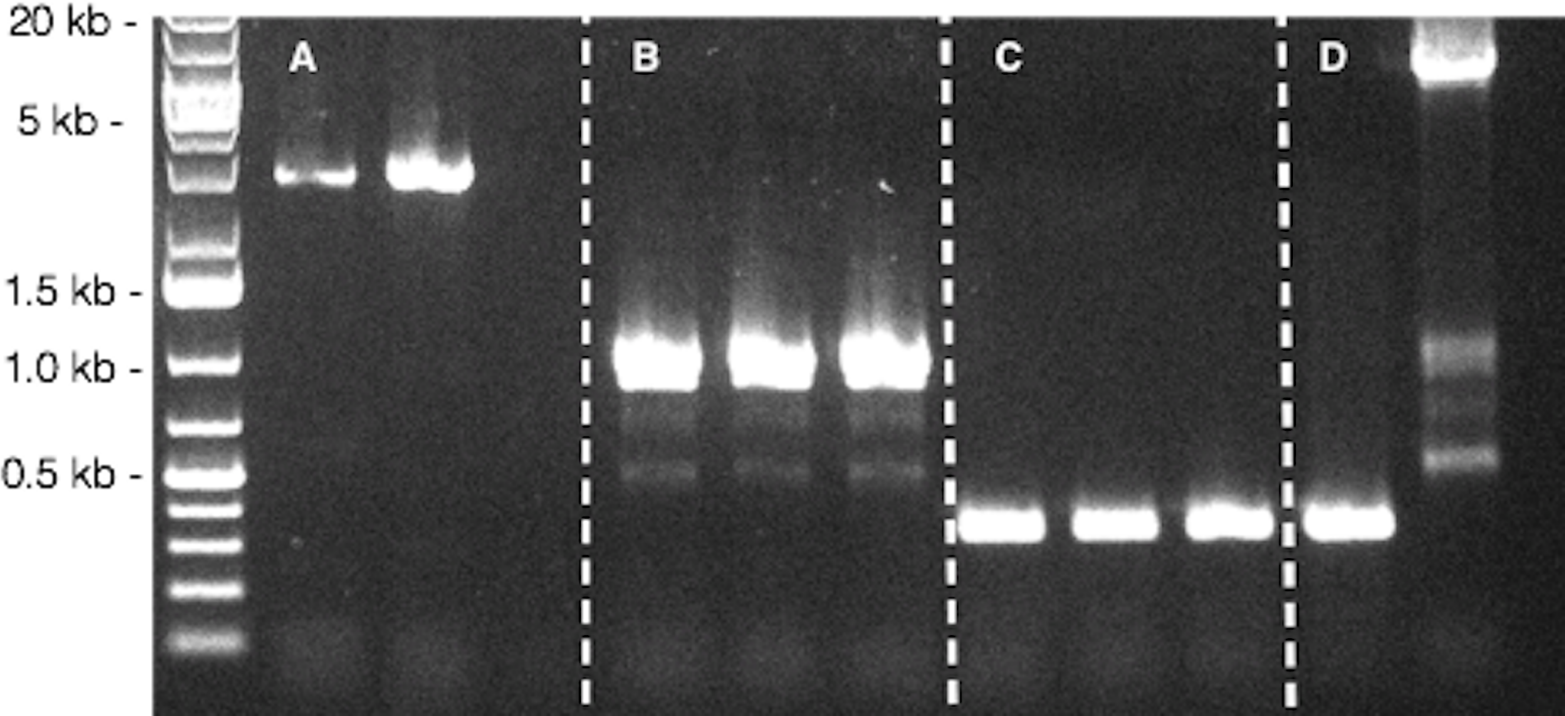
PCR at the baculovirus Tn7 site after clonal selection with lysis plaque assay. A. PCR bands from two baculoviruses carrying the *γ*-SGC transgene cassette (3.8 kb length). B. PCR from three clones of baculovirus carrying the wtAAV2 genome after lysis plaques. Bands are only one kb in length, meaning that wtAAV2 genome has been excised from the baculovirus backbone. C. PCR on three empty (Bac-to-Bac, Invitrogen) baculoviruses after lysis plaque displayed a 325 bp in length bands. D. PCR on empty bacmid DNA and on bacmid carrying the wtAAV2 genome. The gap between the PCR bands on row B and the second one on row D demonstrate the excision phenomenon.

To investigate how the loss of the wtAAV2 genome could happen, we extracted viral DNA five days after transfection and performed qPCR titration on ITR and the baculovirus backbone for both rAAV *γ*-SGC, wtAAV2. Same copy numbers of the recombinant rAAV/wtAAV2 genome cloned in the baculovirus and of the baculovirus backbone alone confirmed that recombinant cassettes were still present, with an integrity value close to one. Baculoviral DNA was then extracted from well isolated lysis plaques after ten days in order to perform a PCR on the insertional Tn7 site. After insertion at the Tn7 site, the PCR products should have been of 5.5 kb and 4.6 kb for wtAAV2 and rAAV *γ*-SGC sequences, respectively. PCR products were of 1 kb for the wtAAV2 cassette and of the expected size for the rAAV *γ*-SGC. These results indicated that the wtAAV2 genome was somehow excised from the bacmid backbone for all selected clones, but not the rAAV *γ*-SGC cassette used as control (Fig 1). The 1 kb product obtained was then sequenced. It corresponded mostly to recombinant bacmid genome, but instead of containing the full wtAAV2 genome, we only detected the first 25 bases of the 5’ ITR (data not show). No trace of the 3’ ITR was detected, suggesting its full excision.

Taking into account the endonuclease activity of Rep78 on ITRs, we hypothesized that it would be a good candidate to explain the excision of the wtAAV2 cassette from the baculovirus genome. To test this hypothesis, new bacmids were generated where eGFP was fused to Rep78, either with or without the ITRs (Rep78-eGFP ΔITR), under the control of wtAAV2 p5 promoter.

**Fig 2:**
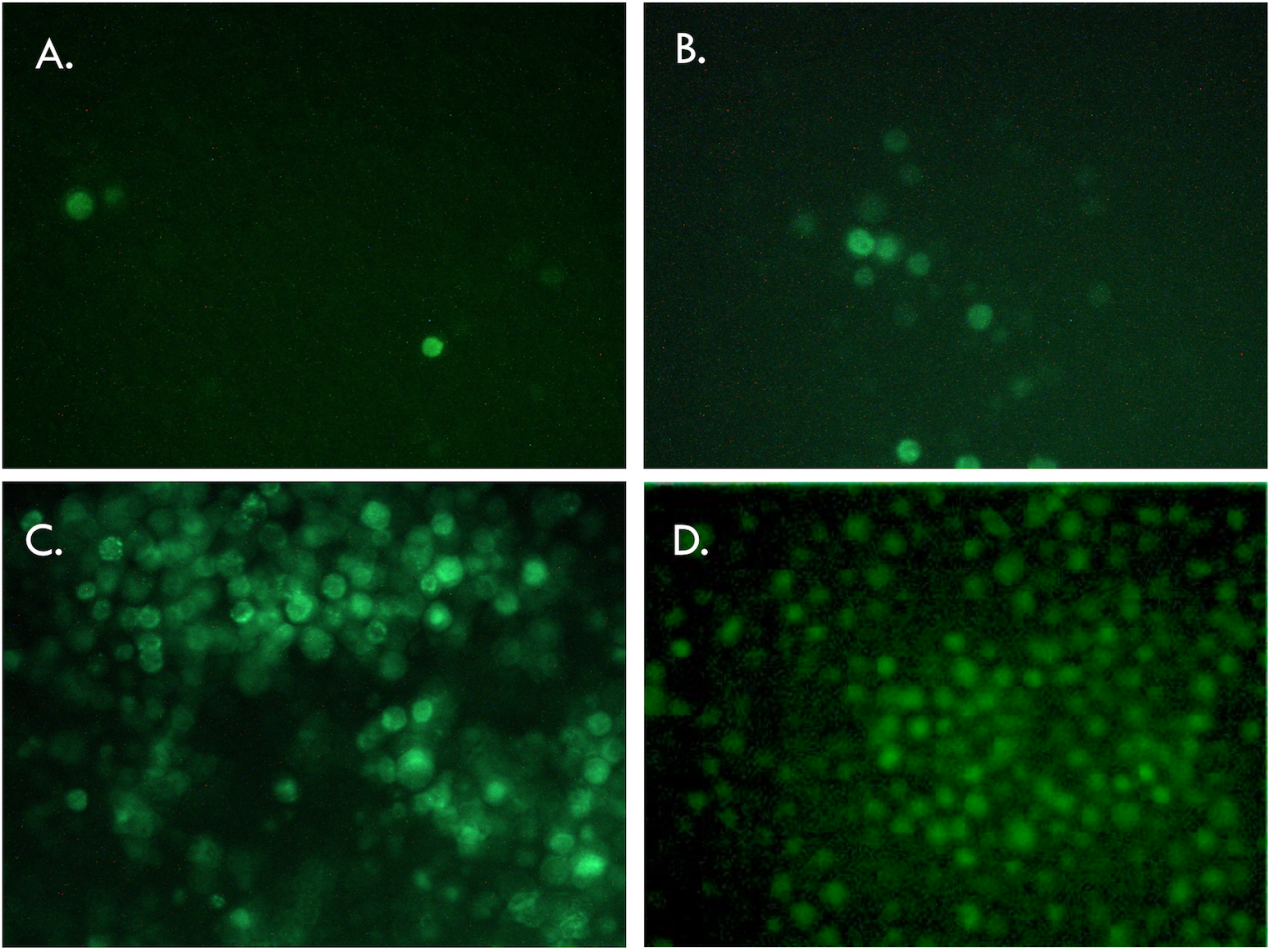
Fluorescence microscopic observation of Sf9 cells transfected with bacmid containing the Rep78eGFP cassette and expressing GFP. **A, B** and **C**, 30, 55 and 120 hours post transfection of bacmid containing the Rep78eGFP fusion protein under the native p5 promoter. GFP is visible after 30 hours post transfection, and the combination of baculovirus replication followed by cell infection led to near 100% of the cells being GFP positive 120 hours post transfection. **D.** 120 hours post transfection with the bacmid containing the construct Rep78eGFP delta ITR.

**Fig 3:**
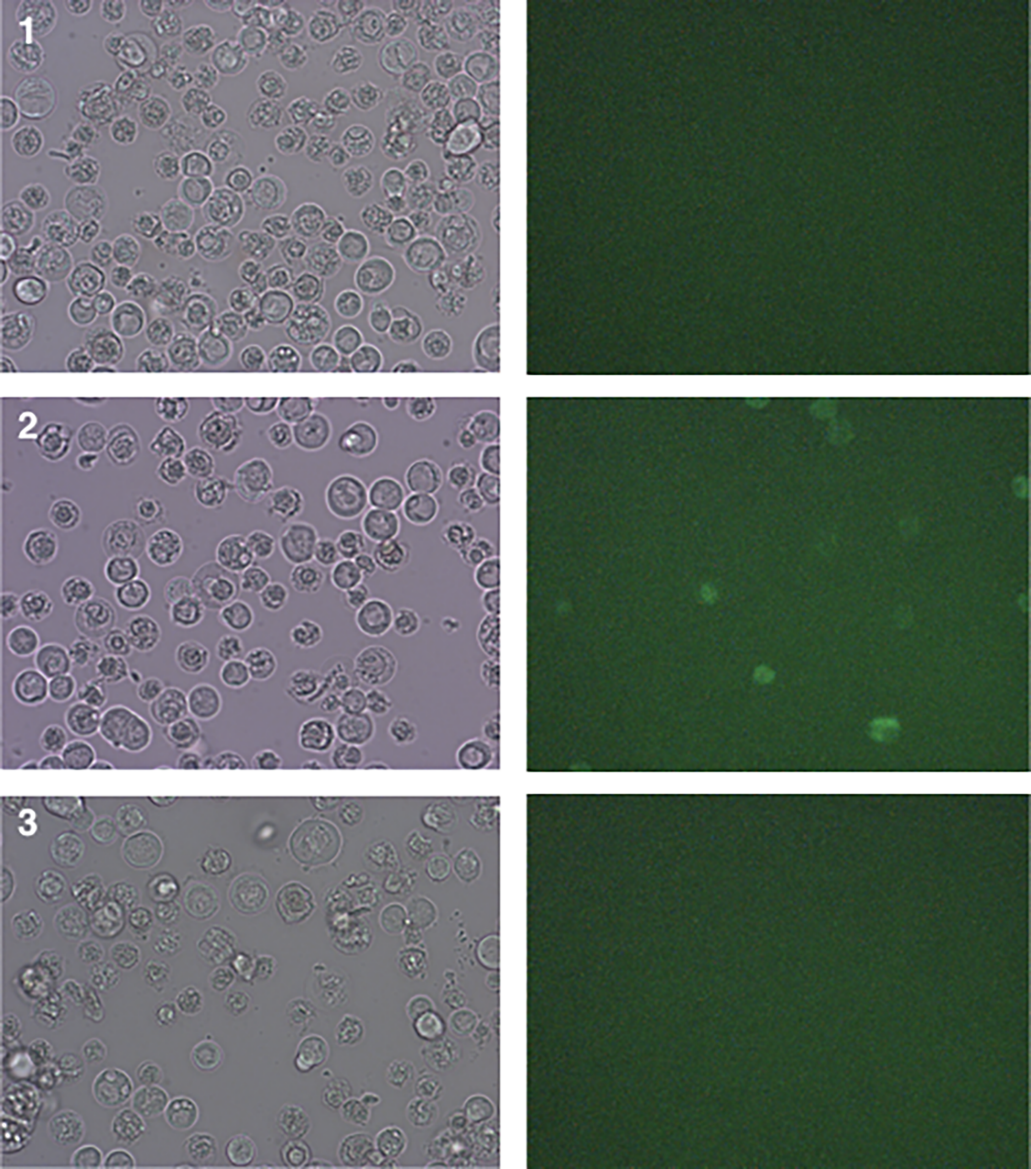
Microscopy observation of infected Sf9 cells 5 days post-infection. Three different baculovirus (carrying the Rep78-eGFP ITR construct) lysis plaque clones have been used to infect T75 cell flasks (panels 1–3). Despite obvious signs of baculoviruses infection, only in one flask eGFP expression could be observed, meaning that the ITR-Rep78eGFP-ITR construct has been excised from the baculovirus backbone before the reinfection in most of the cases.

Transfection of bacmid (Rep78-eGFP-ITR) allowed detection of eGFP expression in Sf9 cells (Fig 2). Following this first expansion of baculoviruses, clonal selection was performed using the lysis plaque technique and isolated clones were expanded in culture flasks for 5 days. Out of the five colonies tested for Rep78-eGFP with ITRs, four exhibited no eGFP expression, despite evident signs of baculoviruses infection. For the last clone few green cells were detected (Fig 3). All baculovirus encoding Rep78-eGFP in the absence of ITRs displayed eGFP positive cells (data not show). The loss of eGFP expression between the bacmid transfection step and infection after clonal selection only observed with the construct harboring Rep78-eGFP between the ITRs could be explained by the cassette excision from the baculovirus backbone through its own Rep78-eGFP nicking and helicase activities. This result indicates that p5 led to Rep78 expression, which means that the native AAV p5 promoter is active in the baculovirus/Sf9 system and that Rep78 expression leads to genome excision only when coupled with the presence of ITRs.

### p19 promoter is active in baculovirus

**Fig 4:**
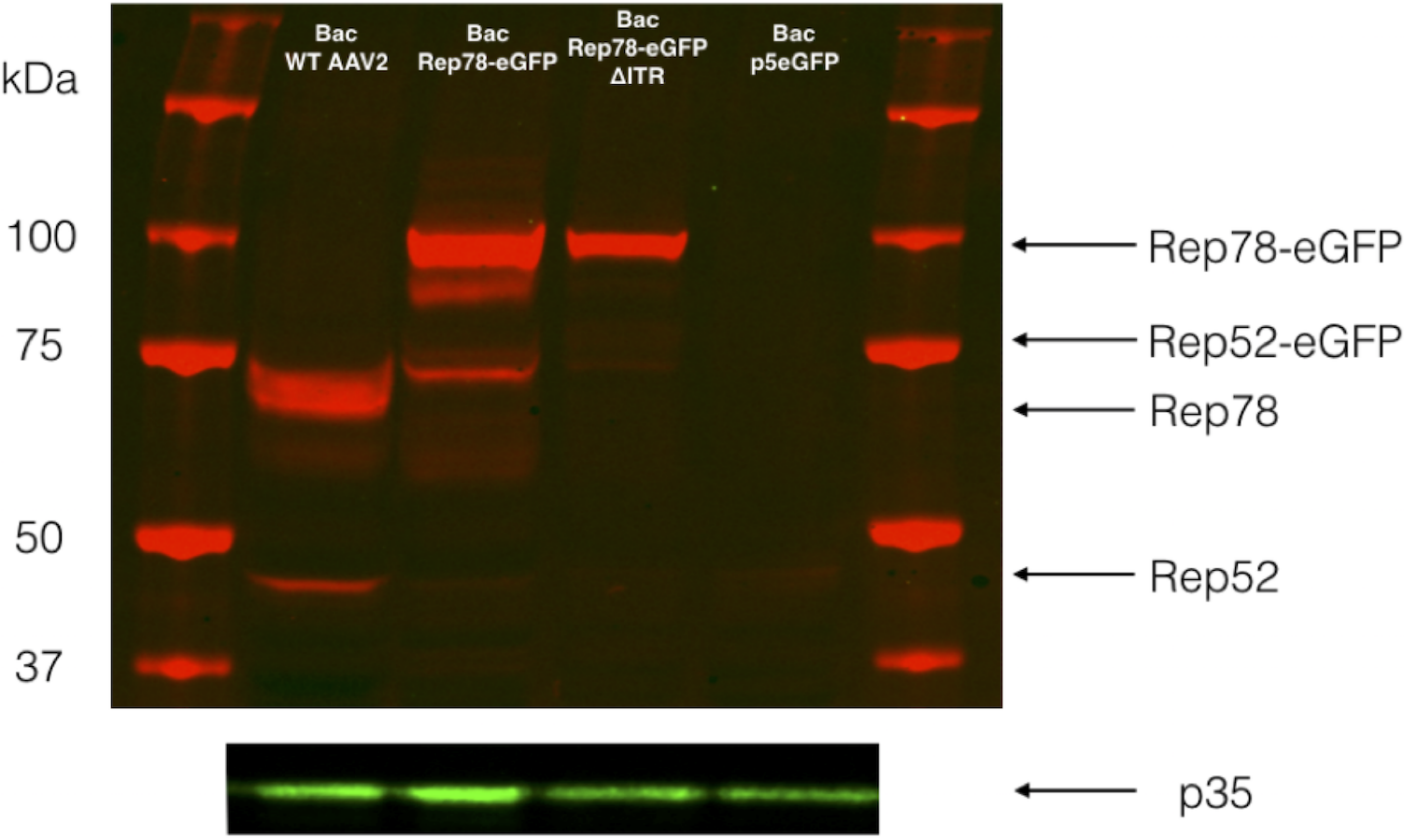
Western blot against Rep proteins, five days post transfection with different bacmid constructs (as indicated above the rows). Large Rep78 proteins are always detectable, excepted for the p5eGFP construction. Small Rep52 proteins are largely less detectable than large Rep proteins. The gap between Rep size can be explained by the eGFP fusion. p35 proteins represent the overall baculovirus proteins amount.

Based on the unanticipated activity of p5 and since p19 activity was previously reported to drive the expression of Rep52 in the baculovirus context [22], we wonder whether the fluorescence observed after transfection with the p5-Rep78-eGFP construct was the result of p5 activity alone or if Rep52-eGFP was also expressed from p19. To study this, two additional constructs were used. The first one consisted in p5-Rep78-eGFP ΔITR, the second only harbored p5-eGFP between the ITRs (p5-eGFP). Fig 4 shows a western blot against Rep proteins 5 days post-transfection where Rep78 and Rep52 could easily be detected in the wtAAV2 construct and Rep78-eGFP and Rep52-eGFP for both fused constructs. For all samples studied, Rep52 was detected to a lesser extent than the large Rep78. It should be noted that both spliced forms Rep68 and Rep40 were not detected in our western blot.

**Fig 5:**
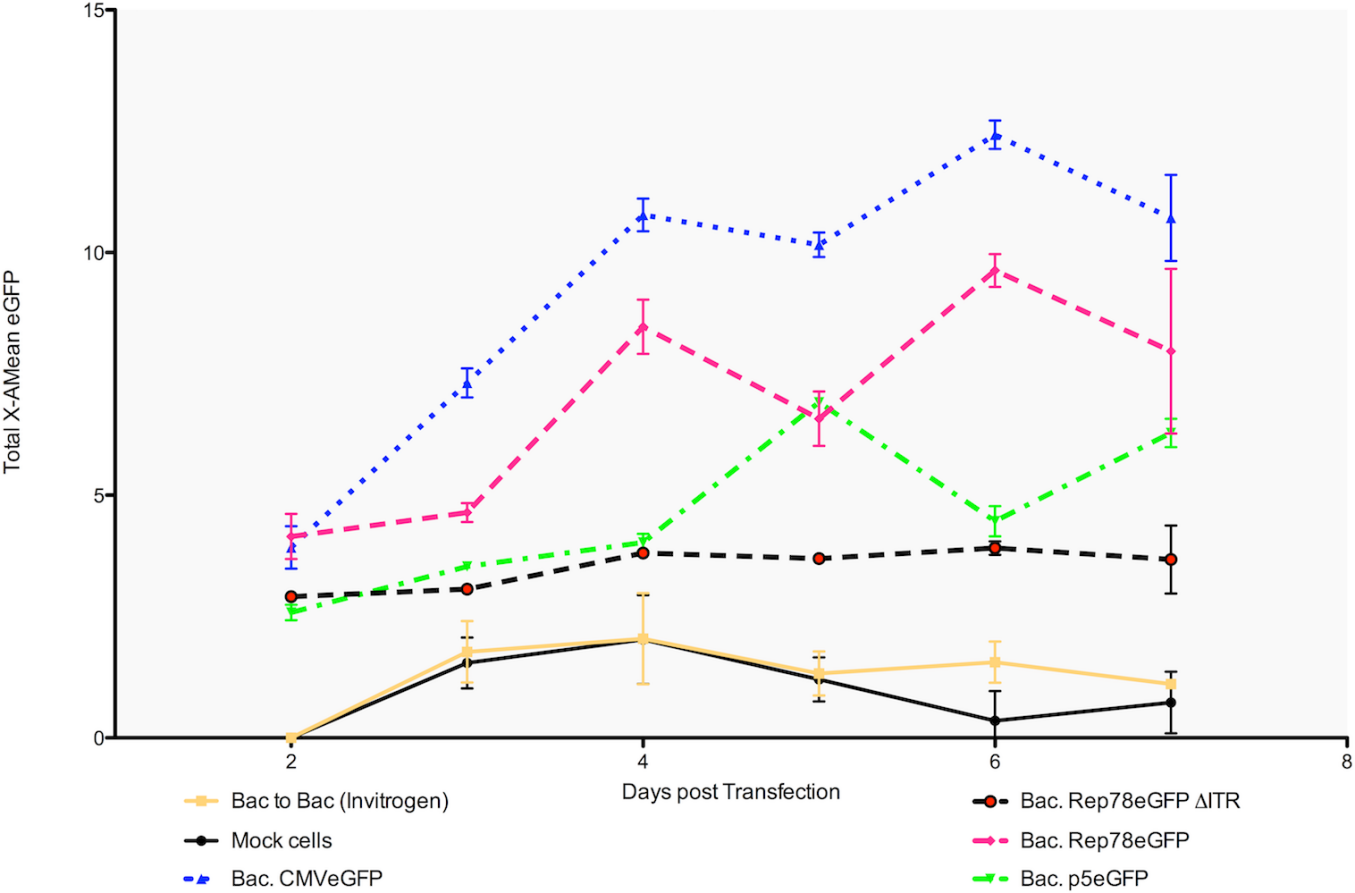
Kinetics of GFP expression by FACS analysis during the seven days following the bacmids transfections. The positive control (blue dots): bacmid with the eGFP protein under the control of CMV promoter. Negative control: cells transfected without bacmid DNA, showing no GFP fluorescence, as for the Bac-to-Bac construct (without any GFP). GFP expression levels were highly similar between the p5eGFP construction and the Rep78eGFP (without ITR), excepted 5 days post transfection. In comparison the Rep78eGFP, within the ITRs context, showed a higher level of GFP expression than the same construct without ITR. This highlights the influence of ITRs on Rep expression via p5 and/or p19 promoters (n = 3). The bars represent the standard deviation.

**Fig 6:**
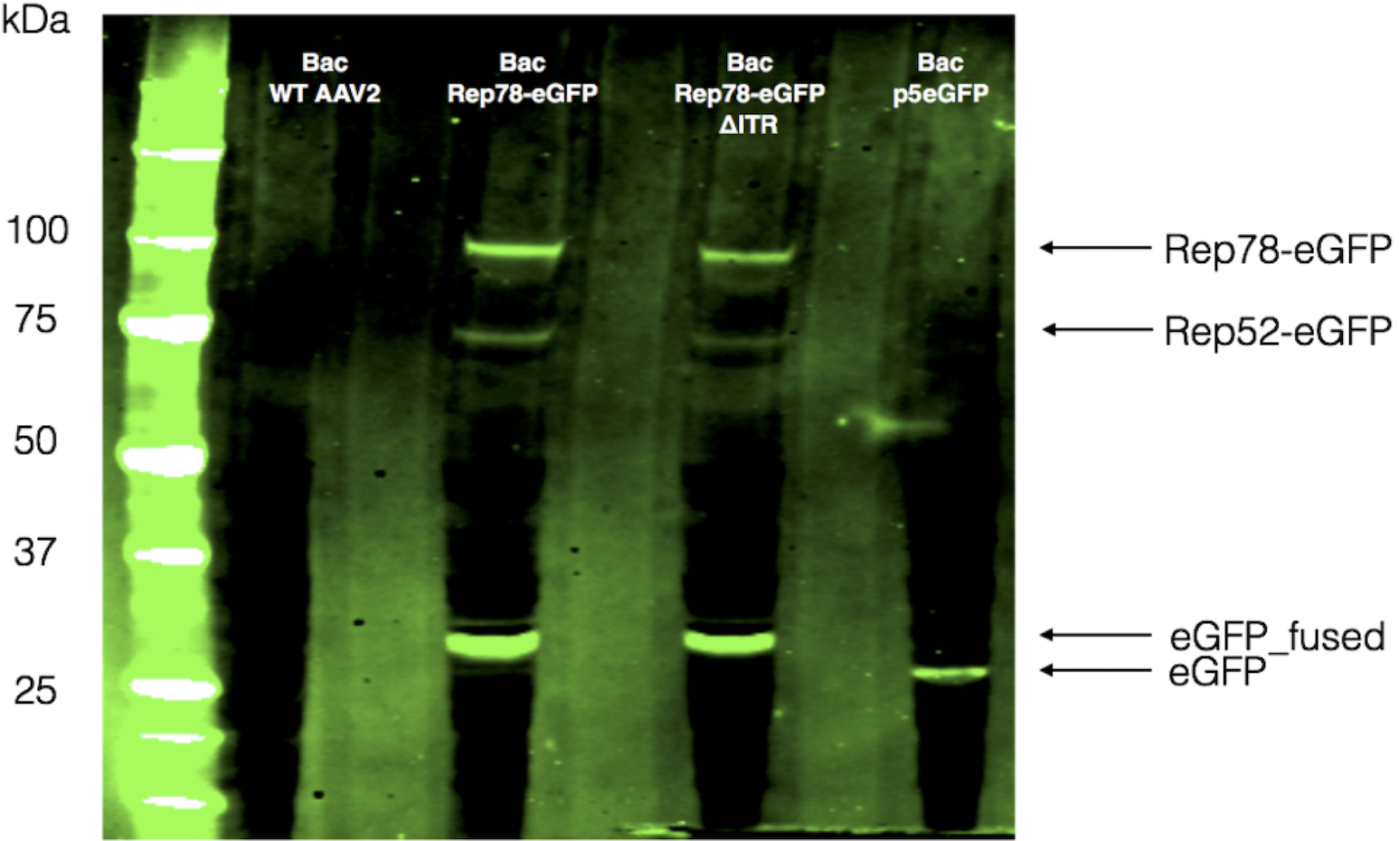
Western blot revealed GFP expression 5 days post-transfection. Sf9 cells were transfected with the different bacmid constructs as indicated at the top of the rows. GFP protein was not detectable, as expected, for the WT AAV2 baculovirus. Rep78eGFP and Rep52eGFP were detected at the expected sizes, respectively. GFP driven by the p5 promoter appeared at 27 kDa, and with the Rep constructs a 32 kDa band was clearly detected.

In mammalian cells, depending on the presence or absence of helper functions, expression of Rep proteins is highly regulated through several auto-activation or auto-inhibition loops [23, 24]. In absence of helper functions, Rep proteins repress the p5 and p19 promoters. On the other hand, in presence of such helper functions the ITR Rep Binding Elements (RBE) activates all promoters. In order to determine if the influence of ITRs on Rep protein expression was the same in the baculovirus/Sf9 cell system than in mammalian cells, Rep78-eGFP ΔITR and the p5-eGFP constructs were used. We performed a kinetic study of Sf9 cells transfected with bacmids carrying the different constructs and followed eGFP expression by FACS analysis during 7 days (Fig 5). All along the experiment, the ITR-containing Rep78-eGFP construct showed a significantly higher level of eGFP expression than Rep78-eGFP ΔITR and p5-eGFP constructs. The eGFP expression profile was comparable for p5-eGFP and Rep78-eGFP ΔITR. Both of them showed low eGFP expression with only weak increase over the experimental time course. Parallel monitoring of cell viability revealed no significant variation between the various constructs. These data suggest that wtAAV2 p5 promoter is naturally active in the baculovirus/Sf9 cell system and that Rep78 protein auto-activates its expression through interaction with the RBE on ITR. However as depicted in Fig 6, the detection of an unknown fused eGFP-peptide within the Rep-eGFP fusion construct, do not allow us direct GFP expression level comparison between the ITR-containing-p5-eGFP construct and both Rep-eGFP (+/- ITRs), in order to detect any Rep proteins influence.

**Fig 7:**
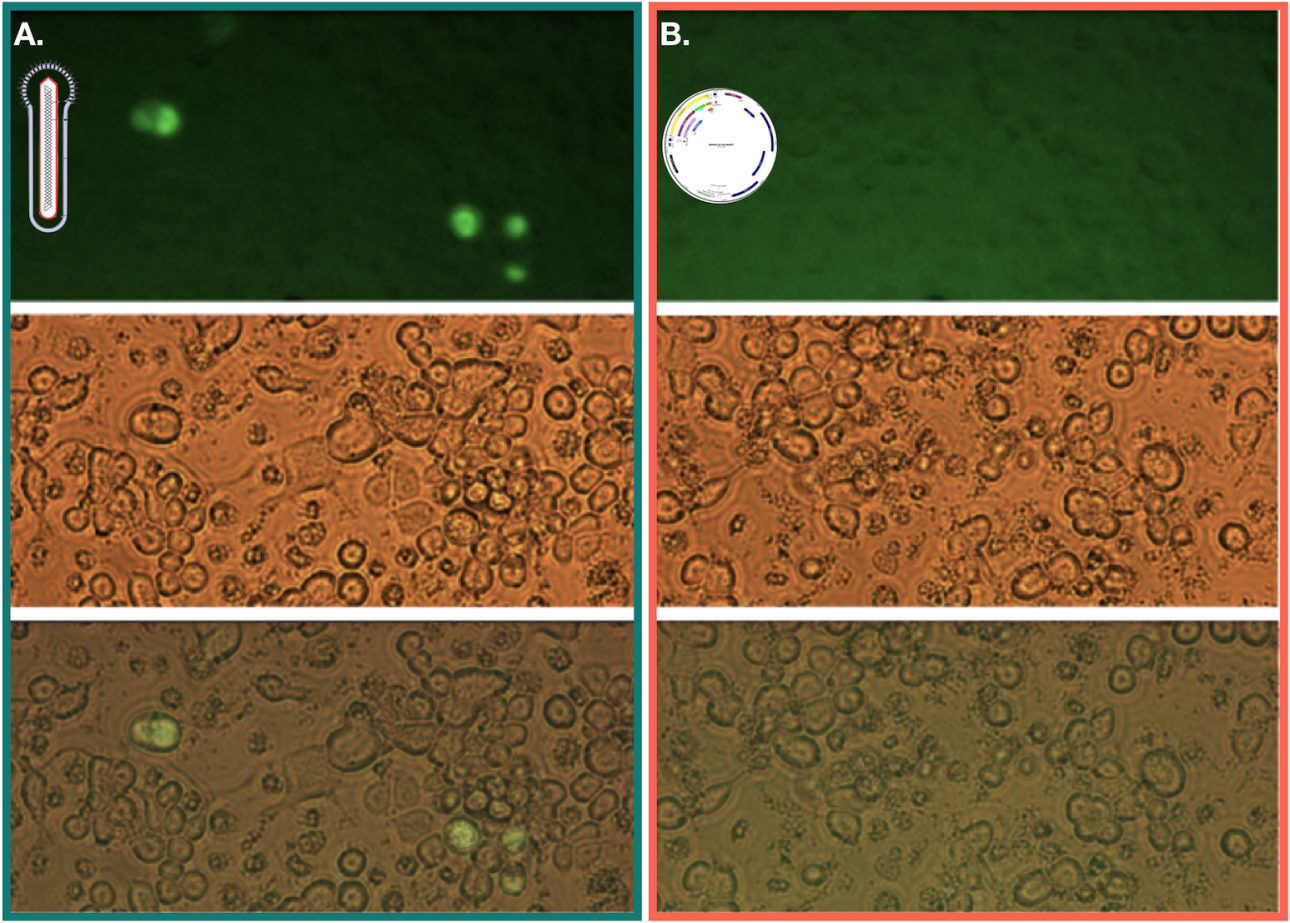
Fluorescent microscopy of transfected Sf9 cells 48 hours post-transfection. **A.** Cells in the have been transfected with a bacmid DNA carrying the ITR-p5-Rep78eGFP-ITR in fluorescent microscopy, light microscopy and merged. **B**. Cells transfected with a plasmid carrying the same construct. eGFP expression is only visible in a baculovirus context.

Regarding the highly regulated Rep protein expression pattern in mammalian cells and the results obtained in this study, we hypothesize that baculovirus brings at least a part of helper functions required by wtAAV2 to replicate. Indeed, to ensure that the p5 and p19 promoters were only active in a baculovirus context, Sf9 cells were also transfected with bacmid DNA carrying the ITR-p5-Rep78eGFP-ITR construct and the same construct harbored in a plasmid. 48 hours post transfection, only the cells transfected with the bacmid DNA were showing signs of eGFP expression (Fig 7), demonstrating the p5 activity only in a baculovirus context and that the baculovirus carries at least, a part of the AAV helper functions as it has been previously suggested [25].

### Transcriptome of WT AAV2 in baculovirus/Sf9 cells context

In order to fully understand these transcription events, we decided to perform the full strand specific RNA-Seq analysis of wtAAV2 genome in the baculovirus context following transfection of Sf9 cells. To this end, baculovirus DNA containing either the Rep2/Cap2 cassette [13] or the wtAAV2 genome were transfected and RNAs were harvested at different time points (0, 7, 24, 30, 40, 48, 68, 80, 94 and 120 hours) post transfection. First, RT-qPCRs were performed for all time points to select the most relevant to analyze in RNA-seq (data not shown). The 48h and 94h post transfection time points were selected and further analyzed.

**Fig 8:**
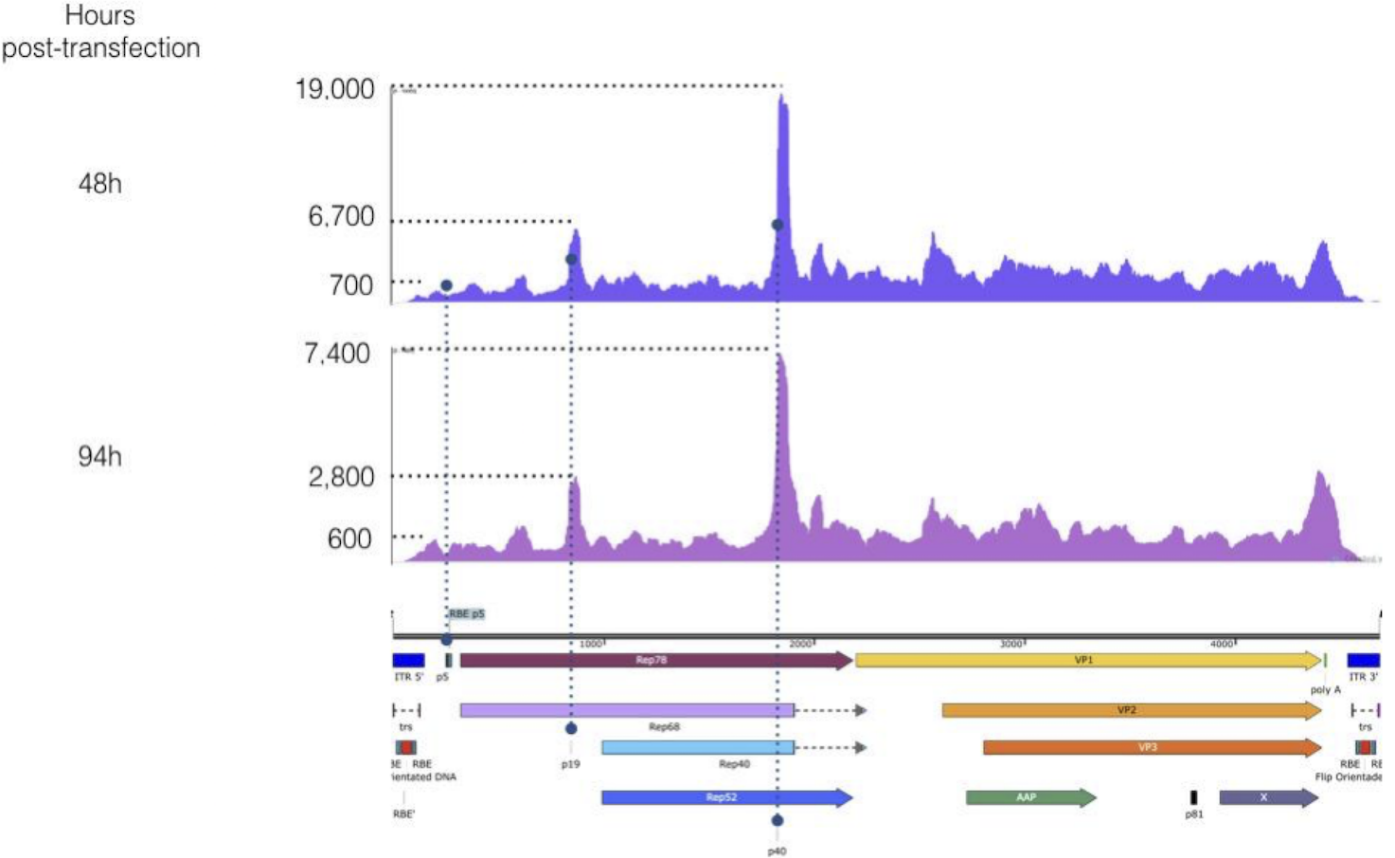
RNA-Seq reads alignment on WT AAV2 positive strand genome 48 and 94 hours post transfection. Peaks of transcription were seen for the sites of the p5, p19 and p40 promoters, with p40 representing the highest level of reads.

RNA-Seq detected an increase of AAV-specific reads near the p5, p19 and p40 promoters for the WT AAV2 genome (Fig 8). These peaks in RNA-Seq data could signify exons or transcriptional stalling along the body of genes due to high GC content or promoter proximal pausing. As no splice sites or GC rich regions were detected, the most plausible explanation for the peaks is promoter proximal pausing that is a common phenomenon at metazoan genes [26]. Our data show evidence that this is also the case for the transcription of AAV genome from the baculovirus system. Overall, the peak height reflects the level of promoter usage. Fig 8 and S3 Table show that p40 is by far the most used promoters of the wtAAV2 sequence in the baculovirus context. Indeed, 48 hours post transfection we detected 4 to 42 times higher level of transcription within the p40 compared to p19 and p5, respectively. Nevertheless, it has to be noticed that gene body counts downstream of p40 were only 1.6 to 2.3 fold higher than those downstream of p19 and p5, respectively. The large discrepancy between the promoter and gene body counts suggests that the p40 promoter exhibits significant promoter proximal pausing but still lead to the highest transcriptional event of all three promoters.

Concerning p5 and p19, these values were surprising, regarding the protein expression ratio between Rep78 and Rep52 detected by western blotting, as Rep52 protein is clearly detected at a lower level than Rep78. However, this could be explained by a less efficient translational Kozak context with AGGTAC for Rep52 compared to GCCGCC for Rep78.

94 hours post-transfection, p40 promoter transcription represents 3 and 22 times that of p5 and p19 promoters, respectively. This decrease in p5 specific reads could be easily explained by the wtAAV2 genome excision (Fig 8). In comparison, there was no significant change in the expression of baculovirus IE1 and DNA polymerase, with RPKM ratios between 48 and 94 hours of 1 for IE1 and 0.8 for DNA Polymerase (S3 Table). Interestingly the p40 promoter was the most transcribed one, but we failed to detect any VP expression from this promoter. This is likely due to inefficient wtAAV RNA splicing in Sf9 cells, as we were unable to detect spliced RNA. Resulting unspliced mRNAs would therefore display longer 5’-end compared to the spliced forms normally observed in mammalian cells [27] where capsid proteins are translated from two spliced forms of the mRNAs originating from p40 promoter. Thus, in insect cells infected with baculovirus encoding wtAAV2 ribosome might not scan up to VP1 start codon if a more favorable Kozak ATG is placed upstream due to longer 5’-UTR sequence.

To verify this hypothesis, we used Rep78eGFP constructs (with and without ITRs) in which the p40 promoter remained at the C-terminal part of Rep, and performed a western blot against eGFP protein to detect any in frame eGFP peptides. As shown in Fig 6, we were able to detect Rep78 and Rep52 proteins, both fused with eGFP, in the two constructs (with and without the ITRs). However, the main product detected was a peptide-eGFP of about 30 kDa, 9 kDa larger than eGFP alone. This size was consistent with the C-terminal part of Rep proteins present after p40 promoter and fused to eGFP.

In the context of wtAAV or rAAV vector production either in the mammalian cells or baculovirus production system, ribosome leaky scanning mechanism functions up to the point where the ribosome encounters an AUG initiation codon. Following this encounter, the ribosome is not able to scan any further and drives the translation from this last favorable AUG start codon. This means that weak start codons can only drive protein expression if placed upstream of this last favorable AUG start codon. As the two splicing events of *cap* mRNA are not performed in insect cells, the first favorable translational initiation element seems to be the AUG codon located in the C-terminal end of the *rep* ORF (position 1,938–1,940 in the of Rep78-eGFPconstruct). In the context of the wtAAV2 we suppose the same mechanism is happening (unfortunately no Rep antibody is available to detect this short Rep C-terminal peptide), making the initiation of translation of VP1 impossible.

Based on the data obtained from the RNA-seq analysis, it is highly probable that the C-terminal part peptide of Rep78 originating from p40 is expressed in the Rep2/Cap2 cassette in baculovirus. This region of Rep78 has previously been described to contain a zinc finger domain between amino acids 537 and 621 of Rep78 (nucleotides 1,929 to 2,186 in the wtAAV sequence) [28, 29]. Nevertheless, the potential influence of high expression level of this Zn-finger containing truncated protein during rAAV production is out of the frame of this paper and remains to be studied.

**Fig 9:**
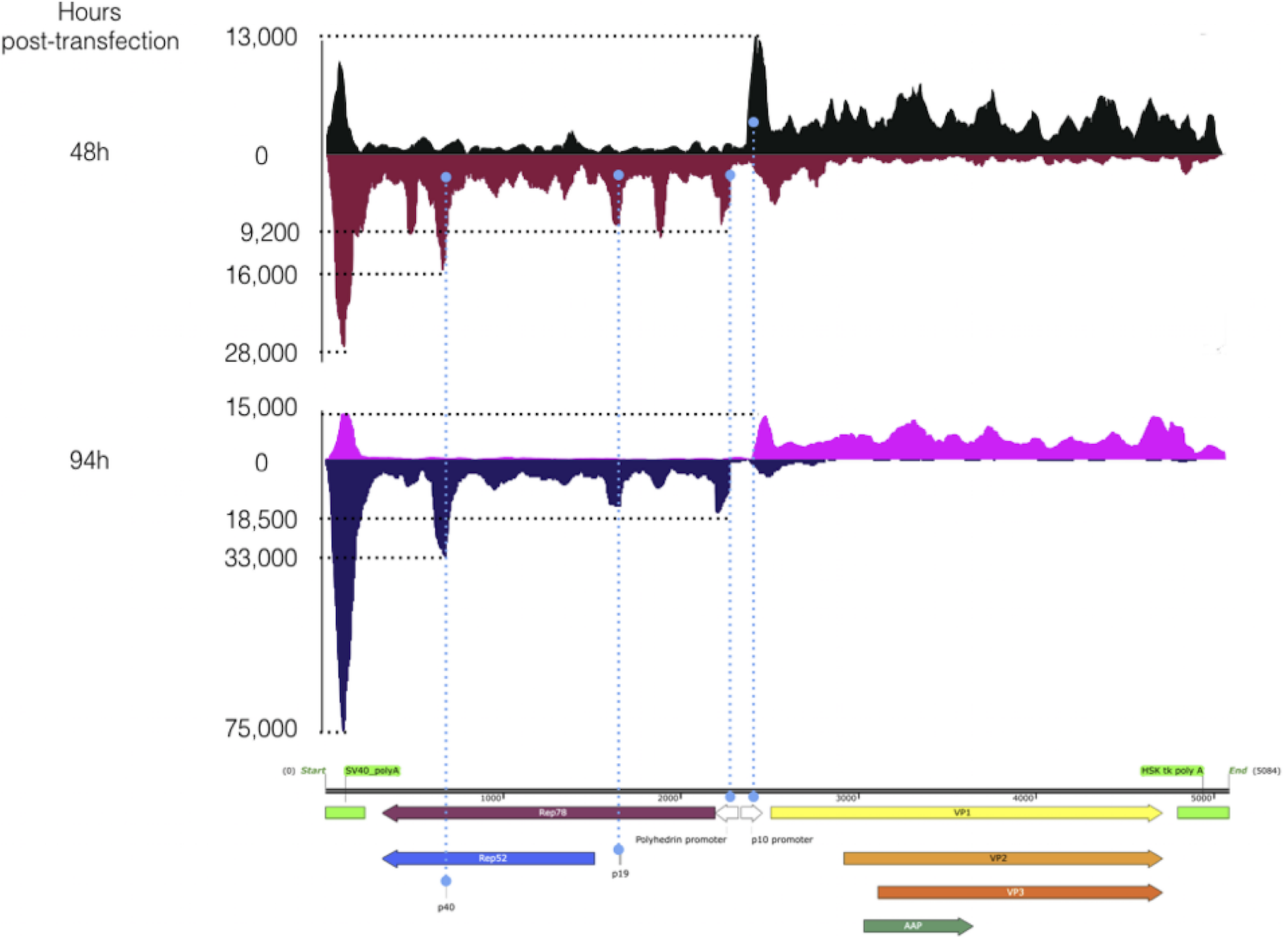
RNA-Seq strand specific reads alignments on Rep/Cap cassette, 48 and 94 hours post transfection. In dark red the reads correspond to *rep* sequence alignments on the negative strand and in black, alignments of the *cap* sequence 48 hours post transfection. Pink color represents the rep mapping 94 hours post transfection and the dark blue the cap alignment 94 hours post transfection. This Rep/Cap cassette used leaky scanning mechanisms to produce all needed proteins. Light blue dots represent the stack of reads aligning to the cassette at specific points. At both time-points we can clearly detect p10 and polh promoters, but also p19 and p40 transcription. Contrary to the WT AAV RNA-Seq the negative strand of both Rep and Cap genes is only less transcribed.

In the Rep2/Cap2 cassette used for rAAV production in the baculovirus/Sf9 cell system the two genes are, unlike in the wtAAV2 sequence, in a head-to-head orientation and under the control of the strong and very late baculoviral promoters polh and p10, respectively. The sequences have only been modified to allow the expression of all proteins required for rAAV production through leaky scanning (VP1, VP2, VP3 and AAP from the *cap* gene, and Rep78 and Rep52 from the *rep* gene). This leaky scanning mechanism allows from a single promoter and from a single mRNA to translate several different proteins. Since these *rep* and *cap* genes are derived from the WT AAV sequence, the native promoters p19 and p40 have been conserved in the *rep* gene. These promoters were assumed to be inactive in a heterologous system. Since then however some residual activity was described for p19 by Kohlbrenner et al. [22] and our RNA-Seq data suggest that wtAAV2 promoters are all active (S3 Table) when present in a baculovirus backbone. Indeed, we discovered that an increase in the transcription signal within p19 and p40 promoters was present in the *rep* sequence similarly to that of wtAAV2 (Fig 9) in addition to the expected transcriptional activities of polyhedrin and p10 promoters. Besides those peaks, the comparison between the wtAAV2 and the Rep2/Cap2 RNA-Seq results leads also to the discovery of an additional peak of transcription located between the polyhedrin and the p19 promoters 48 hours post-transfection. This transcriptional activity remained constant at 94 hours post-transfection. The only differences between the *rep* sequences originating from wtAAV2 and Rep/Cap cassette are the result of the codon optimisation performed in the Rep/Cap cassette to allow the leaky scanning mechanism and avoid unwanted ATG start codon. Whether this represents a region of polymerase stalling or an actual functional promoter leading to the production of a functional RNA transcript that could influence rAAV production remains to be studied.

**Fig 10:**
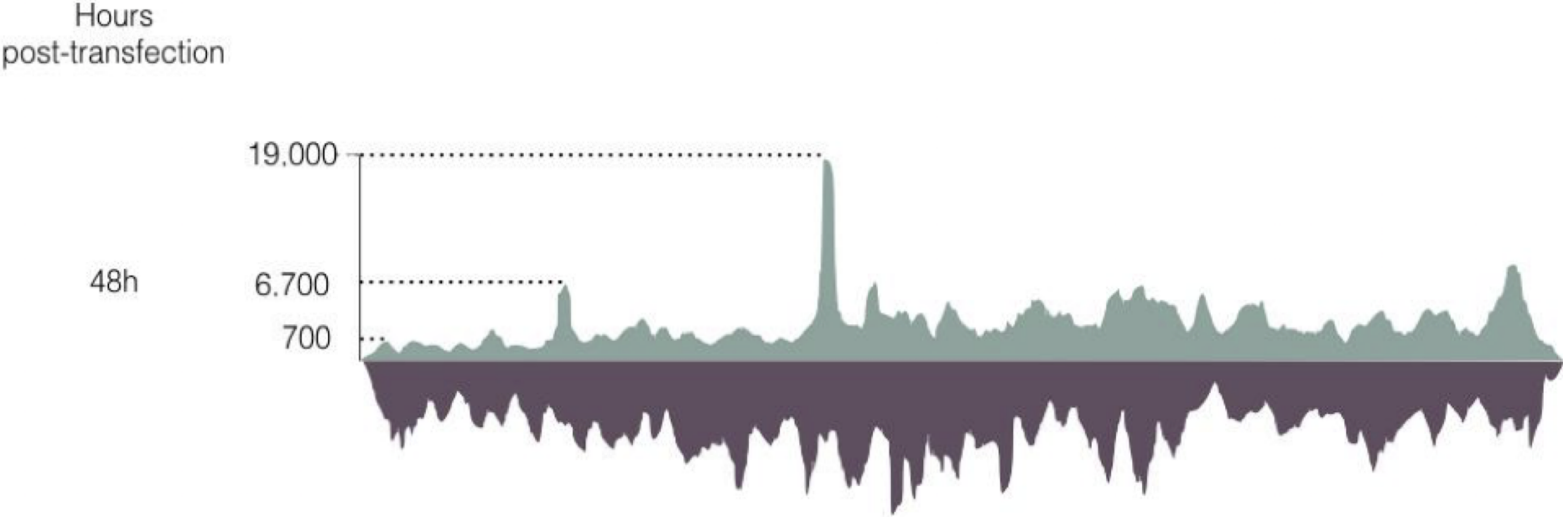
WT AAV2 genome in baculovirus RNA-Seq (strand specific). Dark green represents the reads aligned on the coding strand. Dark grey represents the negative strand. Contrary to the studies performed by Stutika et al. (23), the WT AAV negative strand is highly transcribed in the baculovirus context.

RNA-Seq of wtAAV2 infection of HeLa cells has been performed recently [23], allowing a deeper understanding of fundamental genetics of this single stranded virus. Our work was focused on understanding the behavior of AAV sequences in the baculovirus/Sf9 cell framework. To the contrary of what is observed for wtAAV2 infection of human cells, our analysis detected negative strand reads throughout the whole wtAAV2 genome (Fig 10), which represent 56% of the total reads mapping between the ITRs. The baculovirus dsDNA genome allows transcription from both strands, possibly through regulatory elements within the baculovirus backbone, but it should be noticed that contrary to the wtAAV2 sequence, in the Rep2/Cap2 cassette, the non-coding strands appeared to be weakly transcribed. We have no real explanation for this phenomenon, as the *rep* and *cap* genes from the Rep2/Cap2 cassette originate from the wtAAV2 genome.

To evaluate the influence of baculoviral helper functions on AAV transcription and replication the pGRG25-AAV2wt transposition plasmid was used as a control in our experiment. 48 hours post transfection, no reads were detectable above background (data not show), which validates our previous experiments reporting the baculoviral helper functions necessary for the AAV gene expression.

## Conclusion and perspectives

We describe in this study that wtAAV2 cannot be produced using the baculovirus/Sf9 cell system due to the unanticipated activity of p5 promoter leading to early expression of Rep78 that subsequently results in wtAAV2 genome excision from the baculovirus backbone via the combined action of ITR recognition and endonuclease activity. Thus, it turns out that it was impossible to generate a stable baculovirus stock harboring the wtAAV2 genome as a cassette. By analyzing the expression of Rep78-eGFP fusion protein in the presence or absence of ITRs, we provide evidence that ITRs play a pivotal role on Rep expression in the baculovirus/Sf9 cell system. We also provide proof that the baculovirus is carrying part of the helper functions needed for AAV replication, as the AAV promoters were only active in baculovirus-infected Sf9 cells.

Finally, we performed strand specific RNA-Seq analysis of the wtAAV2 genome and Rep2/Cap2 cassettes in baculovirus. We discovered that not only p5 and p19 were active (as detected by western blotting) but also p40. This promoter appears to be a strong early promoter, leading to the expression of a C-terminal part of Rep78/52. Our analysis also allowed us to discover an unexpected transcriptional event present in the *rep* ORF of the Rep/Cap cassette which is not present in the wtAAV sequence. We hypothesized that it resulted from the codon optimization performed on such sequences to allow correct expression of the replicase proteins.

Interestingly RNA-Seq analysis of the wtAAV2 genome did not allow detection of any splicing events leading either to the expression of Rep68, Rep40 or the two mRNA spliced forms originating from p40 and coding for VP proteins and AAP. Regarding Rep proteins, this result is confirmed by the absence of detection of Rep68 and Rep40 proteins in western blot while the two proteins (Rep78 and Rep52) originating from the non-spliced forms of mRNAs originating from p5 and p19 promoters, could be detected. Since various insect viruses rely on alternative splicing along their life cycle as shown in *Trichoplusia ni* cells infected with AcMNPV baculovirus [30] or for insect parvoviruses of genus densovirinae like *Galleria mellonella* Densovirus or *Acheta domesticus* Densovirus are able to splice NS mRNA [31, 32]. It is more likely that the absence of alternative splicing observed in the case of wtAAV2 in the baculovirus context results from splicing signal sequence divergence than a complete lack of splicing machinery in insect cells.

The baculovirus/Sf9 system is widely used to produce several rAAV serotypes at large scale [12, 13, 25, 33, 34] although further improvements are still required/possible [35]. To do so, our work allows a deeper understanding of AAV sequence behavior in the baculovirus production system and highlights unexpected transcriptional events and peptide expression. The potential influence of such unexpected elements should be further investigated in order to ameliorate the rAAV production field.

## Supporting information

S1 Table Oligo, primers and probes table. Upper case depicts overlapping primers used for Gibson assembly.
(DOCX)

S2 Table Total reads numbers information table. The table depicts the total reads before and after trimming and the reads numbers after mapping on the Sf21 genome for the Rep2/Cap2 cassette at 48 and 94 hours post transfection (R2C2_timing_duplicate) and WT AAV2 genome 48 and 94 hours post transfection (WT_timing_duplicate).
(DOCX)

S3 Table Overall information (position, length, copy number) and RPKM values for baculoviral or cassette genes. The table described all the RPKM values for the Rep2/Cap2 and WT AAV2 construct at 48 and 94 hours post transfection and for each duplicate (Sample_timing_duplicate).
(DOCX)

S4 Table Bacmid genome sequence. (RTF)
