# Supplementary material for: Genetics instability of wtAAV2 genome and AAV promoter activities in the Baculovirus/Sf9 cells system
Source: PLoS One. 2018 Jul 5;13(7):e0199866. doi: 10.1371/journal.pone.0199866 (PMC6033426; doi:10.1371/journal.pone.0199866)
Supplement: S1 Table — Upper case depicts overlapping primers used for Gibson assembly. (DOCX) [file pone.0199866.s001.docx]

**Supplementary Table 1:** Oligo, primers and probes table. Upper case depicts overlapping primers used for Gibson assembly.

| **Primer name** | **Sequence 5’ -> 3’** |
| --- | --- |
| M13 pUC Fw | ccagtcacgacgttgtaaaacg |
| M13 pUC Rv | agcggataacaatttcacacagg |
| Fragment_1_Fw | acgggaactcaacgaccttc |
| Fragment_1_Rv | AGCTCCTCGCCCTTGCTCACttgttcaaagatgcagtcatcc |
| Fragment_2_Fw | gtgagcaagggcgaggagctg |
| Fragment_2_Rv | ATCTCTGTCCTGCCAGACCATGCCttaCTTGTACAGCTCGTCCATGC |
| Rep78eGFP_Fw | gtgaattacgtcatagggttaggg |
| Rep78eGFP_Rv | gaccaaagttcaactgaaacg |
| p5_Fw | tacgtcatagggttagggaggtcctg |
| p5_Rv | AGCTCCTCGCCCTTGCTCACcatggcggctgcgcgttcaaacc |
| eGFP_Fw | gtgagcaagggcgaggagctg |
| eGFP_Rv | ATCTCTGTCCTGCCAGACCATGCCttaCTTGTACAGCTCGTCCATGC |
| Bac_DNA_Pol_Fw (qPCR) | attagcgtggcgtgcttttac |
| Bac_DNA_Pol_Rv (qPCR) | gggtcaggctcctctttgc |
| Bac_DNA_Pol_Probe (qPCR) | caaacacgcgcattaacgagagcacc [5']VIC[3']TAMRA |
| wtAAV_Fw (qPCR) | ctccatcactaggggttccttg |
| wtAAV_Rv (qPCR) | gtagataagtagcatggc |
| wtAAV_Probe (qPCR) | tagttaatgattaaccc [5']6-FAM[3']TAMRA |
| SGC_Fw (qPCR) | aagtcggtcccaaaatggtaga |
| SGC_Rv (qPCR) | tgccgtcgttggagttga |
| SGC_Probe (qPCR) | cagaatcaacagtttcag [5’]6-FAM[3']MGB-NFQ |
