# Supplementary material for: Genetics instability of wtAAV2 genome and AAV promoter activities in the Baculovirus/Sf9 cells system
Source: PLoS One. 2018 Jul 5;13(7):e0199866. doi: 10.1371/journal.pone.0199866 (PMC6033426; doi:10.1371/journal.pone.0199866)
Supplement: S2 Table — The table depicts the total reads before and after trimming and the reads numbers after mapping on the Sf21 genome for the Rep2/Cap2 cassette at 48 and 94 hours post transfection (R2C2_timing_duplicate) and WT AAV2 genome 48 and 94 hours post transfection (WT_timing_duplicate). (DOCX) [file pone.0199866.s002.docx]

**Supplementary Table 2:** Total reads numbers information table. The table depicts the total reads before and after trimming and the reads numbers after mapping on the Sf21 genome for the Rep2/Cap2 cassette at 48 and 94 hours post transfection (R2C2_timing_duplicate) and WT AAV2 genome 48 and 94 hours post transfection (WT_timing_duplicate).

|  | **R2C2_048_01** | **R2C2_048_02** | **R2C2_094_01** | **R2C2_094_02** | **WT_048_01** | **WT_048_02** | **WT_094_01** | **WT_094_02** |
| --- | --- | --- | --- | --- | --- | --- | --- | --- |
| **Total reads before trimming** | 21572939 | 20390105 | 23046709 | 21394720 | 20177448 | 22474774 | 28426114 | 26567432 |
|  |  |  |  |  |  |  |  |  |
| **Reads with adapters** | 4974129 | 6933566 | 5474505 | 2638806 | 5880048 | 2880224 | 5977730 | 5636158 |
| **Reads that were too short** | 2140425 | 4443511 | 3200008 | 1155407 | 4039271 | 1344320 | 2714789 | 2482700 |
| **Reads after trimming** | 19432514 | 15946594 | 19846701 | 20239313 | 16138177 | 21130454 | 25711325 | 24084732 |
| **Reads Mapped** | 12544302 | 11174058 | 11866770 | 11925433 | 11421381 | 15420346 | 16136726 | 16708964 |
| **Reads Unmapped** | 6888414 | 4772769 | 7980217 | 8314129 | 4717055 | 5710467 | 9575151 | 7376176 |
|  |  |  |  |  |  |  |  |  |
| **% Reads with adapters** | 23.06% | 34.00% | 23.75% | 12.33% | 29.14% | 12.82% | 21.03% | 21.21% |
| **% Reads that were too short** | 9.92% | 21.79% | 13.88% | 5.40% | 20.02% | 5.98% | 9.55% | 9.34% |
| **% Reads after trimming** | 90.08% | 78.21% | 86.12% | 94.60% | 79.98% | 94.02% | 90.45% | 90.66% |
| **% Reads Mapped on SF21** | 58.15% | 54.80% | 51.49% | 55.74% | 56.60% | 68.61% | 56.77% | 62.89% |
| **% Reads Unmapped on SF21** | 31.93% | 23.41% | 34.63% | 38.86% | 23.38% | 25.41% | 33.68% | 27.76% |
