# Supplementary material for: Genetics instability of wtAAV2 genome and AAV promoter activities in the Baculovirus/Sf9 cells system
Source: PLoS One. 2018 Jul 5;13(7):e0199866. doi: 10.1371/journal.pone.0199866 (PMC6033426; doi:10.1371/journal.pone.0199866)
Supplement: S3 Table — The table described all the RPKM values for the Rep2/Cap2 and WT AAV2 construct at 48 and 94 hours post transfection and for each duplicate (Sample_timing_duplicate). (DOCX) [file pone.0199866.s003.docx]

**Supplementary Table 3:** Overall information (position, length, copy number) and RPKM values for baculoviral or cassette genes. The table described all the RPKM values for the Rep2/Cap2 and WT AAV2 construct at 48 and 94 hours post transfection and for each duplicate (Sample_timing_duplicate).

| GeneID | chr | start | end | strand | mRNA Length(avg) | Gene Length(avg) | Copies in Genome | R2C2_048_01 genes (Total: 322690.0) RPKM | R2C2_048_02 genes (Total: 543146.0) RPKM | R2C2_094_01 genes (Total: 3111689.0) RPKM | R2C2_094_02 genes (Total: 3663152.0) RPKM | WT_048_01 genes (Total: 452924.0) RPKM | WT_048_02 genes (Total: 552074.0) RPKM | WT_094_01 genes (Total: 3219737.0) RPKM | WT_094_02 genes (Total: 1658404.0) RPKM |
| --- | --- | --- | --- | --- | --- | --- | --- | --- | --- | --- | --- | --- | --- | --- | --- |
| Ac-p6.9 | Bacmid_dCC | 101773 | 101940 | - | 167 | 167 | 1 | 95455.06588 | 100986.2899 | 137653.6438 | 135498.9619 | 80594.08204 | 97953.97775 | 65516.5648 | 111210.0174 |
| Ac-odv-ec27 | Bacmid_dCC | 138523 | 139395 | + | 872 | 872 | 1 | 71062.60702 | 56694.84445 | 52808.79332 | 68981.98976 | 50226.64555 | 57128.19446 | 49689.93806 | 49895.9882 |
| AcOrf-73 | Bacmid_dCC | 77076 | 77375 | - | 299 | 299 | 1 | 17764.54638 | 48349.55899 | 97338.22616 | 28255.72532 | 70009.60448 | 55340.11648 | 121298.2598 | 33733.12161 |
| Ac-odv-e18 | Bacmid_dCC | 138319 | 138507 | + | 188 | 188 | 1 | 48511.74601 | 45303.44139 | 44808.73605 | 54577.46004 | 47234.45218 | 45900.41468 | 36396.1933 | 43444.10188 |
| AcOrf-75 | Bacmid_dCC | 78187 | 78588 | - | 401 | 401 | 1 | 29358.87527 | 33796.82163 | 51189.81461 | 37071.35412 | 35722.44156 | 38517.19 | 58475.83451 | 40809.29872 |
| Ac-39K_pp31 | Bacmid_dCC | 44303 | 45130 | - | 827 | 827 | 1 | 34901.59037 | 44981.79269 | 27607.02435 | 20733.29581 | 49670.53419 | 36965.14775 | 33802.99676 | 26406.1226 |
| Ac-gp16 | Bacmid_dCC | 123690 | 124010 | + | 320 | 320 | 1 | 25818.12266 | 36442.77966 | 42820.39433 | 25162.75601 | 56714.81308 | 35400.59847 | 46896.29929 | 18040.68852 |
| AcOrf-76 | Bacmid_dCC | 78604 | 78858 | - | 254 | 254 | 1 | 25401.62507 | 36742.77803 | 40873.31329 | 33988.19842 | 40941.32147 | 34615.35269 | 45517.72959 | 40789.6202 |
| AcOrf-74 | Bacmid_dCC | 77372 | 78169 | - | 797 | 797 | 1 | 19456.89219 | 29277.82401 | 40641.6711 | 31725.97369 | 39445.35187 | 29120.25745 | 52286.11943 | 34584.50508 |
| AcOrf-81 | Bacmid_dCC | 81887 | 82588 | - | 701 | 701 | 1 | 22386.70477 | 26080.42384 | 28716.44094 | 20717.17388 | 24425.21808 | 27761.99599 | 35259.96608 | 30964.92398 |
| Ac-TLP | Bacmid_dCC | 82437 | 82979 | - | 542 | 542 | 1 | 32424.61691 | 34189.90603 | 22706.31209 | 24327.7473 | 30881.74809 | 26231.1752 | 35191.15787 | 35510.73436 |
| Ac-v-ubi | Bacmid_dCC | 44023 | 44256 | + | 233 | 233 | 1 | 9496.351552 | 10801.79692 | 20673.81159 | 8591.536197 | 14318.02806 | 18300.09073 | 13811.00728 | 10268.93502 |
| AcOrf-132 | Bacmid_dCC | 125039 | 125698 | + | 659 | 659 | 1 | 16110.77517 | 20204.8866 | 22135.43961 | 18333.75528 | 20185.81564 | 18237.20241 | 20261.97784 | 15642.95363 |
| Ac-odv-e25 | Bacmid_dCC | 95032 | 95718 | + | 686 | 686 | 1 | 22243.77131 | 17858.38146 | 18869.41254 | 26877.47279 | 18618.89546 | 18224.41283 | 15898.20741 | 22659.56739 |
| Ac-cg30 | Bacmid_dCC | 89798 | 90592 | - | 794 | 794 | 1 | 13828.18378 | 16470.422 | 27583.89213 | 22535.34056 | 18775.28755 | 17273.99575 | 29495.73641 | 20144.70566 |
| AcOrf-78 | Bacmid_dCC | 80019 | 80348 | - | 329 | 329 | 1 | 10267.03619 | 15557.23143 | 27122.94715 | 14556.36797 | 13998.87295 | 17072.95021 | 26451.59317 | 16247.72296 |
| Ac-lef11 | Bacmid_dCC | 45124 | 45462 | - | 338 | 338 | 1 | 22554.48421 | 18994.09744 | 9653.426656 | 13705.17576 | 18179.04949 | 16114.59642 | 10988.98972 | 16168.32175 |
| Ac-p10 | Bacmid_dCC | 132005 | 132289 | + | 284 | 284 | 1 | 25129.86126 | 16907.23782 | 16542.57611 | 31427.37168 | 14809.87203 | 16015.15249 | 12680.37259 | 24888.17435 |
| Ac-IE-01 | Bacmid_dCC | 135998 | 142112 | + | 6114 | 6114 | 1 | 18010.29948 | 15259.60062 | 14294.08187 | 19497.14371 | 13819.26665 | 15356.19495 | 13609.57968 | 15788.87355 |
| Ac-lef3 | Bacmid_dCC | 72782 | 73939 | - | 1157 | 1157 | 1 | 15272.43716 | 16571.72161 | 6626.809569 | 6268.364134 | 17893.90894 | 15345.60605 | 12849.11982 | 12295.87593 |
| Ac-bro | Bacmid_dCC | 1040 | 2027 | - | 987 | 987 | 1 | 16113.28127 | 13451.22253 | 15045.07772 | 14776.52927 | 14779.57072 | 15279.95093 | 15539.2883 | 13623.16128 |
| p19 | Bacmid_dCC | 7944 | 7949 | - | 5 | 5 | 1 | 21692.64619 | 27616.88386 | 53861.42381 | 36252.9319 | 32234.98865 | 14490.81101 | 14845.93307 | 9286.036454 |
| AcOrf-122 | Bacmid_dCC | 117774 | 117962 | - | 188 | 188 | 1 | 5340.74268 | 10508.12638 | 8781.233627 | 3319.429406 | 8349.998882 | 14009.06863 | 8724.447224 | 6809.289649 |
| Ac-vp39 | Bacmid_dCC | 90595 | 91638 | - | 1043 | 1043 | 1 | 14835.14347 | 12868.4617 | 17857.07888 | 24878.37508 | 12561.39578 | 13919.44514 | 16403.20252 | 22904.34637 |
| AcOrf-47 | Bacmid_dCC | 53999 | 54265 | - | 266 | 266 | 1 | 8900.742047 | 15739.54734 | 7988.312089 | 3406.204168 | 13952.77988 | 13816.66144 | 12265.74754 | 8176.62667 |
| Ac-alk-exo | Bacmid_dCC | 125726 | 126985 | + | 1259 | 1259 | 1 | 19036.75544 | 15147.24294 | 14168.05364 | 17735.82506 | 13210.42845 | 13682.24908 | 11907.5434 | 12881.17504 |
| Ac-PE_pp34 | Bacmid_dCC | 124069 | 124827 | + | 758 | 758 | 1 | 19648.48428 | 15659.28322 | 12938.70105 | 19881.73445 | 12425.85586 | 13506.27702 | 9639.998104 | 14902.11089 |
| AcOrf-124 | Bacmid_dCC | 118854 | 119597 | + | 743 | 743 | 1 | 11094.4893 | 11802.53188 | 12237.104 | 11794.73905 | 13223.48306 | 12889.11807 | 11014.6679 | 11604.50222 |
| AcOrf-56 | Bacmid_dCC | 61695 | 61949 | + | 254 | 254 | 1 | 5392.660077 | 10379.88916 | 7798.888814 | 3439.230804 | 9283.509837 | 12643.80311 | 11061.20569 | 6654.248947 |
| Ac-ctx | Bacmid_dCC | 2083 | 2245 | - | 162 | 162 | 1 | 10999.35764 | 11251.32305 | 11210.21929 | 16050.7389 | 10180.76126 | 12299.29947 | 9499.664026 | 19262.16075 |
| Ac-lef2 | Bacmid_dCC | 3088 | 3721 | + | 633 | 633 | 1 | 6193.003258 | 9793.159342 | 10683.35748 | 6033.357569 | 14890.08273 | 12018.44515 | 13076.92691 | 7176.811991 |
| AcOrf-58 | Bacmid_dCC | 62635 | 62808 | - | 173 | 173 | 1 | 6197.898912 | 7556.064562 | 12089.41418 | 5212.037488 | 11651.96953 | 11988.42385 | 9053.624261 | 5357.194666 |
| Ac-p40 | Bacmid_dCC | 101982 | 103067 | - | 1085 | 1085 | 1 | 14626.4702 | 11664.42148 | 11312.48067 | 16596.97305 | 11147.22984 | 11667.77398 | 8270.418183 | 9021.499178 |
| AcOrf-117 | Bacmid_dCC | 114971 | 115258 | + | 287 | 287 | 1 | 5377.271182 | 11277.69613 | 14060.72819 | 6532.711061 | 13639.59593 | 11436.12786 | 16320.28899 | 9036.436669 |
| Ac-49K | Bacmid_dCC | 136798 | 138231 | + | 1433 | 1433 | 1 | 12514.73866 | 11360.24597 | 9524.225292 | 13975.20517 | 9919.263971 | 10469.93961 | 8194.832641 | 11685.28617 |
| AcOrf-102 | Bacmid_dCC | 103087 | 103455 | - | 368 | 368 | 1 | 15873.6949 | 11076.7719 | 10153.68461 | 15656.06436 | 9407.471375 | 10095.33064 | 7126.561012 | 9500.359854 |
| Ac-35K_p35 | Bacmid_dCC | 129658 | 130557 | + | 899 | 899 | 1 | 11265.14663 | 9314.170396 | 5315.991738 | 5914.962915 | 8573.63169 | 9826.430105 | 6728.521985 | 8965.694154 |
| AcOrf-79 | Bacmid_dCC | 80351 | 80665 | - | 314 | 314 | 1 | 11744.42628 | 10114.46383 | 9948.106643 | 14599.69221 | 8304.144838 | 9593.239935 | 9163.22815 | 13294.57159 |
| AcOrf-60 | Bacmid_dCC | 63164 | 63427 | - | 263 | 263 | 1 | 8012.49289 | 9443.644189 | 8329.930173 | 8460.581335 | 10804.32064 | 9538.865616 | 7835.465096 | 8602.342792 |
| AcOrf-48 | Bacmid_dCC | 54339 | 54680 | - | 341 | 341 | 1 | 7470.194877 | 9211.027148 | 3542.597095 | 3130.166496 | 10961.68316 | 9083.316288 | 5887.441033 | 7080.262399 |
| AcOrf-93 | Bacmid_dCC | 94538 | 95023 | + | 485 | 485 | 1 | 10146.66337 | 8715.926507 | 8591.482188 | 13485.09102 | 7524.987465 | 8892.428099 | 7890.764235 | 12039.89517 |
| Ac-p48 | Bacmid_dCC | 103436 | 104599 | - | 1163 | 1163 | 1 | 10234.79352 | 9413.011833 | 8848.835385 | 12738.9364 | 8087.318558 | 8552.132769 | 6174.299848 | 8983.660352 |
| Ac_gp64 | Bacmid_dCC | 121345 | 122823 | - | 1478 | 1478 | 1 | 14444.29148 | 9356.356096 | 8682.178122 | 12463.84777 | 7939.689171 | 8379.032045 | 7246.618492 | 8967.325153 |
| AcOrf-70 | Bacmid_dCC | 75171 | 76043 | + | 872 | 872 | 1 | 3610.702577 | 6306.699701 | 7735.334132 | 3543.534253 | 7492.092766 | 7883.117517 | 10172.70677 | 6755.970463 |
| Ac-FP | Bacmid_dCC | 63574 | 64218 | - | 644 | 644 | 1 | 6135.342479 | 7141.50889 | 8761.793083 | 6694.163696 | 11173.08645 | 7748.871185 | 10077.59484 | 6959.658482 |
| AcOrf-55 | Bacmid_dCC | 61472 | 61693 | + | 221 | 221 | 1 | 7417.847341 | 9213.958838 | 3449.262338 | 3945.36964 | 9500.859793 | 7679.801991 | 6997.283245 | 5568.784276 |
| Ac-lef6 | Bacmid_dCC | 38526 | 39047 | + | 521 | 521 | 1 | 9195.731016 | 7216.081489 | 4736.644161 | 4214.299587 | 7229.628302 | 7659.130678 | 5034.924417 | 5705.830411 |
| AcOrf-59 | Bacmid_dCC | 62943 | 63152 | - | 209 | 209 | 1 | 9089.263237 | 7285.219442 | 5820.005742 | 7331.5165 | 8651.915745 | 7522.741603 | 6025.931272 | 6439.580366 |
| AcOrf-13 | Bacmid_dCC | 24699 | 25682 | - | 983 | 983 | 1 | 9700.373832 | 7259.616265 | 5215.460526 | 6334.565449 | 7912.865635 | 7479.425471 | 5131.435214 | 6159.938307 |
| AcOrf-111 | Bacmid_dCC | 111209 | 111412 | - | 203 | 203 | 1 | 2763.102717 | 5795.464297 | 5775.141201 | 1765.687117 | 7471.974223 | 7450.632511 | 6093.87961 | 3199.111145 |
| Ac-gp41 | Bacmid_dCC | 80668 | 81897 | - | 1229 | 1229 | 1 | 9448.139636 | 6631.947103 | 8230.338005 | 12134.79175 | 6866.152826 | 7260.143924 | 7434.060638 | 9927.000918 |
| AcOrf-25 | Bacmid_dCC | 36244 | 37194 | - | 950 | 950 | 1 | 8122.509627 | 8440.107312 | 2664.317133 | 2434.774033 | 6465.59037 | 6721.066951 | 5492.439453 | 4755.999395 |
| AcOrf-45 | Bacmid_dCC | 51216 | 51794 | + | 578 | 578 | 1 | 5474.095839 | 7176.567397 | 4528.632361 | 3282.00723 | 6222.543076 | 6214.376782 | 5764.080585 | 5761.782607 |
| Ac-IE-1 | Bacmid_dCC | 140364 | 142112 | + | 1748 | 1748 | 1 | 5873.466561 | 5826.720118 | 5659.070599 | 7838.437151 | 5168.551674 | 6165.641128 | 6876.387787 | 8203.484239 |
| AcOrf-19 | Bacmid_dCC | 30522 | 30848 | + | 326 | 326 | 1 | 3441.164733 | 4845.66183 | 5997.571315 | 3287.590792 | 5323.283696 | 6100.809238 | 4461.554057 | 3281.298038 |
| Ac-IAP1 | Bacmid_dCC | 37661 | 38521 | + | 860 | 860 | 1 | 4587.165881 | 4971.039094 | 4084.747903 | 3593.931204 | 7776.344427 | 5907.9542 | 4807.557191 | 4394.10982 |
| AcOrf-150 | Bacmid_dCC | 143622 | 143921 | + | 299 | 299 | 1 | 1782.673266 | 4242.593752 | 8444.800227 | 2813.886049 | 7140.521837 | 5640.027197 | 11270.36942 | 3803.471475 |
| AcOrf-5 | Bacmid_dCC | 2778 | 3108 | + | 330 | 330 | 1 | 5390.293902 | 3927.734593 | 3538.953029 | 5198.371305 | 4489.347735 | 5219.985331 | 3960.421349 | 5496.339562 |
| Ac-gp37 | Bacmid_dCC | 66344 | 67252 | - | 908 | 908 | 1 | 5563.092085 | 4501.430409 | 3746.000216 | 6084.22608 | 4328.214939 | 5146.791357 | 3559.406381 | 4867.080581 |
| Ac-p24 | Bacmid_dCC | 123066 | 123662 | + | 596 | 596 | 1 | 4445.640579 | 5372.009064 | 5439.545693 | 5042.050851 | 6493.970661 | 4941.706944 | 5231.991801 | 4825.938496 |
| Ac-vlf-1 | Bacmid_dCC | 78874 | 80013 | - | 1139 | 1139 | 1 | 3468.973272 | 4888.115703 | 6528.387252 | 5298.957494 | 6144.834692 | 4866.317132 | 8289.521479 | 6593.707401 |
| Ac-pkip | Bacmid_dCC | 35695 | 36204 | - | 509 | 509 | 1 | 3366.835067 | 4459.936843 | 4972.69406 | 3229.743472 | 5040.376889 | 4761.469827 | 4082.75058 | 3784.972632 |
| Ac-p26 | Bacmid_dCC | 131210 | 131932 | + | 722 | 722 | 1 | 6348.124994 | 4549.263232 | 2423.619856 | 3300.443908 | 5379.021842 | 4691.450242 | 4124.921829 | 4696.134743 |
| Ac-pcna | Bacmid_dCC | 54704 | 55474 | - | 770 | 770 | 1 | 8540.221748 | 5501.857122 | 1484.97463 | 2557.941868 | 5015.032481 | 4681.284727 | 2602.858395 | 4556.093784 |
| ORF603 | Bacmid_dCC | 3758 | 4364 | - | 606 | 606 | 1 | 2173.355642 | 3803.777623 | 6317.603026 | 3266.407328 | 5239.151146 | 4638.972503 | 5309.660252 | 2727.382658 |
| Ac-fgf | Bacmid_dCC | 42102 | 42647 | - | 545 | 545 | 1 | 4361.273822 | 4587.612022 | 3740.261996 | 2917.72588 | 4849.22476 | 4636.394809 | 4956.242751 | 3714.193301 |
| AcOrf-107 | Bacmid_dCC | 109120 | 109452 | + | 332 | 332 | 1 | 2044.186539 | 4397.628293 | 4224.258373 | 1984.103387 | 6823.134667 | 4577.481341 | 4658.765607 | 2257.577709 |
| Ac-PE38 | Bacmid_dCC | 145692 | 146657 | + | 965 | 965 | 1 | 2482.370911 | 3447.579215 | 2760.109071 | 2088.577597 | 4948.845231 | 4548.087447 | 6445.671766 | 4410.258078 |
| Ac-helicase | Bacmid_dCC | 95755 | 99420 | - | 3665 | 3665 | 1 | 5068.240939 | 4747.240609 | 4006.106382 | 4869.705661 | 4649.491532 | 4510.833292 | 5217.732737 | 4921.972796 |
| AcOrf-29 | Bacmid_dCC | 39107 | 39322 | - | 215 | 215 | 1 | 1729.646208 | 2680.336325 | 2479.771941 | 745.3233058 | 3399.102021 | 4389.367755 | 3579.665481 | 1705.197875 |
| Ac-IE-0 | Bacmid_dCC | 135998 | 136783 | + | 785 | 785 | 1 | 5668.906266 | 4406.974503 | 3926.022334 | 5758.154079 | 3917.925067 | 4314.938949 | 3746.79493 | 5248.696095 |
| AcOrf-145 | Bacmid_dCC | 139465 | 139698 | + | 233 | 233 | 1 | 4561.972805 | 4029.93155 | 5063.283897 | 9401.130021 | 4074.620824 | 4306.818295 | 5430.561111 | 8441.851322 |
| Ac-HE65 | Bacmid_dCC | 106728 | 108389 | - | 1661 | 1661 | 1 | 4330.320101 | 4678.742395 | 2138.525105 | 2036.158965 | 3999.698187 | 4238.845757 | 2834.71349 | 2986.630723 |
| AcOrf-44 | Bacmid_dCC | 50819 | 51214 | + | 395 | 395 | 1 | 7123.661028 | 5467.443842 | 1986.791894 | 2973.160612 | 3923.870649 | 4150.058218 | 2972.960804 | 4263.669212 |
| AcOrf-118 | Bacmid_dCC | 115292 | 115765 | - | 473 | 473 | 1 | 3747.566784 | 3674.466295 | 2967.736269 | 1957.671251 | 3211.455906 | 4128.196161 | 3457.13896 | 3464.958003 |
| ccdB | Bacmid_dCC | 19022 | 19327 | - | 305 | 305 | 1 | 6583.997533 | 4237.607097 | 2366.539338 | 4035.761158 | 4118.955433 | 4062.17817 | 1848.231602 | 2991.222728 |
| AcOrf-108 | Bacmid_dCC | 109453 | 109770 | - | 317 | 317 | 1 | 1476.155735 | 3176.958038 | 5355.810025 | 1852.363472 | 3398.875256 | 3931.261032 | 3855.361233 | 1776.631171 |
| Ac-94K | Bacmid_dCC | 127036 | 129447 | - | 2411 | 2411 | 1 | 5138.780558 | 4434.432312 | 1991.260745 | 2484.980662 | 3486.26449 | 3893.917124 | 3310.924751 | 4113.883166 |
| AcOrf-72 | Bacmid_dCC | 76885 | 77067 | + | 182 | 182 | 1 | 1447.311559 | 2164.83998 | 6635.737584 | 1835.925652 | 3032.796614 | 3871.514756 | 5491.541291 | 2604.118971 |
| Ac-vp80 | Bacmid_dCC | 104625 | 106700 | + | 2075 | 2075 | 1 | 3727.700165 | 3896.087936 | 3908.619929 | 4003.136202 | 4556.204701 | 3815.622587 | 3753.206349 | 3063.186051 |
| Ac-egt | Bacmid_dCC | 26487 | 28007 | + | 1520 | 1520 | 1 | 5154.042253 | 4572.532305 | 1996.081296 | 1943.609449 | 3237.733855 | 3485.659722 | 2291.377084 | 2928.862233 |
| Ac-38K | Bacmid_dCC | 100082 | 101044 | - | 962 | 962 | 1 | 2631.852085 | 2851.639428 | 4768.085222 | 5665.23004 | 2912.468359 | 3408.051967 | 4128.964196 | 4684.136838 |
| AcOrf-97 | Bacmid_dCC | 99900 | 100070 | + | 170 | 170 | 1 | 1312.49624 | 2350.142666 | 2673.032896 | 1295.89458 | 2792.313683 | 3324.362527 | 2705.737986 | 1929.566016 |
| Ac-p15 | Bacmid_dCC | 89417 | 89797 | + | 380 | 380 | 1 | 2128.488969 | 2785.913723 | 1942.590292 | 1371.410253 | 3335.054702 | 3317.633048 | 4259.092558 | 2405.610264 |
| Ac-odv-e56 | Bacmid_dCC | 142174 | 143304 | - | 1130 | 1130 | 1 | 2909.721569 | 3188.568832 | 3316.924968 | 3500.056492 | 3411.461401 | 3297.300692 | 3161.638984 | 3414.627472 |
| AcOrf-63 | Bacmid_dCC | 65856 | 66323 | + | 467 | 467 | 1 | 2315.91726 | 2365.471851 | 2288.118879 | 1785.827039 | 2562.459865 | 3285.256137 | 2862.430717 | 2860.003163 |
| AcOrf-84 | Bacmid_dCC | 86226 | 86792 | + | 566 | 566 | 1 | 2031.290191 | 3116.251441 | 2015.085062 | 1796.614523 | 2672.075999 | 3225.86959 | 3332.471559 | 4924.058154 |
| AcOrf-120 | Bacmid_dCC | 117357 | 117605 | + | 248 | 248 | 1 | 2186.758689 | 2293.983095 | 3516.915857 | 2357.831937 | 3204.981196 | 3206.38409 | 3768.340245 | 2963.887316 |
| AcOrf-121 | Bacmid_dCC | 117708 | 117884 | + | 176 | 176 | 1 | 1091.675377 | 1841.125591 | 3984.243693 | 1594.503611 | 1768.809698 | 3190.448448 | 3144.666785 | 1846.654977 |
| Ac-ptp | Bacmid_dCC | 502 | 1009 | + | 507 | 507 | 1 | 2108.752588 | 2658.19316 | 2646.380793 | 2812.809169 | 3853.98462 | 3161.826368 | 3254.703704 | 2917.421991 |
| AcOrf-109 | Bacmid_dCC | 109782 | 110954 | - | 1172 | 1172 | 1 | 3781.141401 | 3756.084716 | 3152.540939 | 3835.820774 | 3545.411698 | 3160.591779 | 2549.600905 | 2936.229004 |
| Ac-lef1 | Bacmid_dCC | 25574 | 26374 | - | 800 | 800 | 1 | 4264.929189 | 3106.899434 | 2060.376214 | 2874.914827 | 2994.431737 | 3120.052747 | 1975.316617 | 2922.99102 |
| AcOrf-66 | Bacmid_dCC | 70353 | 72779 | + | 2426 | 2426 | 1 | 2562.445428 | 2600.798598 | 2453.848755 | 2765.44778 | 2999.653373 | 3100.053964 | 3189.826982 | 2878.739901 |
| Ac-lef10 | Bacmid_dCC | 60189 | 60425 | + | 236 | 236 | 1 | 3007.031464 | 2730.482867 | 1826.083323 | 2431.452383 | 2123.677276 | 3070.087079 | 1864.822278 | 3273.005915 |
| AcOrf-26 | Bacmid_dCC | 37270 | 37659 | + | 389 | 389 | 1 | 4620.541606 | 3838.439213 | 1972.824881 | 3292.709846 | 2917.347637 | 3068.587551 | 2346.548778 | 4010.111893 |
| Ac-ME53 | Bacmid_dCC | 134371 | 135720 | - | 1349 | 1349 | 1 | 2765.852591 | 2714.60252 | 1857.697927 | 1961.513598 | 2582.674741 | 3049.354319 | 2349.988661 | 2776.701282 |
| AcOrf-43 | Bacmid_dCC | 50605 | 50838 | + | 233 | 233 | 1 | 5599.389361 | 3856.091366 | 1725.461225 | 2554.145494 | 2691.144917 | 3016.327615 | 1795.524255 | 3633.463904 |
| Ac-HisP | Bacmid_dCC | 42794 | 43342 | - | 548 | 548 | 1 | 3602.245992 | 2939.753452 | 1993.894493 | 2799.132997 | 2977.409336 | 2997.984852 | 2496.010187 | 3473.791009 |
| AcOrf-16 | Bacmid_dCC | 28153 | 28830 | + | 677 | 677 | 1 | 2632.047175 | 3540.835331 | 1619.661165 | 1262.120233 | 3662.399815 | 2806.658189 | 2303.003776 | 1919.412281 |
| AcOrf-38 | Bacmid_dCC | 45425 | 46075 | - | 650 | 650 | 1 | 3914.211544 | 3144.076008 | 1134.18493 | 1731.169895 | 2316.571366 | 2717.027065 | 1666.643532 | 2842.39917 |
| AcOrf-114 | Bacmid_dCC | 112947 | 114221 | - | 1274 | 1274 | 1 | 3446.790722 | 2835.391216 | 1781.907181 | 2636.678939 | 2448.766638 | 2674.373579 | 2114.362853 | 2677.481102 |
| AcOrf-54 | Bacmid_dCC | 60283 | 61380 | + | 1097 | 1097 | 1 | 3404.042019 | 2809.520728 | 1483.511389 | 2226.713838 | 2441.343217 | 2569.245891 | 1960.900067 | 2656.561235 |
| Ac-pk-2 | Bacmid_dCC | 118025 | 118672 | - | 647 | 647 | 1 | 2605.608198 | 2697.661607 | 1338.623066 | 2021.04644 | 2146.451257 | 2497.25723 | 1643.168951 | 2991.647466 |
| AcOrf-85 | Bacmid_dCC | 86995 | 87156 | + | 161 | 161 | 1 | 981.6547967 | 1772.512214 | 2069.934942 | 1171.648205 | 2002.173208 | 2351.381601 | 2882.068661 | 2764.013432 |
| repE | Bacmid_dCC | 16611 | 17366 | - | 755 | 755 | 1 | 3591.497714 | 2765.346251 | 1384.22727 | 2376.269334 | 2193.25424 | 2307.973542 | 1034.184259 | 1689.168926 |
| Ac-GTA | Bacmid_dCC | 49071 | 50591 | + | 1520 | 1520 | 1 | 2572.943562 | 2338.95626 | 1406.623118 | 1563.041308 | 2518.721626 | 2264.189221 | 1986.514001 | 2296.120087 |
| Ac-env-prot | Bacmid_dCC | 33574 | 35646 | + | 2072 | 2072 | 1 | 2060.980864 | 2116.58357 | 1437.784504 | 1383.389924 | 2342.138661 | 2214.359574 | 1879.245316 | 1900.638823 |
| AcOrf-51 | Bacmid_dCC | 58241 | 59197 | + | 956 | 956 | 1 | 1179.93473 | 1858.458363 | 1617.937663 | 974.5933781 | 2762.154412 | 2171.347989 | 2649.713549 | 1305.636001 |
| Ac-lef5 | Bacmid_dCC | 100979 | 101776 | + | 797 | 797 | 1 | 1185.921686 | 2314.689889 | 1553.215702 | 1002.900415 | 2842.259359 | 2147.712737 | 2822.537791 | 1212.787925 |
| AcOrf-92 | Bacmid_dCC | 93760 | 94539 | - | 779 | 779 | 1 | 1714.566387 | 1805.673878 | 2101.06764 | 2184.261995 | 1935.788528 | 2108.980358 | 2302.072111 | 2282.690221 |
| AcOrf-140 | Bacmid_dCC | 135791 | 135973 | + | 182 | 182 | 1 | 2809.487144 | 2670.643714 | 2463.239471 | 2971.379671 | 2159.351189 | 2099.973299 | 1686.029458 | 2842.664221 |
| AcOrf-18 | Bacmid_dCC | 29459 | 30520 | - | 1061 | 1061 | 1 | 3136.919619 | 2384.266316 | 1016.809916 | 1429.010934 | 1972.729865 | 2012.802331 | 1362.64869 | 2402.296051 |
| Ac-IE-2 | Bacmid_dCC | 144023 | 145249 | - | 1226 | 1226 | 1 | 1622.777774 | 1443.166144 | 1233.574173 | 1244.704896 | 1613.586328 | 1972.39322 | 1632.214617 | 2091.773111 |
| Ac-DNA-pol | Bacmid_dCC | 67390 | 70344 | - | 2954 | 2954 | 1 | 1932.384867 | 1930.252523 | 638.8212751 | 760.0071374 | 1944.784422 | 1922.338039 | 1467.758246 | 1530.74386 |
| AcOrf-69 | Bacmid_dCC | 74337 | 75125 | + | 788 | 788 | 1 | 2929.844346 | 1712.620632 | 1373.56647 | 2609.677313 | 1972.518602 | 1919.388832 | 1915.533067 | 2844.303967 |
| Ac-PNK_PNL | Bacmid_dCC | 87192 | 89276 | - | 2084 | 2084 | 1 | 1717.507976 | 1660.900245 | 1873.623732 | 2302.853001 | 1903.816245 | 1896.530088 | 3256.664941 | 3129.237114 |
| KanR | Bacmid_dCC | 4776 | 5592 | - | 816 | 816 | 1 | 3467.329477 | 2373.607992 | 1938.846792 | 3580.638087 | 1722.955944 | 1875.804371 | 1239.288814 | 1881.474839 |
| Ac-pk-1 | Bacmid_dCC | 21978 | 22796 | + | 818 | 818 | 1 | 2644.335844 | 2106.715835 | 1166.828319 | 1816.477785 | 1819.203399 | 1820.208841 | 1083.248433 | 1489.78183 |
| AcOrf-53 | Bacmid_dCC | 59773 | 60192 | + | 419 | 419 | 1 | 1434.835786 | 1515.962598 | 1028.533805 | 1529.780532 | 1570.279305 | 1815.674411 | 1244.56125 | 2013.322537 |
| T3_promoter | Bacmid_dCC | 5972 | 5999 | + | 27 | 27 | 1 | 1032.983152 | 1295.606897 | 1499.721427 | 1314.391217 | 2575.855258 | 1811.351377 | 1259.592183 | 669.9882002 |
| Ac-odv-e66 | Bacmid_dCC | 51779 | 53893 | + | 2114 | 2114 | 1 | 1357.439544 | 1428.309351 | 1696.535794 | 1423.313222 | 1572.876612 | 1785.646296 | 1687.204801 | 1941.602985 |
| sopA | Bacmid_dCC | 14857 | 16032 | - | 1175 | 1175 | 1 | 2276.079473 | 1643.694251 | 881.5079924 | 1751.543538 | 1563.364066 | 1745.063624 | 701.5241941 | 1140.804589 |
| T7_promoter | Bacmid_dCC | 12850 | 12868 | - | 18 | 18 | 1 | 3787.604891 | 3170.827406 | 1249.767856 | 2805.719713 | 3066.494354 | 1710.720745 | 1294.101558 | 1574.47227 |
| ORF1629 | Bacmid_dCC | 20348 | 21689 | - | 1341 | 1341 | 1 | 1231.72264 | 1405.900526 | 1108.135487 | 976.7344954 | 1471.91729 | 1651.963262 | 880.799682 | 753.6243111 |
| AcOrf-34 | Bacmid_dCC | 43355 | 44002 | - | 647 | 647 | 1 | 1882.360334 | 1437.045476 | 985.4649958 | 1697.425851 | 1542.441921 | 1637.775202 | 1303.782317 | 1951.560683 |
| Ac-lef7 | Bacmid_dCC | 119614 | 120294 | - | 680 | 680 | 1 | 1089.189588 | 1085.722591 | 1410.71485 | 880.7908021 | 1282.516168 | 1627.552487 | 1201.687482 | 1645.806308 |
| AcOrf-30 | Bacmid_dCC | 39376 | 40767 | - | 1391 | 1391 | 1 | 1904.817962 | 1957.602263 | 704.6549787 | 869.2077912 | 1539.6403 | 1625.137684 | 1229.833739 | 1878.327091 |
| Ac-p43 | Bacmid_dCC | 46139 | 47230 | - | 1091 | 1091 | 1 | 2002.529209 | 1527.239835 | 705.7743627 | 971.3502151 | 1620.997822 | 1605.47826 | 1189.956629 | 1595.628176 |
| Ac-IAP2 | Bacmid_dCC | 76077 | 76826 | + | 749 | 749 | 1 | 1617.74264 | 1457.66018 | 1261.447929 | 1832.561303 | 1435.561523 | 1555.005254 | 1773.523854 | 2306.494759 |
| AcOrf-68 | Bacmid_dCC | 73781 | 74359 | + | 578 | 578 | 1 | 1538.75172 | 1312.359417 | 1182.059226 | 1780.095733 | 1447.724878 | 1538.708523 | 1649.106303 | 2210.613316 |
| AcOrf-154 | Bacmid_dCC | 146757 | 147002 | + | 245 | 245 | 1 | 1517.852795 | 961.8941861 | 986.4056964 | 1597.821009 | 1252.631653 | 1508.227269 | 1229.660582 | 1412.717976 |
| AcOrf-113 | Bacmid_dCC | 111850 | 112359 | + | 509 | 509 | 1 | 1248.103416 | 1566.222752 | 1319.569653 | 966.4559508 | 1730.731823 | 1505.307726 | 2108.800778 | 1298.381848 |
| AcOrf-17 | Bacmid_dCC | 28799 | 29293 | + | 494 | 494 | 1 | 2195.611963 | 1617.507098 | 645.9904757 | 1051.062699 | 1318.468423 | 1459.34787 | 800.9807535 | 1530.66535 |
| Ac-sod | Bacmid_dCC | 40881 | 41336 | + | 455 | 455 | 1 | 1954.721965 | 1699.500545 | 745.1520119 | 1356.545065 | 1480.004748 | 1417.233165 | 1043.700017 | 1675.117751 |
| Ac-p47 | Bacmid_dCC | 47238 | 48443 | - | 1205 | 1205 | 1 | 1964.811107 | 1431.647036 | 553.1278433 | 862.4638289 | 1311.899726 | 1375.424489 | 1013.45872 | 1515.730151 |
| AcOrf-11 | Bacmid_dCC | 22960 | 23982 | - | 1022 | 1022 | 1 | 1027.929418 | 888.1359258 | 498.0903145 | 530.2182543 | 1253.001998 | 1368.26151 | 793.4792564 | 1044.906255 |
| AcOrf-106 | Bacmid_dCC | 108934 | 109119 | + | 185 | 185 | 1 | 1005.064688 | 1273.859868 | 1460.925544 | 1060.967846 | 2804.599161 | 1351.170216 | 1534.45469 | 1192.941152 |
| AcOrf-20 | Bacmid_dCC | 31074 | 31283 | - | 209 | 209 | 1 | 800.6855054 | 1030.677962 | 901.0629762 | 464.9955236 | 1912.083944 | 1343.346715 | 1093.732532 | 1139.621077 |
| Ac-p95 | Bacmid_dCC | 82945 | 85488 | + | 2543 | 2543 | 1 | 1497.683398 | 1466.818894 | 1364.585582 | 1790.906241 | 1485.519357 | 1342.665098 | 2405.899166 | 2234.593755 |
| AcOrf-12 | Bacmid_dCC | 24019 | 24672 | + | 653 | 653 | 1 | 669.1452884 | 927.611515 | 800.2232684 | 691.4605132 | 1027.86261 | 1334.241979 | 1308.449636 | 1112.71394 |
| sopB | Bacmid_dCC | 13886 | 14857 | - | 971 | 971 | 1 | 1784.050202 | 1190.758878 | 647.7022581 | 1357.635032 | 1211.944257 | 1320.738182 | 540.5639462 | 947.6434543 |
| Ac-lef8 | Bacmid_dCC | 55584 | 58214 | - | 2630 | 2630 | 1 | 1379.798408 | 1299.28863 | 991.2335861 | 974.6639521 | 1406.996223 | 1319.600471 | 1418.886407 | 1408.65656 |
| AcOrf-96 | Bacmid_dCC | 99407 | 99928 | + | 521 | 521 | 1 | 951.6927313 | 1293.381893 | 944.9848749 | 1036.938814 | 1652.728627 | 1303.755789 | 1674.532641 | 1261.532484 |
| AcOrf-41 | Bacmid_dCC | 48442 | 48987 | + | 545 | 545 | 1 | 1461.339468 | 1185.752445 | 644.5067573 | 990.7745565 | 1158.628472 | 1216.430466 | 1003.557949 | 1502.494639 |
| AcOrf-52 | Bacmid_dCC | 59400 | 59771 | - | 371 | 371 | 1 | 1227.885634 | 1101.697793 | 604.6239252 | 1228.082343 | 833.1607302 | 1069.234371 | 1057.326318 | 1394.514523 |
| AcOrf-21 | Bacmid_dCC | 31366 | 32325 | - | 959 | 959 | 1 | 1027.597421 | 827.4506043 | 454.7419878 | 576.4365106 | 996.8824608 | 1065.278599 | 621.4926104 | 1151.275866 |
| AcOrf-146 | Bacmid_dCC | 139693 | 140298 | - | 605 | 605 | 1 | 1377.880006 | 1028.595784 | 656.5486482 | 1153.32189 | 934.241718 | 993.9977803 | 733.0817947 | 1281.726187 |
| AcOrf-110 | Bacmid_dCC | 110990 | 111160 | - | 170 | 170 | 1 | 601.5607768 | 812.2612899 | 913.0656922 | 1069.474338 | 883.150374 | 969.605737 | 867.8092798 | 1319.482644 |
| Ac-lef9 | Bacmid_dCC | 64245 | 65795 | + | 1550 | 1550 | 1 | 1029.650948 | 871.8620538 | 426.2802654 | 686.3470444 | 910.2146597 | 925.5421229 | 750.0111685 | 1009.909955 |
| AcOrf-112 | Bacmid_dCC | 111582 | 111845 | + | 263 | 263 | 1 | 801.249289 | 679.0463205 | 612.189382 | 910.3091437 | 772.336829 | 881.5702518 | 816.022062 | 1150.953113 |
| AcOrf-152 | Bacmid_dCC | 145275 | 145553 | - | 278 | 278 | 1 | 557.3650101 | 529.8203138 | 470.4932842 | 631.4096822 | 675.0699801 | 820.9722068 | 691.5529762 | 1064.992035 |
| AcOrf-22 | Bacmid_dCC | 32362 | 33510 | + | 1148 | 1148 | 1 | 1074.374463 | 755.3746979 | 491.2912713 | 862.9589561 | 684.6723283 | 766.8264539 | 534.5947062 | 919.1911283 |
| AcOrf-119 | Bacmid_dCC | 115760 | 117352 | + | 1592 | 1592 | 1 | 1078.403266 | 759.8112519 | 840.7671943 | 1136.367926 | 704.5232255 | 765.7283144 | 797.3356381 | 1229.840777 |
| Ac-lef4 | Bacmid_dCC | 91657 | 93051 | + | 1394 | 1394 | 1 | 849.2099657 | 645.8467818 | 507.6429466 | 545.9778901 | 793.5049092 | 740.653002 | 1025.106674 | 1048.094167 |
| Ac-p74 | Bacmid_dCC | 132301 | 134238 | - | 1937 | 1937 | 1 | 948.7232976 | 668.2040734 | 430.206246 | 711.434277 | 712.4018892 | 666.749371 | 544.2041119 | 941.3731985 |
| AcOrf-149 | Bacmid_dCC | 143333 | 143656 | - | 323 | 323 | 1 | 441.3364551 | 473.1065759 | 502.4497925 | 469.0676919 | 512.6646908 | 616.8688899 | 579.8216019 | 696.3313895 |
| AcOrf-57 | Bacmid_dCC | 62134 | 62619 | + | 485 | 485 | 1 | 460.0502285 | 516.2743924 | 320.7062609 | 410.8905771 | 605.4587616 | 582.6202367 | 459.7929493 | 607.9624884 |
| AcOrf-115 | Bacmid_dCC | 114243 | 114857 | - | 614 | 614 | 1 | 741.9308958 | 473.7749891 | 310.9008356 | 509.0754757 | 420.71903 | 498.5641412 | 401.6351674 | 643.2541433 |
| AcOrf-116 | Bacmid_dCC | 114865 | 115035 | - | 170 | 170 | 1 | 309.8949456 | 270.7537633 | 160.6844386 | 244.0842332 | 181.825077 | 266.3752025 | 228.3708631 | 432.7335552 |
| AcOrf-91 | Bacmid_dCC | 93048 | 93722 | - | 674 | 674 | 1 | 183.9139143 | 169.3617012 | 371.4337616 | 435.0001884 | 189.9952585 | 193.4974765 | 332.7031423 | 443.7429266 |
| sopC | Bacmid_dCC_Rep2_Cap2 | 13340 | 13813 | + | 473 | 473 | 1 | 550.3419753 | 467.0931731 | 515.6849424 | 737.5895809 | 144.7022283 | 137.8618384 | 65.66265831 | 104.5351568 |
| Tn7L | Bacmid_dCC_Rep2_Cap2 | 6089 | 6254 | + | 165 | 165 | 1 | 638.5714031 | 1383.633777 | 1462.715314 | 784.2227876 |  |  |  |  |
| Tn7R | Bacmid_dCC_Rep2_Cap2 | 12527 | 12751 | + | 224 | 224 | 1 | 1701.655282 | 1717.83593 | 2305.534757 | 3307.553557 |  |  |  |  |
| SV40_polyA | Bacmid_dCC_Rep2_Cap5 | 6287 | 6508 | - | 221 | 221 | 1 | 4445.099446 | 13512.69551 | 33351.10949 | 9821.425801 |  |  |  |  |
| p40 | Bacmid_dCC_Rep2_Cap2 | 6965 | 6969 | - | 4 | 4 | 1 | 31764.23193 | 54313.20492 | 99865.37858 | 70704.13677 |  |  |  |  |
| p10_promoter | Bacmid_dCC_Rep2_Cap2 | 8631 | 8751 | + | 120 | 120 | 1 | 3847.862241 | 5492.691345 | 6017.632225 | 8003.125905 |  |  |  |  |
| VP2 | Bacmid_dCC_Rep2_Cap2 | 9206 | 11002 | + | 1796 | 1796 | 1 | 5618.139994 | 4248.120516 | 5746.347506 | 7544.435572 |  |  |  |  |
| GmR | Bacmid_dCC_Rep2_Cap2 | 11927 | 12460 | + | 533 | 533 | 1 | 2825.683744 | 1568.238308 | 1705.727117 | 3527.857136 |  |  |  |  |
| polyhedrin_promoter | Bacmid_dCC_Rep2_Cap2 | 8485 | 8611 | - | 126 | 126 | 1 | 5140.320923 | 6210.145841 | 9607.909208 | 11528.36648 |  |  |  |  |
| VP1 | Bacmid_dCC_Rep2_Cap2 | 8795 | 11002 | + | 2207 | 2207 | 1 | 5807.546421 | 4151.083343 | 5516.41982 | 7366.61967 |  |  |  |  |
| Pc_promoter | Bacmid_dCC_Rep2_Cap2 | 11710 | 11738 | + | 28 | 28 | 1 | 4095.040353 | 2104.143532 | 1503.547247 | 4328.8247 |  |  |  |  |
| Hsk_tk_polyA | Bacmid_dCC_Rep2_Cap2 | 11089 | 11370 | + | 281 | 281 | 1 | 3363.628413 | 2692.891878 | 3013.548012 | 4165.753012 |  |  |  |  |
| Rep52 | Bacmid_dCC_Rep2_Cap2 | 6606 | 6969 | - | 363 | 363 | 1 | 8246.791115 | 6223.308814 | 7395.910743 | 10634.53629 |  |  |  |  |
| Rep78 | Bacmid_dCC_Rep2_Cap2 | 6606 | 8480 | - | 1874 | 1874 | 1 | 6328.537657 | 4882.8144 | 6325.685044 | 9342.951957 |  |  |  |  |
| AAP | Bacmid_dCC_Rep2_Cap2 | 9321 | 9935 | + | 614 | 614 | 1 | 5728.51406 | 4539.843883 | 6100.251243 | 8014.937642 |  |  |  |  |
| ITR2 | Bacmid_dCC_AAV2wt | 10884 | 11027 | + | 143 | 143 | 1 |  |  |  |  | 61.75876741 | 50.66717138 | 13.03151219 | 42.16708952 |
| ITR | Bacmid_dCC_AAV2wt | 6349 | 6493 | + | 144 | 144 | 1 |  |  |  |  | 153.3247177 | 176.1036061 | 112.1554683 | 385.2432151 |
| p81_Promoter | Bacmid_dCC_AAV2wt | 10141 | 10161 | + | 20 | 20 | 1 |  |  |  |  | 1103.937968 | 2083.054083 | 1087.045308 | 2110.462831 |
| mTN7 | Bacmid_dCC_AAV2wt | 6089 | 6253 | + | 164 | 164 | 1 |  |  |  |  | 1292.415181 | 1192.841151 | 1443.081054 | 1025.817299 |
| p5_promoter | Bacmid_dCC_AAV2wt | 6603 | 6608 | + | 5 | 5 | 1 |  |  |  |  | 1766.300748 | 2535.891928 | 1055.986871 | 3256.142653 |
| mRNA_Rep78 | Bacmid_dCC_AAV2wt | 6635 | 10799 | + | 4164 | 4164 | 1 |  |  |  |  | 2570.552962 | 2417.745185 | 1130.753868 | 2325.071443 |
| mRNA_Rep68 | Bacmid_dCC_AAV2wt | 6635 | 10799 | + | 4164 | 4164 | 1 |  |  |  |  | 2570.552962 | 2417.745185 | 1130.753868 | 2325.071443 |
| mRNA_Rep40 | Bacmid_dCC_AAV2wt | 7221 | 10799 | + | 3578 | 3578 | 1 |  |  |  |  | 2728.682891 | 2604.640255 | 1185.828153 | 2474.14396 |
| mRNA_Rep52 | Bacmid_dCC_AAV2wt | 7221 | 10799 | + | 3578 | 3578 | 1 |  |  |  |  | 2728.682891 | 2604.640255 | 1185.828153 | 2474.14396 |
| mRNA_VP2/3 | Bacmid_dCC_AAV2wt | 8200 | 10799 | + | 2599 | 2599 | 1 |  |  |  |  | 2781.295041 | 2765.464511 | 1212.701126 | 2664.614479 |
| mRNA_VP1 | Bacmid_dCC_AAV2wt | 8200 | 10799 | + | 2599 | 2599 | 1 |  |  |  |  | 2781.295041 | 2765.464511 | 1212.701126 | 2664.614479 |
| X_Protein | Bacmid_dCC_AAV2wt | 10277 | 10744 | + | 467 | 467 | 1 |  |  |  |  | 2987.96058 | 3052.534333 | 1248.988124 | 2566.901259 |
| PolyA | Bacmid_dCC_AAV2wt | 10772 | 10772 | + | 0 | 0 | 1 |  |  |  |  | 6623.627805 | 9056.756884 | 12423.37495 | 36179.36281 |
| p40_promoter | Bacmid_dCC_AAV2wt | 8171 | 8175 | + | 4 | 4 | 1 |  |  |  |  | 96042.60317 | 76076.75783 | 33387.82019 | 39043.56236 |
