# Supplementary material for: Genetics instability of wtAAV2 genome and AAV promoter activities in the Baculovirus/Sf9 cells system
Source: PLoS One. 2018 Jul 5;13(7):e0199866. doi: 10.1371/journal.pone.0199866 (PMC6033426; doi:10.1371/journal.pone.0199866)
Supplement: S4 Table — (RTF) [file pone.0199866.s004.rtf]

gaattctacccgtaaagcgagtttagttttgaaaaacaaatgacatcatttgtataatgacatcatcccctgattgtgttttacaagtagaattctatccgtaaagcgagttcagttttgaaaacaaatgagtcatacctaaacacgttaataatcttctgatatcagcttatgactcaagttatgagccgtgtgcaaaacatgagataagtttatgacatcatccactgatcgtgcgttacaagtagaattctactcgtaaagccagttcggttatgagccgtgtgcaaaacatgacatcagcttatgactcatacttgattgtgttttacgcgtagaattctactcgtaaagcgagttcggttatgagccgtgtgcaaaacatgacatcagcttatgagtcataattaatcgtgcgttacaagtagaattctactcgtaaagcgagttgaaggatcatatttagttgcgtttatgagataagattgaaagcacgtgtaaaatgtttcccgcgcgttggcacaactatttacaatgcggccaagttataaaagattctaatctgatatgttttaaaacacctttgcggcccgagttgtttgcgtacgtgactagcgaagaagatgtgtggaccgcagaacagatagtaaaacaaaaccctagtattggagcaataatcgatttaaccaacacgtctaaatattatgatggtgtgcattttttgcgggcgggcctgttatacaaaaaaattcaagtacctggccagactttgccgcctgaaagcatagttcaagaatttattgacacggtaaaagaatttacagaaaagtgtcccggcatgttggtgggcgtgcactgcacacacggtattaatcgcaccggttacatggtgtgcagatatttaatgcacaccctgggtattgcgccgcaggaagccatagatagattcgaaaaagccagaggtcacaaaattgaaagacaaaattacgttcaagatttattaatttaattaatattatttgcattctttaacaaatactttatcctattttcaaattgttgcgcttcttccagcgaaccaaaactatgcttcgcttgctccgtttagcttgtagccgatcagtggcgttgttccaatcgacggtaggattaggccggatattctccaccacaatgttggcaacgttgatgttacgtttatgcttttggttttccacgtacgtcttttggccggtaatagccgtaaacgtagtgccgtcgcgcgtcacgcacaacaccggatgtttgcgcttgtccgcggggtattgaaccgcgcgatccgacaaatccaccactttggcaactaaatcggtgacctgcgcgtcttttttctgcattatttcgtctttcttttgcatggtttcctggaagccggtgtacatgcggtttagatcagtcatgacgcgcgtgacctgcaaatctttggcctcgatctgcttgtccttgatggcaacgatgcgttcaataaactcttgttttttaacaagttcctcggttttttgcgccaccaccgcttgcagcgcgtttgtgtgctcggtgaatgtcgcaatcagcttagtcaccaactgtttgctctcctcctcccgttgtttgatcgcgggatcgtacttgccggtgcagagcacttgaggaattacttcttctaaaagccattcttgtaattctatggcgtaaggcaatttggacttcataatcagctgaatcacgccggatttagtaatgagcactgtatgcggctgcaaatacagcgggtcgccccttttcacgacgctgttagaggtagggcccccattttggatggtctgctcaaataacgatttgtatttattgtctacatgaacacgtatagctttatcacaaactgtatattttaaactgttagcgacgtccttggccacgaaccggacctgttggtcgcgctctagcacgtaccgcaggttgaacgtatcttctccaaatttaaattctccaattttaacgcgagccattttgatacacgtgtgtcgattttgcaacaactattgttttttaacgcaaactaaacttattgtggtaagcaataattaaatatgggggaacatgcgccgctacaacactcgtcgttatgaacgcagacggcgccggtctcggcgcaagcggctaaaacgtgttgcgcgttcaacgcggcaaacatcgcaaaagccaatagtacagttttgatttgcatattaacggcgattttttaaattatcttatttaataaatagttatgacgcctacaactccccgcccgcgttgactcgctgcacctcgagcagttcgttgacgccttcctccgtgtggccgaacacgtcgagcgggtggtcgatgaccagcggcgtgccgcacgcgacgcacaagtatctgtacaccgaatgatcgtcgggcgaaggcacgtcggcctccaagtggcaatattggcaaattcgaaaatatatacagttgggttgtttgcgcatatctatcgtggcgttgggcatgtacgtccgaacgttgatttgcatgcaagccgaaattaaatcattgcgattagtgcgattaaaacgttgtacatcctcgcttttaatcatgccgtcgattaaatcgcgcaatcgagtcaagtgatcaaagtgtggaataatgttttctttgtattcccgagtcaagcgcagcgcgtattttaacaaactagccatcttgtaagttagtttcatttaatgcaactttatccaataatatattatgtatcgcacgtcaagaattaacaatgcgcccgttgtcgcatctcaacacgactatgatagagatcaaataaagcgcgaattaaatagcttgcgacgcaacgtgcacgatctgtgcacgcgttccggcacgagctttgattgtaataagtttttacgaagcgatgacatgacccccgtagtgacaacgatcacgcccaaaagaactgccgactacaaaattaccgagtatgtcggtgacgttaaaactattaagccatccaatcgaccgttagtcgaatcaggaccgctggtgcgagaagccgcgaagtatggcgaatgcatcgtataacgtgtggagtccgctcattagagcgtcatgtttagacaagaaagctacatatttaattgatcccgatgattttattgataaattgaccctaactccatacacggtattctacaatggcggggttttggtcaaaatttccggactgcgattgtacatgctgttaacggctccgcccactattaatgaaattaaaaattccaattttaaaaaacgcagcaagagaaacatttgtatgaaagaatgcgtagaaggaaagaaaaatgtcgtcgacatgctgaacaacaagattaatatgcctccgtgtataaaaaaaatattgaacgatttgaaagaaaacaatgtaccgcgcggcggtatgtacaggaagaggtttatactaaactgttacattgcaaacgtggtttcgtgtgccaagtgtgaaaaccgatgtttaatcaaggctctgacgcatttctacaaccacgactccaagtgtgtgggtgaagtcatgcatcttttaatcaaatcccaagatgtgtataaaccaccaaactgccaaaaaatgaaaactgtcgacaagctctgtccgtttgctggcaactgcaagggtctcaatcctatttgtaattattgaataataaaacaattataaatgtcaaatttgttttttattaacgatacaaaccaaacgcaacaagaacatttgtagtattatctataattgaaaacgcgtagttataatcgctgaggtaatatttaaaatcattttcaaatgattcacagttaatttgcgacaatataattttattttcacataaactagacgccttgtcgtcttcttcttcgtattccttctctttttcatttttctcctcataaaaattaacatagttattatcgtatccatatatgtatctatcgtatagagtaaattttttgttgtcataaatatatatgtcttttttaatggggtgtatagtaccgctgcgcatagtttttctgtaatttacaacagtgctattttctggtagttcttcggagtgtgttgctttaattattaaatttatataatcaatgaatttgggatcgtcggttttgtacaatatgttgccggcatagtacgcagcttcttctagttcaattacaccattttttagcagcaccggattaacataactttccaaaatgttgtacgaaccgttaaacaaaaacagttcacctcccttttctatactattgtctgcgagcagttgtttgttgttaaaaataacagccattgtaatgagacgcacaaactaatatcacaaactggaaatgtctatcaatatatagttgctgatgggtaccttctagaattccggagcggccgctgcagatcccccgggctgcagggggggggggggcgctgaggtctgcctcgtgaagaaggtgttgctgactcataccaggcctgaatcgccccatcatccagccagaaagtgagggagccacggttgatgagagctttgttgtaggtggaccagttggtgattttgaacttttgctttgccacggaacggtctgcgttgtcgggaagatgcgtgatctgatccttcaactcagcaaaagttcgatttattcaacaaagccgccgtcccgtcaagtcagcgtaatgctctgccagtgttacaaccaattaaccaattctgattagaaaaactcatcgagcatcaaatgaaactgcaatttattcatatcaggattatcaataccatatttttgaaaaagccgtttctgtaatgaaggagaaaactcaccgaggcagttccataggatggcaagatcctggtatcggtctgcgattccgactcgtccaacatcaatacaacctattaatttcccctcgtcaaaaataaggttatcaagtgagaaatcaccatgagtgacgactgaatccggtgagaatggcaaaagcttatgcatttctttccagacttgttcaacaggccagccattacgctcgtcatcaaaatcactcgcatcaaccaaaccgttattcattcgtgattgcgcctgagcgagacgaaatacgcgatcgctgttaaaaggacaattacaaacaggaatcgaatgcaaccggcgcaggaacactgccagcgcatcaacaatattttcacctgaatcaggatattcttctaatacctggaatgctgttttcccggggatcgcagtggtgagtaaccatgcatcatcaggagtacggataaaatgcttgatggtcggaagaggcataaattccgtcagccagtttagtctgaccatctcatctgtaacatcattggcaacgctacctttgccatgtttcagaaacaactctggcgcatcgggcttcccatacaatcgatagattgtcgcacctgattgcccgacattatcgcgagcccatttatacccatataaatcagcatccatgttggaatttaatcgcggcctcgagcaagacgtttcccgttgaatatggctcataacaccccttgtattactgtttatgtaagcagacagttttattgttcatgatgatatatttttatcttgtgcaatgtaacatcagagattttgagacacaacgtggctttcccccccccccctgcaggaattaagtcttcgaaccaatacgcaaaccgcctctccccgcgcgttggccgattcattaatgcagctggcacgacaggtttcccgactggaaagcgggcagtgagcgcaacgcaattaatgtgagttagctcactcattaggcaccccaggctttacactttatgcttccggctcgtatgttgtgtggaattgtgagcggataacaatttcacacaggaaacagctatgaccatgattacgccaagcgcgcaattaaccctcactaaagggaacaaaagctggagctccaccgcggtggcggccgctctagaactagtggatcccccgggctgcaggaattcacataacaggaagaaaaatgccccgcttacgcagggcatccatttattactcaaccgtaaccgattttgccaggttacgcggctgtcgacctcgagggggggcccggtacccaattcgccctatagtgagtcgtattacgcgcgctcactggccgtcgttttacaacgtcgtgactgggaaaaccctggcgttacccaacttaatcgccttgcagcacatccccctttcgccagctggcgtaatagcgaagaggcccgcaccgatcgcccttcccaacagttgcgcagcctgaatggcgaatgggacgcgccctgtagcggcgcattaagcgcggcgggtgtggtggttacgcgcttcgaagacgctcgacagcgacacacttgcatcggatgcagcccggttaacgtgccggcacggcctgggtaaccaggtattttgtccacataaccgtgcgcaaaatgttgtggataagcaggacacagcagcaatccacagcaggcatacaaccgcacaccgaggttactccgttctacaggttacgacgacatgtcaatacttgcccttgacaggcattgatggaatcgtagtctcacgctgatagtctgatcgacaatacaagtgggaccgtggtcccagaccgataatcagaccgacaacacgagtgggatcgtggtcccagactaataatcagaccgacgatacgagtgggaccgtggtcccagactaataatcagaccgacgatacgagtgggaccgtggttccagactaataatcagaccgacgatacgagtgggaccgtggtcccagactaataatcagaccgacgatacgagtgggaccatggtcccagactaataatcagaccgacgatacgagtgggaccgtggtcccagtctgattatcagaccgacgatacgagtgggaccgtggtcccagactaataatcagaccgacgatacgagtgggaccgtggtcccagactaataatcagaccgacgatacgagtgggaccgtggtcccagtctgattatcagaccgacgatacaagtggaacagtgggcccagagagaatattcaggccagttatgctttctggcctgtaacaaaggacattaagtaaagacagataaacgtagactaaaacgtggtcgcatcagggtgctggcttttcaagttccttaagaatggcctcaattttctctatacactcagttggaacacgagacctgtccaggttaagcaccattttatcgcccttatacaatactgtcgctccaggagcaaactgatgtcgtgagcttaaactagttcttgatgcagatgacgttttaagcacagaagttaaaagagtgataacttcttcagcttcaaatatcaccccagcttttttctgctcatgaaggttagatgcctgctgcttaagtaattcctctttatctgtaaaggctttttgaagtgcatcacctgaccgggcagatagttcaccggggtgagaaaaaagagcaacaactgatttaggcaatttggcggtgttgatacagcgggtaataatcttacgtgaaatattttccgcatcagccagcgcagaaatatttccagcaaattcattctgcaatcggcttgcataacgctgaccacgttcataagcacttgttgggcgataatcgttacccaatctggataatgcagccatctgctcatcatccagctcgccaaccagaacacgataatcactttcggtaagtgcagcagctttacgacggcgactcccatcggcaatttctatgacaccagatactcttcgaccgaacgccggtgtctgttgaccagtcagtagaaaagaagggatgagatcatccagtgcgtcctcagtaagcagctcctggtcacgttcattacctgaccatacccgagaggtcttctcaacactatcaccccggagcacttcaagagtaaacttcacatcccgaccacatacaggcaaagtaatggcattaccgcgagccattactcctacgcgcgcaattaacgaatccaccatcggggcagctggtgtcgataacgaagtatcttcaaccggttgagtattgagcgtatgttttggaataacaggcgcacgcttcattatctaatctcccagcgtggtttaatcagacgatcgaaaatttcattgcagacaggttcccaaatagaaagagcatttctccaggcaccagttgaagagcgttgatcaatggcctgttcaaaaacagttctcatccggatctgacctttaccaacttcatccgtttcacgtacaacattttttagaaccatgcttccccaggcatcccgaatttgctcctccatccacggggactgagagccattactattgctgtatttggtaagcaaaatacgtacatcaggctcgaaccctttaagatcaacgttcttgagcagatcacgaagcatatcgaaaaactgcagtgcggaggtgtagtcaaacaactcagcaggcgtgggaacaatcagcacatcagcagcacatacgacattaatcgtgccgatacccaggttaggcgcgctgtcaataactatgacatcatagtcatgagcaacagtttcaatggccagtcggagcatcaggtgtggatcggtgggcagtttaccttcatcaaatttgcccattaactcagtttcaatacggtgcagagccagacaggaaggaataatgtcaagccccggccagcaagtgggctttattgcataagtgacatcgtccttttccccaagatagaaaggcaggagagtgtcttctgcatgaatatgaagatctggtacccatccgtgatacattgaggctgttccctgggggtcgttaccttccacgagcaaaacacgtagccccttcagagccagatcctgagcaagatgaacagaaactgaggttttgtaaacgccacctttatgggcagcaaccccgatcaccggtggaaatacgtcttcagcacgtcgcaatcgcgtaccaaacacatcacgcatatgattaatttgttcaattgtataaccaacacgttgctcaacccgtcctcgaatttccatatccgggtgcggtagtcgccctgctttctcggcatctctgatagcctgagaagaaaccccaactaaatccgctgcttcacctattctccagcgccgggttattttcctcgcttccgggctgtcatcattaaactgtgcaatggcgatagccttcgtcatttcatgaccagcgtttatgcactggttaagtgtttccatgagtttcattctgaacatcctttaatcattgctttgcgtttttttattaaatcttgcaatttactgcaaagcaacaacaaaatcgcaaagtcatcaaaaaaccgcaaagttgtttaaaataagagcaacactacaaaaggagataagaagagcacatacctcagtcacttattatcactagcgctcgccgcagccgtgtaaccgagcatagcgagcgaactggcgaggaagcaaagaagaactgttctgtcagatagctcttacgctcagcgcaagaagaaatatccaccgtgggaaaaactccaggtagaggtacacacgcggatagccaattcagagtaataaactgtgataatcaaccctcatcaatgatgacgaactaacccccgatatcaggtcacatgacgaagggaaagagaaggaaatcaactgtgacaaactgccctcaaatttggcttccttaaaaattacagttcaaaaagtatgagaaaatccatgcaggctgaaggaaacagcaaaactgtgacaaattaccctcagtaggtcagaacaaatgtgacgaaccaccctcaaatctgtgacagataaccctcagactatcctgtcgtcatggaagtgatatcgcggaaggaaaatacgatatgagtcgtctggcggcctttctttttctcaatgtatgagaggcgcattggagttctgctgttgatctcattaacacagacctgcaggaagcggcggcggaagtcaggcatacgctggtaactttgaggcagctggtaacgctctatgatccagtcgattttcagagagacgatgcctgagccatccggcttacgatactgacacagggattcgtataaacgcatggcatacggattggtgatttcttttgtttcactaagccgaaactgcgtaaaccggttctgtaacccgataaagaagggaatgagatatgggttgatatgtacactgtaaagccctctggatggactgtgcgcacgtttgataaaccaaggaaaagattcatagcctttttcatcgccggcatcctcttcagggcgataaaaaaccacttccttccccgcgaaactcttcaatgcctgccgtatatccttactggcttccgcagaggtcaatccgaatatttcagcatatttagcaacatggatctcgcagataccgtcatgttcctgtagggtgccatcagattttctgatctggtcaacgaacagatacagcatacgtttttgatcccgggagagactatatgccgcctcagtgaggtcgtttgactggacgattcgcgggctatttttacgtttcttgtgattgataaccgctgtttccgccatgacagatccatgtgaagtgtgacaagtttttagattgtcacactaaataaaaaagagtcaataagcagggataactttgtgaaaaaacagcttcttctgagggcaatttgtcacagggttaagggcaatttgtcacagacaggactgtcatttgagggtgatttgtcacactgaaagggcaatttgtcacaacaccttctctagaaccagcatggataaaggcctacaaggcgctctaaaaaagaagatctaaaaactataaaaaaaataattataaaaatatccccgtggataagtggataaccccaagggaagttttttcaggcatcgtgtgtaagcagaatatataagtgctgttccctggtgcttcctcgctcactcgagggcttcgccctgtcgctcgactgcggcgagcactactggctgtaaaaggacagaccacatcatggttctgtgttcattaggttgttctgtccattgctgacataatccgctccacttcaacgtaacaccgcacgaagatttctattgttcctgaaggcatattcaaatcgttttcgttaccgcttgcaggcatcatgacagaacactacttcctataaacgctacacaggctcctgagattaataatgcggatctctacgataatgggagattttcccgactgtttcgttcgcttctcagtggataacagccagcttctctgtttaacagacaaaaacagcatatccactcagttccacatttccatataaaggccaaggcatttattctcaggataattgtttcagcatcgcaaccgcatcagactccggcatcgcaaactgcacccggtgccgggcagccacatccagcgcaaaaaccttcgtgtagacttccgttgaactgatggacttatgtcccatcaggctttgcagaactttcagcggtataccggcatacagcatgtgcatcgcataggaatggcggaacgtatgtggtgtgaccggaacagagaacgtcacaccgtcagcagcagcggcggcaaccgcctccccaatccaggtcctgaccgttctgtccgtcacttcccagatccgcgctttctctgtccttcctgtgcgacggttacgccgctccatgggtattttcagtgttgccaccatcgtctgcagctggctgacgtaccaggagtcagagagcggaaccagccggtgagtctgctgaccggcgggcattctccccgccgtcctggcagctttttcggtccgttgtttcagggtcgcaagctgcacaaacggatacggaggcgcaagcgaaaaatccccccgcgtcagcgccagtgcttcattaatgcgtgctccggtgttccacagtgtggccagcagcatcttgcggtgcagatccgggcagcttgattcgccggtcacgcaagtttctatgcgattccagttccagtggcagacagactctgagtgcgttccgacgtaatggagcagggcactcacttccggagccagcagatattttggcagttcatcatggaccatcgacatctggcgaagtgccagagctgccggataatcaatggcaacaggcagcgatgcaggctgcccggcagaatacactgccggtaccatgactgcagactggctgtgtataacggagcctgacatttatattccccagaacatcaggttaatggcgtttttgatgtcattttcgcggtggctgagatcagccacttcttccccgataacggagaccggcacactggccatatcggtggtcatcatgcgccagctttcatccccgatatgcaccaccgggtaaagttcacgggagactttatctgacagcagacgtgcactggccagggggatcaccatccgtcgcccgggcgtgtcaataatatcactctgtacatccacaaacagacgataacggctctctcttttataggtgtaaaccttaaactgcatttcaccagtccctgttctcgtcagcaaaagagccgttcatttcaataaaccgggcgacctcagccatcccttcctgattttccgctttccagcgttcggcacgcagacgacgggcttcattctgcatggttgtgcttaccagaccggagatattgacatcatatgccttgagcaactgatagctgtcgctgtcaactgtcactgtaatacgctgcttcatagcacacctctttttgacatacttcgggtatacatatcagtatatattcttataccgcaaaaatcagcgcgcaaatacgcatactgttatctggcttttagtaagccttatgtattttacctttcgttatgttaaccaataaaaattaaaatctgacttataaaaacaaagcgtaattaccgcattcccgtttcgtatggatctgatcctttcctgggacccggcaagaaccaaaaactcactctcttcaaggaaatccgtaatgttaaacccgacacgatgaagcttgtcgttggatggaaaggaaaagagttctacagggaaacttggacccgcttcatggaagacagcttccccattgttaacgaccaagaagtgatggatgttttccttgttgtcaacatgcgtcccactagacccaaccgttgttacaaattcctggcccaacacgctctgcgttgcgaccccgactatgtacctcatgacgtgattaggatcgtcgagccttcatgggtgggcagcaacaacgagtaccgcatcagcctggctaagaagggcggcggctgcccaataatgaaccttcactctgagtacaccaactcgttcgaacagttcatcgatcgtgtcatctgggagaacttctacaagcccatcgtttacatcggtaccgactctgctgaagaggaggaaattctccttgaagtttccctggtgttcaaagtaaaggagtttgcaccagacgcacctctgttcactggtccggcgtattaaaacacgatacattgttattagtacatttattaagcgctagattctgtgcgttgttgatttacagacaattgttgtacgtattttaataattcattaaatttataatctttagggtggtatgttagagcgaaaatcaaatgattttcagcgtctttatatctgaatttaaatattaaatcctcaatagatttgtaaaataggtttcgattagtttcaaacaagggttgtttttccgaaccgatggctggactatctaatggattttcgctcaacgccacaaaacttgccaaatcttgtagcagcaatctagctttgtcgatattcgtttgtgttttgttttgtaataaaggttcgacgtcgttcaaaatattatgcgcttttgtatttctttcatcactgtcgttagtgtacaattgactcgacgtaaacacgttaaataaagcttggacatatttaacatcgggcgtgttagctttattaggccgattatcgtcgtcgtcccaaccctcgtcgttagaagttgcttccgaagacgattttgccatagccacacgacgcctattaattgtgtcggctaacacgtccgcgatcaaatttgtagttgagctttttggaattatttctgattgcgggcgtttttgggcgggtttcaatctaactgtgcccgattttaattcagacaacacgttagaaagcgatggtgcaggcggtggtaacatttcagacggcaaatctactaatggcggcggtggtggagctgatgataaatctaccatcggtggaggcgcaggcggggctggcggcggaggcggaggcggaggtggtggcggtgatgcagacggcggtttaggctcaaatgtctctttaggcaacacagtcggcacctcaactattgtactggtttcgggcgccgtttttggtttgaccggtctgagacgagtgcgatttttttcgtttctaatagcttccaacaattgttgtctgtcgtctaaaggtgcagcgggttgaggttccgtcggcattggtggagcgggcggcaattcagacatcgatggtggtggtggtggtggaggcgctggaatgttaggcacgggagaaggtggtggcggcggtgccgccggtataatttgttctggtttagtttgttcgcgcacgattgtgggcaccggcgcaggcgccgctggctgcacaacggaaggtcgtctgcttcgaggcagcgcttggggtggtggcaattcaatattataattggaatacaaatcgtaaaaatctgctataagcattgtaatttcgctatcgtttaccgtgccgatatttaacaaccgctcaatgtaagcaattgtattgtaaagagattgtctcaagctcggatcccgcacgccgataacaagccttttcatttttactacagcattgtagtggcgagacacttcgctgtcgtcgacgtacatgtatgctttgttgtcaaaaacgtcgttggcaagctttaaaatatttaaaagaacatctctgttcagcaccactgtgttgtcgtaaatgttgtttttgataatttgcgcttccgcagtatcgacacgttcaaaaaattgatgcgcatcaattttgttgttcctattattgaataaataagattgtacagattcatatctacgattcgtcatggccaccacaaatgctacgctgcaaacgctggtacaattttacgaaaactgcaaaaacgtcaaaactcggtataaaataatcaacgggcgctttggcaaaatatctattttatcgcacaagcccactagcaaattgtatttgcagaaaacaatttcggcgcacaattttaacgctgacgaaataaaagttcaccagttaatgagcgaccacccaaattttataaaaatctattttaatcacggttccatcaacaaccaagtgatcgtgatggactacattgactgtcccgatttatttgaaacactacaaattaaaggcgagctttcgtaccaacttgttagcaatattattagacagctgtgtgaagcgctcaacgatttgcacaagcacaatttcatacacaacgacataaaactcgaaaatgtcttatatttcgaagcacttgatcgcgtgtatgtttgcgattacggattgtgcaaacacgaaaactcacttagcgtgcacgacggcacgttggagtattttagtccggaaaaaattcgacacacaactatgcacgtttcgtttgactggtacgccgtcggcgtgttaacatacaagttgctaaccggcggccgacacccatttgaaaaaagcgaagacgaaatgttggacttgaatagcatgaagcgtcgtcagcaatacaatgacattggcgttttaaaacacgttcgtaacgttaacgctcgtgactttgtgtactgcctaacaagatacaacatagattgtagactcacaaattacaaacaaattataaaacatgagtttttgtcgtaaaaatgccacttgttttacgagtagaattctacgtgtaacacacgatctaaaagatgatgtcattttttatcaatgactcatttgttttaaaacagacttgttttacgagtagaattctacgtgtaaagcatgatcgtgagtggtgttaataaaatcataaaaattattgtaaatgtttattatttaaaaacgattcaaatatataataaaaacaatctacatctatttcttcacaatccataacacacaacaggtccatcaatgagtttttgtctttatccgacatactatgtgcatgtaacaaatcaaatacatcttttaaatttttatacacatctttacattgtctaccaaaatctttaataaccctataacaaggaaaagacttttcttcttgcgtggttttgccgcgcagatattgaaataaaatgtgcatgcacgacaacttgtgtttactaaaatgctccttgcctataccgcaaaaccggccatacatttcggcgattacacgcggacaattgtacgattcgtctacgtgtaaacgatcatcataatcactcttgcgcaaacgaataaattttttcaccgcttccgacaaacgaggcaccaattcggcgggcacgcttcgatacattattctgtgcacataagttaccacacaaaatttattgtaccaccatccgacaacgtcgttattagggttgaacacgttggcgatgcgcagcagtttcccgtttctcatgaaatattcaaagcggcccaaaataatttgcaagcaatccaacatgtcttgagaaatttctcgttcaaaattgttcaaagagaatatctgccatccgttttgaacgcgcacgctgacgggaaccaccgcatcgatttgctccaacacttcacggacgttatcgtcgatgcccatcgtttcgctggtgctgaaccaatgggaaaggctcttgatggaatcgcccgcgtctatcatcttgaccgcttcgtcaaaggtgcaactgccgctcttcaaacgccgcatagcggtcacgtcccgctctatgcacgacataccgtttacgtacgattctgataggtattcctgaactatacggtaatggtgatacgactcgccatacacgtcgtgcacctcattgtatttagcataataattgtaaattattaactttgcagcgagagacatgttgtcagtaaagcggtgctaggctcaataatactgatgtacaggcacgcgtgctatttatatataatttcgcaaggaggggagctgttatcggttgctattattaaagaatggccgtctgtttttatcacaagcttggcagcctcaaccatgaagcgtcgtcattgtaaattaaattctctgcctcaagaattatttgacaagattgtcgagtatttatctttatctgattactgcaatttggtgcttgtctgtaaaagaccttctagtaaatataacgtgatatttgatagtactaatcaccaacatttgaaaggcgtgtacaaaaagacagacgtgcaaataacaagctacaacgaatacatcaactgtatttgcaacgaactgagacaagacgaattctatgccaaatcatcatggattgcgagtatttgcggtcaccagagagcgacaatttttagtgtaacaaataaacaagtagaaatgaaatatcatttgtataatatagcaattgtggaaagtgaagattgcaacggattttacccatttgagccaacgcgcgattgtttaatatgcaaacaaaaaaaccaatgtcctcgtaattcatttattgtttcgttgtgtaaatatttagaaaaacaaaatgtacaatcaaactttatatattatttatacgaaataaatacataataataactattatacatgtttttattttacaatacttcctgtataacctctctaactacattaggagtacaatccacgtcaattacacgtttagctatttttctaattttgtaatgtttatcgtagagtttttcgttaatacattgaatagccaacaagggatttgggtgcacaccgtcatagagtacttccatgtcgtcttcaaagcgcatttttcgcttgcgaaaatgccgctcttggcccaaaacaaaagcgagtttgatgcggtcgtcgatgcgttccgaaaatacggccaaatgctggtgtttggtgatgtcgcgcggaaacgtcaccgtgccatttttgctttccgccacgacggcggttttcaatttttcggccgactgcagcatgttaagtttggcgtcgagttcgtgcaaacgcaattcaaactgctcaaacctgttgcccacctcgttcttgaacgtctcgtgggtgaccataaatttttcgctgtttgcattcagtttctttacatgttttaaaacagattcaatcttgtcgcgcaaatcatcacgctcgccttcagtttgaatgtgcagcaacgcgttgcttttgttggcaaaatttaaccgcatcaaaatttccaacaacccgtgcttggtcgcgaacaatgcgcccaacgagttgagatcgcgtttggatctctgtttgtgaaaaacaatttcgtttaaatggtaaacttgatcgccgtcccaattgcaatcaagtatgtcgtcgtgcgcaatttcaagacctttgcaaaaatctatcacattgtagcattttgcgttcgtgtcgctgtgcacgtatctgtacttgaaactgtgcgtgttgcatttgaatgagtcccatttaacgatgtgcgaccattgttgggcgtttatgtggtactttttgtagtcgtctgcattgaaccgatcttcggcggcgatggcgtcgttgtcgttgtcaccggaccacatccaccagttccataaccaggatagcattgctttagcttgtctagcaattcctttgttatacaacgagaaaatttcgttcccttataattatagctgtacggtgcgcgtatttgtttgttaacgttacaaaaaatatccctgtccacgtccggccaatactgcaacgtgagcgcgtccaagtttgaatcttgcatatgcggaacgtacaaacgtacggcctctctcacacaatgcgcaaaactgcccggctgaatgtaatcactgtccaactttgcaggtttctcgaaagccttgtaccgatgcacgcgaacattttgagcggacgtgattttaaacttgtcggtgaattttaaccacaaatgaaatccacggttgccggtatacatgactcttgacacgttctcttccgtgtaaaacaacagaaacgccgtggcgccaatgtaaattttcagcattaaatcgtgttcgtcaacataatttttgtaatcggcgtctacgacccattccctgccgccgccgtcgtccaacggtttgacgtgcacgtcggacactttgttttgcacaatataactatacaattgtgcggaggtatcaaaatatctgtcggcgtgaatccagcgcgcgttgaccgtcatgaacgcgtacttgcggctgtcgttgtacgcaatggcgtcccacatcatgtcgacgcgcttctgcgtataattgcacactaacatgttgccctttgaacttgacctcgattgtgttaatttttggctataaaaaggtcaccctttaaaatttgttacataatcaaattaccagtacagttattcggtttgaagcaaaatgactattctctgctggcttgcactgctgtctacgcttactgctgtaaatgcggccaatatattggccgtgtttcctacgccagcttacagccaccatatagtgtacaaagtgtatattgaagcccttgccgaaaaatgtcacaacgttacggtcgtcaagcccaaactgtttgcgtattcaactaaaacttattgcggtaatatcacggaaattaatgccgacatgtctgttgagcaatacaaaaaactagtggcgaattcggcaatgtttagaaagcgcggagtggtgtccgatacagacacggtaaccgccgctaactacctaggcttgattgaaatgttcaaagaccagtttgacaatatcaacgtgcgcaatctcattgccaacaaccagacgtttgatttagtcgtcgtggaagcgtttgccgattatgcgttggtgtttggtcacttgtacgatccggcgcccgtaattcaaatcgcgcctggctacggtttggcggaaaactttgacacggtcggcgccgtggcgcggcaccccgtccaccatcctaacatttggcgcagcaatttcgacgacacggaggcaaacgtgatgacggaaatgcgtttgtataaagaatttaaaattttggccaacatgtccaacgcgttgctcaaacaacagtttggacccaacacaccgacaattgaaaaactacgcaacaaggtgcaattgcttttgctaaacctgcatcccatatttgacaacaaccgacccgtgccgcccagcgtgcagtatcttggcggaggaatccatcttgtaaagagcgcgccgttgaccaaattaagtccggtcatcaacgcgcaaatgaacaagtcaaaaagcggaacgatttacgtaagttttgggtcgagcattgacaccaaatcgtttgcaaacgagtttctttacatgttaatcaatacgttcaaaacgttggataattacaccatattatggaaaattgacgacgaagtagtaaaaaacataacgttgcccgccaacgtaatcacgcaaaattggtttaatcaacgcgccgtgctgcgtcataaaaaaatggcggcgtttattacgcaaggcggactacaatcgagcgacgaggccttggaagccgggatacccatggtgtgtctgcccatgatgggcgaccagttttaccatgcgcacaaattacagcaactcggcgtagcccgcgccttggacactgttaccgtttccagcgatcaactactagtggcgataaacgacgtgttgtttaacgcgcctacctacaaaaaacacatggccgagttatatgcgctcatcaatcatgataaagcaacgtttccgcctctagataaagccatcaaattcacagaacgcgtaattcgatatagacatgacatcagtcgtcaattgtattcattaaaaacaacagctgccaatgtaccgtattcaaattactacatgtataaatctgtgttttctattgtaatgaatcacttaacacacttttaattacgtcaataaatgttattcaccattatttacctggtttttttgagaggggctttgtgcgactgcgcacttccagcctttataaacgctcaccaaccaaagcaggtcattattgtgccaggacgttcaaaggcgaaacatcgaaatggagtctgttcaaacgcgcttatgtgccagtagcaatcaatttgctccgttcaaaaagcgccagcttgccgtgccggtcggttctgtgaacagtttgacacacaccatcacctccaccaccgtcaccagcgtgattccaaaaaattatcaagaaaaacgtcagaaaatatgccacataatatcttcgttgcgtaacacgcacttgaatttcaataagatacagtctgtacataaaaagaaactgcggcatttgcaaaatttgctaagaaaaaagaacgaaattattgccgagttggttagaaaacttgaaagtgcacagaagaagacaacgcacagaaatattagtaaaccagctcattggaaatactttggagtagtcagatgtgacaacacaattcgcacaattattggcaacgaaaagtttgtaaggagacgtttggccgagctgtgcacattgtacaacgccgagtacgtgttttgccaagcacgcgccgatggagacaaagatcgacaggcactagcgagtctgctgacggcggcgtttggttcgcgagtcatagtttatgaaaatagtcgccggttcgagtttataaatccggacgagattgctagtggtaaacgtttaataattaaacatttgcaagatgaatctcaaagtgatattaacgcctattaatttgaaaggtgaggaagagcccaattgcgttgagcgcattaccataatgccatgtattttaatagatactgagatctgtttaaatgtcagatgccgttctccttttgccaaattcaaagtattgattattgtagatggctttgatagcgcttatattcaggctaccttttgtagcattagcgatagtgtaacaattgttaacaaatctaacgaaaagcatgtaacgtttgacgggtttgtaaggccggacgatgaaggtacaacaatgccttatgtcattggaccattatattctgtcgacgctgctgtcgccgaccgtaaagtgaaggacgtggtggattcaattcaaaaccaacagacaatgttaaaagtatttattaacgaggctaatgtgtataacaaatggaatatgcttaaaggtttaatttataataataacaatgaatctgttttagtaaaataatgtagtaaaatttataaaggtagataaaaattataatattaataaaaaaaataatgttactaaatgggttcctgcgttaaattattttacgggtagacagctattaactattttatttatttttaaatttaaataaatgtattgttagaaaattgtgttgttttattagtataacgaaaaaatacatgacataaaccgcttccaattttggtcacacaaactcttgtgtggatagtttacgtaatgagttaaataggcgggcagttgtccgctaaacgtgtcggtggtcaagtagatgtgcattaatttacgacaacccaaagcggggccgcttatgtcaagtatttttttcacaaaattggtaatggtttcgttttgttccttgtacaaacacatgtcggtgtgatcgttgacgcacgagttgtacgattccgccggcaggttggcaaacaagcgcttgagatgcttgagtctgcgttcaattttataatcaaacttgttggtgaaaatgtctttcagcaagcacattaactggtcgttcaaaacgcgctgcaacgacgacaccaacacatgatattcgtttccaaaaagcgaaaaatttttgatgcagcggtccgcgttgaagggtcgtttcataatgcgcacgttgacaaaaaacacgttgaaagacagcggggctgtggttattttaacgccgttgtcggtatactcgtcgacgccgtctgcgcttgttatgtcaatttgtagcgcaaatctaaccaaatcaaactcatcgttgtactgtgtctttatgcattttatatggcggtttaagtgcaagttgatttggccgtttaatctataggctccgttttgataacatttcagcactaccaacggatccgacatgtaaacttgacgcgttagcacgtccaattcagcgtaatgttggtcgacgcatttttgtaaattagtttgcaggttgcaaaacatttttgcgcaaaagccgtaatagtcaaaatctatgcattttaatgcgcttctgtcgtcgtcaatatggcatgtcacggctgcgcctccagttaacacgaataaaccgccgttttcgcaaactacggcttcgaaacaatctttgataaatgccaactttgctttagccacaattttatcgcgcaggcgatcttcaatatcctttgtcgtaatataaggtaggacgccaagatttagttgattcaacaaacgttccataatgaatagcggcgacgcaacacgactacactgttcaaatgcgcacgcaaaacaaacccttgcaactttatttgccaatcgtaatcacagtagtttttacgagtacgccatcgcgtttgtaagcacattgctttttaaaaataatttaaatttaatgaccgcgtgcaatttgatcaactcgttgatcaactttgaactcaacatgtttggtaaaagtttattgctaaatggatttgttaatttctgcattgctaacagcgacggggttacgattcaacataaaatgttaaccaacgtgttaagttttttgttggaaaaatattattaaaaataaataaataaacttgttcagttctaattattgttttattttttataaaataatacaattttatttatacattaatactttggtatttattaatacaattatttacaatactttatttacactataatactttatttacattagtactaaattaatactaaattacgctaatactaaattaatactttatataatcaaaaataatactttatataatactttctaatcatcataaacgggtaatagttttttctcttgaaatttacgctgcaactcttcgctaaaacacatgggcggtggagtgggagcgggtggagtaggagtccttacgggtttgatgggcgacagttctctggacttgcggaacagcttgggcgaaagcgtcggcgtgcgccgactaatgatttcttcatcgcacgaggcgtcgcacattgtgcacgcgtccggtgaggtacacaaaactttcttgggcacgctgtacaccggcttgggcacgctatatgtgttgccaaaactagaactcgttgtggttgccgaacggagacgatgggtgtgaagacggcgatggctgtgaagacaagtccgaaggcgcgataaaagatgaaagtgtttctgaaaccgaagtggtggtagaagtggtagaaggcgggtgcgttacggcaaccacgctgctgctatttctgccttcggagaccacttccagcaatctagagttactctctcgttcttcgcggcgatagtcaatgtcgcaataatgttcataagatgccttttcggcttcggcgcgccttttcatgtatatgttgtgacgcatctcctttaactgcacgtacaaattccagcattgcacagccagtatcgtaagcacgcccattatgattacgggataattttgattaaacacggtcggctcgtgatcgcttacaatcgctcggcacatgatgcattttttgtaaatgttcacatacacacagttttggctcaaggtttcggtatttgcgtagtcaatttccagatacacgatagagttccagcacattgattccaaatcgtagtgacgatataaaacatctagcgccggtagatgaccatttttgaacacgtagatttgaaacgcggcaaacagcatccaacacagcccagtgatcacgtttaccataatacacgtgatagcgacgtaaaagttttctttcgcattgaaatttacatttgtgtttgaagagctgctgcgatttttcgtccacacgataatcttccatataaaataaaacatgtaaaataatatccacatgccgaacgccagcattatcggtatagatagattgataaccgattgctttccttcaatttccagcaaaaacgcgtatctgctgtctatcactcccattatagataacacaaacactatcagatatgctaataataatgaggcattaagcccgaattgtaaaactgcagtgattttatttaacattttgaatatttaattcaacaactaagtaatggcaatatgtatcgagtactgatcgtgtttttcctgttcgtgtttctttatatagtgtaccagcccttttatcaggcatacttgcatatcggacatgcccaacaagattacaatgacacgttggacgataggatggattacattgaatccgtaatgcgtagaaggcactacgtgccgattgaagcgttgcccgcaatcaggtttgatactaatctcggcacgttggccggtgacacgattaaatgcatgtcggtgcctttgtttgttagtgacattgacctgccgatgtttgattgtagtcagatatgcgataacccgtctgcggcgtatttctttgtcaacgaaacggatgtgtttgtggtcaacggccacagactgacggtgggcggatactgctccactaatagtttgccccgcaactgtaatcgcgagacgagcgtcattttaatgagtctcaatcagtggacgtgcatagccgaggacccgcgttactatgcgggcacagataacatgacgcaactcgcaggcagacaacactttgaccgcattatgcccggacagagtgataggaacgtcctgtttgaccgattactaggccgagaggtgaacgtgaccactaacacgtttcgccgcagctgggacgagttgctggaggacggcactaggcggttcgaaatgcgctgcaacgcccgagataacaacaataatctcatgtttgttaatccgcttaatcccctcgagtgtctcccgaacgtgtgcactaacgttagcaacgtgcacaccagtgttagacccgtatttgaaacgggagagtgtgactgcggcgacgaagcggtcacgcgtgttacgcacattgtgccgggggacaggacctctatgtgtgccagcattatagatggcctggataaaagtacggcatcatatagatatcgcgtagagtgcgttaatctgtacacctctattctaaattattctaataacaaattgttatgtcccagtgacacttttgatagtaacacggacgcagcttttgcctttgaagtgcccggctcctaccctttatcgcgcaacggcatcaacgagccaacttatcgcttttatcttgataccagatctcgagttaattacaatgacgtcagagggcagttatcttaattgtgataacacaaacaataagtcatttaaatgttacgtcagtagttagtatataagccgtacatgttggcttgcaaattcagtcaatatcaggcttttatcatggacggtgtaaagctgctagggacgtgcgcgctaataattttgttatcgacgacgagtacagttgtcgggcgtgaccgtatcacgtttacgccgatagaagatagcgcaggcctcatgtttgaacgcatgtacggcttgcgacatcatacagacgacagatttgtgtttgtgaaaaaattcaattttgtttcggtgctgcaagagctcaataatatcaaatctaaaattgaattatatgaagcgcaagtttcaacttgcacaaacgtcagacaaataaaacagaacagatcgagtatcatcaaagctcgcattgaaaatcagctgcagtttttgacgcaactaaacaaaaatctcatcacatactctgtggaaagcagcattttaagcaacgacgtgctggacaacatcgatctggaatatgacgacagcggtgagtttgacgtttacgacgaatacgaacagccttcgcattggagcaacatgactgtatccgacgcgcaagctttgctccgaaacccgcccaaagacagagtaatgtttttggacacggttaccaccagcgacgtgagcagcaaatacgaagaatacataaactgcattgtgagcaaccgtaccgttgaaaacgagtgcatgtttttagccaacatgatgaacgtgctcaacgacaaattggacgacgcagcagctttggccaagatgctggagcgaatagtaaaacaaacgcgaaagaacaaactcaacatctccaacacggttatagacgacgacacgctgctaacggaaatgaaaaaattaacacaaactttatacaaccaaaaccgcgtgtgggtagtggattttaacaaggacatgaatagttatttcgatttgtcgcaagcgtataaattgcatttatatgttgatttaaacacggtcattatgtttattaccatgccattgttaaaatccaccgccgtttcgtttaatttgtatcgcgtcatgacggtgcctttttgcaggggcaaaatgtgtctgcttatcatttcgggcaatgaatactttgggattacagacagcaaaaactattatgtgcccgtatctgataactttagacaagattgccaagagtttacgggctacaatgagtttttgtgtcccgaaactgagccgattgccactatgaactcgaaagtgtgcgagattgaaatgtttatgggtcgatatagcgacgacgtggacaacatgtgcgacattagggtggccaattataatcccaaaaaagcttacgtgaacactttaatagactaccgaaaatggttgtacatttttccaaacacgaccgtgtccgtccactattattgtcacgacgcgcttgtagaagttgatacaaaagtttcgcccggcgttggtgttatgttttcgactatggcgcaaacgtgttcgattagaataacgtatgatgtgaccataactgtagattcgcgattttatgtcagccattcaactacatactggcctaaaaagaaatttaattttaacaactacatcgaccaaatgttgcttgaaaaagcgaccaccagttttataccgactgttgacaattttacccggcccgttttattgcaacttcctcataaatttcacattaaagattacacatcgacgccccatcattttttccatcagtctaaaatttacaccaacagcgcggcgcccgacgaagactcgcaagacgacagtaataccaccgtggtaattatcgctattgtcgctgcaatgatcctattctgtggattattgttatttttgttttgctgtataaaaaaacggtgtcatcaatcaaataacgtggttgtgcaatacaaaaataacaatgaatttgtcacaatttgcaataatttagaagacaatcgagcatacattaatttacctaatgaatacgatagcgatgatatgccaaaaccattgtaccctttacttggctttaatgatgatttgttaaaagatgataaacctgtgttgtaccctatgattatagaaagaataaaataaaacatgtataattgaaataaatatattatttaataaaatgttttttatttatatactattttctattacatattccaatgcacacaaatgtttaatggctatcagttttaattttactaattcgtctaaacaaaaattattcacttgctgtttttcatccatttgacatatggcgtttataaataattcgctgtgttttatgaacgaatcgtaaaccgctgcctgggccttcagcacggtcggcgcattgtatttttgggtaaagtacgcaatatttttagtcaaacacagagattttaaatctttttcatttatatccaagtcggaacaatcgtatacaaaatctagcttttcactttcgggcgcgcccagatactggtttacgagttcgagctgctccacttggcctttgatatcggccgctatgcacaacattttgtcgattgcagtttcattgtttttaacataataatttttaacttttttattttgcaatttaatcaaactatttaaattcgcttgacctttcttacaaagcgcagttaatatgcaagacattttgacttataataaaaaacaaaacttttatatattcatttattgttcaataataacaaatattccaggcttaaaagctaacgaatagggcttttcggtaattttcttattattcatgtccgtcatctgcatctctttgccgtacttgacgccgtcaatggtgcccatcatgtacattttaatctcctccgaaggtccgtctattttgtccatttcgaacaatctatcaaaatcttcaacgctcattctctgtatatcaagaggaacgtttctgatctttccggtggcgtaaattgatccgttgttgtcacggttgattatgtaaaaccgacgaatcaacatgtcgcgctcgctagttttgttcttatccggcaaatgaatgcacacgtttggttccatcttcaaaggaaaatcgctttgcaagtgtttttgcaaaatgttgccaaatatattgttgtgtttgtgaatgtctccgtattgaatgctaaaaaactggccaaagttgcttttggcacgttttatggttccaaagtcggaaaaccaaaatccgcagggcttgccctgcactcttggaccgatggtgtacgtagtcttgccgttggccggctccaacaccacgatatttttatcgggctcgggatacaacttgtcttcccattcgtgcaaactgttcaaattagacagtcgacaaaattcgtttttcaaaaatctgccttcgaaacaactacaattcagtattgaaaagttgcctcgtttcacattaatcgccatctgctcctgccacaacatcttcgtcaactcgtgtggctccaattgaatggacgacggcgtaaaatagcacattacgcccgtttcgtcgtgtttcacgttaaaagcgccgctgttgtacggcaccagctgctggtcctcaccaccttccgatctttcccgcttcggctggttgtcgtcgctgctcgaatatccatcgccaatcttgcgtttagttgccatgctaccgacgtgcgctgtctgctgtggttcaagtctaattgaagtgtttcacagaatataagatatataataaatatggacgactctgttgccagcatgtgcgtagacaacgcgtttgcgtacactactgacgatttattgaaaaatattccttttagtcattccaaatgcgcccctttcaagctacaaaattacaccgttttgaagcggttgagcaacgggtttatcgacaagtatgtggacgtgtgctctatcagcgagttgcaaaagtttaattttaagatagatcggctaaccaactacatatcaaacattttcgagtacgagtttgtagttttagaacacgatttgtccacagtgcacgtcattaacgccgaaacaaaaaccaaactgggccatataaacgtgtcgctaaaccaaaacgacgcaaacgtgctcattttgaccgtaactttaacgagctaaaatgaacgaggacacgcccccgttttattttatcagcgtgtgtgacaactttcgcgacaacaccgccgaacacgtattcgacatgttaatagaaagacatagttcgtttgaaaattatcccattgaaaacacggcgtttattaacagcttgatcgttaacgggtttaaatacaatcaagttgacgatcacgttgtgtgcgagtattgcgaagcagaaataaaaaattggtccgaagacgagtgtattgaatatgcacacgtaaccttgtcgccgtattgcgcgtatgctaacaagatcgccgagcgtgaatcgtttggcgacaacattaccatcaacgctgtactagtgaaagaaggcaaacccaagtgtgtgtacagatgcatgtccaatttacagtcgcgtatggatacgtttgttaacttttggcctgccgcattgcgtgacatgattacaaacattgcggaagcgggacttttttacacgggtcgcggagacgaaactgtgtgtttcttttgcgactgttgcgtacgtgattggcatactaatgaagacacctggcagcgacacgccgccgaaaacccgcaatgttattttgtattgtcggtgaaaggtaaagaattttgtcaaaactcaattactgtcactcacgttgataaacgtgacgacgacaatttaaacgaaaacgccgacgacattgaggaaaaatatgaatgcaaagtctgtctcgaacgccaacgcgacgccgtgcttatgccgtgtcggcatttttgcgtttgcgttcagtgttattttggattagatcaaaagtgtccgacgtgtcgtcaggacgtcaccgattttataaaaatatttgtggtgtaataaaatggtgttcaacgtgtactacaacggctattatgtggaaaaaaaattctccaaggagtttttaattcatattgcgcctgatttgaaaaacagcgtcgactggaacggcagcacgcgcaaacagctgcgcgttctagacaagcgcgcctacaggcaggtgttgcactgcaacggcagatactactggcccgatggcacaaagtttgtctctcatccgtacaacaaatctattcgcacgcacagcgcaacagtcaaacggaccgacagctcgcatcgattaaaaagccacgtggtcgacaaacgaccgcgccgctctttagattctcctcgcttggacggatatgttttggcatcgtcgcccataccacacagcgactggaatgaagaactaaagctgtacgcccagagccacggctacgacgactacgacgacaatttagaagatggcgaaatcgacgaacgtgactctttaaaaagtttaaataatcatctagacgacttgaatgtattagaaaaacaataaaacatgtattaaaaataataataataaaactatattttgtaatatataatgtattttatttaaaaattgtctattccgtagttgagaaagttttgtcttgacttcataactctcttctccatattctgcagctcgtttacgttttttgtgacgcttttaattttctcaaaatgctggctgtcaatagttattttttgcttttgtctattaatttcttccaattgagattttaaatctcgctgagattgagatgcgttgtaattccttgagaacatcttgagaaaacatacagatgaggtaaaacagcatcttttatccaaattaggagttaattattattcatttgtatcgcgaccatttgctcgtacacatcttccataaaatggttatttttattgcgataagtgttggcattgacattttgcaaatgtcgtaggttaaaggggcaaatgggctgcgtggccgataaaagattccagttcaacaatccctcttcgcccccgtttaacttgaaaatggcgctacacgtttctacgctatcgtgttcctgttgagtggcgcacggttcgaccagtatcatcttgtgatatgcggttttgacattcatgtgcaacggaataacttgcgggtcatcgcattcgtcggaattaagctttaaatggcgtccgtatgctttccaaagtttttcgtcgtcgaaccgcggcactgcttgcaagtcgacgcggggaaacggcgctctgtacaaaacgcctaaattcaaaaactgattgcattgttgcagctctgtccaatcgacgcgatttttgtaattttgaaacagcatcaggttgaacgccgcgctggcgcgcacgtttgtaatcactgtgtaattgatcagcttgtgccaatactgggcattgaaattttcttcaaactcatttctaaactctggatgcgcaaacatgtgtctaatgtagtacgcgggcggggcgttgaacgcagtccatttgtcaatacacttccagtccgaatgtaacgtgttcaccaaaccgggatattcgtcaaacacgagcatgtgatccgaccacggtatgctgtgggcgatcaattttagttcttgcacgcggccttcgcgtaagcaatacaaaatgagcgcgtcgctgatcttgacacagtcttgcatgtacgcggacaaattaacgttttccatacagctcacattgtttattagcgccgtgttcaagtgtttgtatttggacacataatcgtagttgatgtactgtttaatgggttcttgaaaccattcttttagtagtatgtgactggccactatgcgtttccaatttaatttgtgtgcgtatttttgctgcaccgacaacgagaggttattgtaatttttggatatttcttccatgtccaacaagtccccaaacgcgagtataaaatcttgcgtcaaaaatttttgctcagacaccaacgaccagatcaaatgtgatttaaacctgttggcgattgttatcgacaacggcgaaattgaaataattttccaatccaacttgttgcgaaacacgtgaataaaatcgacgcgtccgtaacattcgcgcgatatgcgcttccaaaacgtgtcatcttgcaaattaagcaaatagacacgattgttgggagatttgacggccaattcaattatttttatatattctttttgctttaaagcgcgttgtagcacttgggttggagccatgtcgactgaagctccacgctgtttgaagcaaggtgaccgttttggtcggcatgttcaaacgtcgattacatgtttgctttgcatcaaaatggcgtaattaattaagaaacaacatgaaagccatctgcatcattagcggcgatgttcatggaaaaatttattttcaacaagaatcagcgaatcaaccgcttaaaattagcggctatttgttaaatttgcctcgaggtttgcacggctttcacgtgcacgaatatggcgacacgagcaacggttgcacgtcggccggtgagcactttaatcccaccaatgaggaccacggcgctcccgatgctgaaattaggcatgttggcgacttgggcaacataaaatcggctggctacaattcactgaccgaagtaaacatgatggacaacgttatgtctctatatggcccgcataatattatcggaagaagtttggtcgtgcacacggacaaagacgatttgggccttaccgatcatccgttgagcaaaacaaccggcaattctggcggccgtttgggatgcggaataattgccatatgtaaatgatgtcatcgttctaactcgctttacgagtagaattctacgtgtaaaacataatcaagagatgatgtcatttgtttttcaaaactgaactcaagaaatgatgtcatttgtttttcaaaactgaactggctttacgagtagaattctacttgtaacgcatgatcaagggatgatgtcatttgttttttaaaattgaactggctttacgagtagaattctacttgtaaaacacaannnnnngatgatgtcatttgttttttaaaattgaactggctttacgagtagaattctacttgtaaaacacaatcgagagatgatgtcatattttgcacacggctctaattaaactcgctttacgagtaaaattctacttgtaacgcatgatcaagggatgatgtattggatgagtcatttgtttttcaaaactaaactcgctttacgagtagaattctacttgtaacgcacgatcaagggatgatgtcatttatttgtgcaaagctgatgtcatcttttgcacacgattataaacacaatcaaataatgactcatttgtttttcaaaactgaactcgctttacgagtagaattctacttgtaaaacacaatcaagggatgatgtcattwtamaaatgatgtcatttgtttttcaaaactaaactcgctttacgrgtagaattctacktgtaaaacacaatcragggatgatgtcatttactaaaataaaataattatttaaataaaaatgtttttattgtaaaatacacattgattacacgtgacatttacgatggcgaacaataatttcactttttatattaggacacgacgtgtatataggaaagcttaagcgtttcaataaagccatggcgtacacgctaagcttgcccagcttgcggctctttgaaatctgtagttttcggggagtaccgtcgttcttcagtgccacatacgtcaacttgcgatcgtacactttataatacgtgttgtagttatttttttccagaaattccctcataaagcaatccttggataaagtttttgatccgtacagttggccacaccggtccatgcacaggtacacacacgtgatggcgttttgaatgacgatgcgatttctgtcaacggcaacgcgcttgaatatggtgtcgacgttgtccgattcaatggttccgtaaacagctccgtctggatttactgccaaaaactgccggttaataaacagctggccgggaatagacgtgcccgtgatgtgtgtcagcagagctgagcagtcagccatagaggctagagctacaagtgccagcaagcgatacatgatgaactttaattccccacagcaaactggcgcttttatataaaaatttgggccatttttggcgattagataatttttgaagattagataatattgagattagttaataatttgtgtgattagataactttttagggtattgcgcattataaatcaaggtcgagttgtataaactgctctggcgtgtaaaactgcagacttaagttttttgcaaacactcggtctgaatcgctaaaatctttctgaccggtggttagattaattcggccagccgcgtcgcccacataaaaagattgttccttgtcaatatgcgtaaactgtttggccatctcgcgccacattcccgtgtcgggctttcgatgctcatccttgttgggcgacacataaaacgatatgggcacgccagtagcttttttaatattctctaatttatataataaatcgctcgctttgattttgccggaacctaaatgggcttggttcgtaaaaacaactaaatcgtagcctaattcgtacaaacgctttagcttgtgtgcgcacggaaggagctgccagtcgtctgggttttttggaaatttggaccgtgtctttgagctaattagcgtgccgtccaaatcaaaagccgcaattttggttcttttagcgccgtcatgaaccgcgtacgcatacaaatcgggctgctgtaacgtccacatggtgaatgcatcttactcaaagtccatcaattcgtacgcgtttgtgtccaggtcgggcgttgaaaaattgtagcttgccattagatcggatagcgattcaaattttgtaagcgtttgtagcgcacgtttggcatcttgtttaaaattacacgacgacagacagtaaaaatattcctcgataagcatgactacacccatatcactgtttaagtgctcgacgtagttgttgcatgttatgtcgcgtgtgccgcgatacgcgtgatttcggtgaaaatcacaccacaaccagtcggcgtgcgtgtaacaaagtcgacagcgaaacaatttatcgttttccaaaaaattcaagtactcgacagttttgcagctgagattgcgcgtttgattcaccttaaaatcgtcgtcagcctctataatctcgggcaacagcttgccttgttggcccatcgtatcgatcacctcgcccaagtggcccggtgttatattaagtcgtttgaaagcatctatcgcttcttgcacgtcggcctgataatttttgaccacgggcgtggaaatcaattgccgttgaagggaaataattcgtggtgtgggtatcggccgcctgttgcacaattccaccagcggtggaggcaagggcgcattcacagcaaccgttgtcatttataagtaatagtgtaaaaatgcaaatattcatcaaaacattgacgggcaaaaccattaccgccgaaacggaacccgcagagacggtggccgatcttaagcaaaaaattgccgataaagaaggtgtgcccgtagatcaacaaagacttatctttgcgggcaaacaactggaagattccaaaactatggccgattacaatattcagaaggaatctactcttcacatggtgttacgattacgaggagggtattaataataacaataataaaaaccattaaatatacataaaagttttttatttaatctgacatatttgtatcttgtgtattatcgctaaccattaaaagtgctggagccacagtgttgcggcgagtctttatagaagatcgttgtttggctggaactgagcttttccttttcctgctgccgctaatgggagtgggcacgtactctgtagtagacggtgcaacgggcaacttgagcgctaccgtcttaaatttggccatacttttagtgatgaaatcgcgcgttaacacttcgtcgtaaatgttacttagcagaggcgcaacattgtgattaaatgtctcgtttaacaagctgtaaaactccgaataaagcttatcgcgcatttcgcagctctccttcaattctgccaaatttgcgttggtaagcaccacagtctgtctttttttgctcgctggaattgctgcgttctcgcttgaagacgacgatgtcgatcggtcggccatttttttgcccagcttttcagtgtgatcaaaaatgaacacaaaatctgccaattcgggcttgtttttcaccaaatcccacatggccgggctactaggccactcgggctgcttgatcttagtgtaccaactgttaaacaaaatgtatttattgttgttaatcactttcttcttgcgtttggacattttgcgttcgtcttgcatgacaggcaccacgttaaggatatagttaatgttctttctttccaagaaatttacaataacggccagctggtccatgttggatttgttgtaagagctcgattccagtttattcaacagcttttcatttttgcacacggccgcagtctccggagattgttgctccggcacgtttaccatgtttgcttcttgtaaacctttgaaacaacccgtttgtattcttgatgatatatttttttaatgcccaacaacctggcaattcgtttgtgatgaagacacaccttacgcttcgaacatttgtcggtgattactgtgaaatggcctaaattagctcttatatattcttttatacgctcaaacgacacgatgtccaacatgtgcgcgcagacgttttctgtgttcatcgtgtgcttgagcgtgttgatggcttccctgaacagcgcttgtatttcgctgcgagtcaagcagtccgaatcacacccgcctaagtgcgtgcaatttttggggggcatcgttgtctatctttttcagagtggcgtagaaaaagtcctgcaattgcctattatcaaaacgcgccttgacgctgcgcacaaaatcaaaaaattcaatgtaattgctgtaatcgtacgtgatcagttgtttgtcgttcatataattaaagtatttgttgagcggcacgatggccaggctgcgcgctatttcgcaattgaagcgtcgcggttttaacattatacggtagtcattgccaaacgtgcccggcaacaacttcacggtgtacgtgttgggtttggcgttcacgttaatcaagttgccgcgcacgacgcctacgtatatcaaatacttgtaggtgacgccgtcatctttccattgtaacgtaaatggcaacttgtagatgaacgcgctgtcaaaaaaccggccagtttcttccacaaactcgcgcacggctgtctcgtaaacttttgcgtcgcaacaatcgcgatgacctcgtggtatggaaattttttctaaaaaagtgtcgttcatgtcggcggcgggcgcgttcgcgctccggtacgcgcgacgggcacacagcaggacagccttgtccggctcgattatcataaacaatcctgcagcgtttcgcattttacatatttgacacttaaaaaattgcgcacacgagcaccatcgtttgatacctaattgcaactatttacaatttatcagtttacgttgaacccgttttaattttttagatccgtccttgttcagttgcaagttgactaaatgacaaaatttttcggttctgcaaaaccgcccttgtctgttccacccgttgtatttgaaaaaactttttttcacgcggcgacaactgcttgtataatattgcccaatgtaaacatgcaaaattttgttactctcgtcaaaacagcggttggcgttccattccataatttttttattatttatcaacgatggccattgtaaattgtcgtcatttatacgcatcatatgatttaacaaaagcttttcgtatagcggaacttcaattcccttggaacatttttcaaacgataatttaatttgtttctcggttggcagcatttcatgcttgattaacaatcgcctgacttttatagccacgtttatgtctttgcacagcaaatgtgggttgtcgacaatgtaatagtgcaaagcatttgttacggcaaatgcgtagtttgatttgacgacgccctttttcttgacgggcattgcggcttttaaaattacttgcaagcattgtacgaatacctctttgtgtttaaacaataatatggacaaacatcggcgaaacaatttgtaataattatgaaatcccaaattgcaggttttaaacttctttgttacttgttttataataaataaaatttgctgacccatgtctgcgcccacaactttaattaaccatttgtgcgcatattgattgtctcgttgttcccaaccggaaaattgattgatctcgagccaccggcattggtcgtttgataccgtcgttaacgccgacgctcctgcctgtttgattacgggttctaaaagacgaaacagcagcgtaaatttgtttttgcgtcggtagtattttggcaggcaataatcaaaaaaatccgtaagcaattctctgcatctattaatattcgttgcgtacgaatcgagtttttcaaaaattactttgtttgtatgaaaataacgtttgggcttctcacaataataatcttcgttgtagaacagaaacggtttgcgagaattggcacgtttgtccatgattggctcagtgtaacgattgattcaaatcaaaattgacaacacgtttgccgtaatgtgcaccggttcgcacacgtttgccgcgtatgtaatccatgtttatttcgctgtcgcaattgattacacgattgtgttgggcggcgcgttttattgaatttaggcgacgcgtcgacaactccaaaggattgtaaagcgcagatttttccagagtaaacgagtttaagtggccaccgttgaaccattccagagccacgattgtgtacagcaaaaagaatatttctttgtcgacgttttcaaacgcaaacttgttttttaggcaatagtagtaaaattttaacgaattgtataaataaaacataaaattgccatttttaaagtaaaattctacatccgtgacgaacaaaaggtttactattttgttctccaacaagtgtgccaattttcttaagtacaccattgaatttttgtcgtcgtccatctcgatcaacaacacgtacggcgttttggaatttaaaattattctaaaattttcctgttgcaacgattccacagcgtccgaccaatatgacgctgccacctctagacagatgtatttcttggaaaacacgtgtcgtttgataacctcgctgatggacgtgatcgattgtaaatacttttcaaacgtcgcgtcttcccaaccacgcaccgacacgggcgctgtcgtgtcgggctgatgtttgaaatccaaaccactctgaattaacttggttgtgattcgtatgctcaactgttgacccaacgtgtagtgatcttcgtaggcgcgctcccacatcacgttacacacaaatttgacgagatcatcaacgtctttctgttgcaaaattcgccgcaaacgcgccacatcgcccttgtaccaccgatctcggcacacaagctgtagcattttcaaatcgtgatcgctcaagctattaattctggttagatttatatagtcgtcaatatcctcgggcgtggtttgcgtcatgtctgtaaaacgtgcaaaatcaaatatttttatgttgtagtcgaatctaacaaatccatcggcgttcacttgcacttcgcgctttacaaaacgaggtagcgtgtaatcgaacccgtttaaatagattacgtacaaaaccagcacttcatcttccagtttgcacgcttgcggcaaaaattgtgtggtgtgctccaaccgggtgacaaacatgactatggaaaatgacgcggaattcaacagacgactagagtacgtgggcacgatcgccacaatgatgaaacgaacattgaacgttttacgacagcagggctattgcacgcaacaggatgcggattctttgtgcgtgtcagacgacacggcggcctggttatgcggccgtttgccgacctgcaattttgtatcgttccgcgtgcacatcgaccagtttgagcatccaaatccggcgttggaatattttaaatttgaagaaagtctggcgcaacgccaacacgtgggcccgcgttacacgtacatgaattacacgctttttaaaaacgtcgtggccctcaaattggtcgtgtacacgcgcacgctacaagctaacatgtacgcggacgggttgccgtattttgtgcaaaatttttcagaaacaagctacaaacatgttcgtgtgtatgttagaaaacttggtgcgatacaagtaacaacattaccagtttacgaacaaattattgaagatacaataaatgaactcgtcgtcaatcacgttgattagataatgtccgtgttaaatgtgatatcttagattacgagcgcgcaataaccatagtttaatcgaagagaatagccgtcgccacaatggataattacaaattgcaattgcaagaattttttgaccaagcgcccgacaacgacgatcccaactttgaacatcaaacgcccaatctattggcgcatcagaaaaaaggcatacagtggatgattaacagagaaaaaaacggccggcccaacggcggcgtgcttgccgacgacatgggactcggcaaaacgctctctgtgctaatgttaatcgcaaaaaacaactctctacaattgaaaactctaatagtgtgtcctttgtctttaatcaatcattgggtaaccgaaaacaagaagcataatttaaattttaacattttaaagtattacaaatctttggatgccgacacgtttgagcattaccacattgtggtgaccacgtacgacgttttattggcacatttcaaattgatcaaacaaaataaacagtcaagtctgttttcaacccgctggcatcgagttgttctagatgaagcgcatattatcaaaaactgcaagacgggcgtgcacaacgccgcgtgcgctttgaccgcaacaaaccgatggtgcattaccggcacaccgatccacaacaagcattgggacatgtactcgatgattaattttttgcaatgtcgtccttttaacaatccaagagtgtggaaaatgttaaataaaaacaacgactctacaaatcgcataaaaagtattattaaaaaaattgttttaaaacgcgacaaatctgaaatttcttctaacattcctaaacacacggttgagtatgtacatgttaattttaatgaagaagaaaaaacgttgtacgataaattaaagtgtgaatcggaagaggcgtatgtgaaggctgtggcagcgcgtgaaagcgaaaacgcactaagccgattgcagcaaatgcagcacgtgttatggctaatactgaaattgaggcaaatctgctgccacccgtatttggccatgcacggtaaaaatattttggaaacaaacgactgttttaaaatggattatatgagcagcaagtgcaaacgagtgctcgatttggtagacgacattttgaacacaagcgacgacaagataatattggtttcgcaatgggtggaatatttaaaaatatttgaaaacttttttaaacaaaaaaacattgctacgttaatgtacacgggccaattaaaagtggaagacaggattttggccgagacgacattcaacgatgctgccaatactcaacatcgaattttgctgctttccattaagtgcggcggcgtcgggttaaacttaataggcggaaaccacattgtaatgttggagcctcattggaacccgcaaattgaattgcaggcgcaagaccgaatcagtcgtatgggacaaacaaaaaacacgtacgtgtacaagatgctaaatgtggaagacaacagcatcgaaaaatacattaaacaacgccaagacaaaaagattgcgtttgtcaacacggtctttgaagagactctgctcaattacgaagacattaaaaaatttttcaacttgtagctggtaagtcgtcatgaacacccgatatgctacttgctatgtttgcgacgagttggtgtacttgtttaagaaaacgtttagtaacatgtccccttcggccgctgcgttttaccaacggcgcatggccattgttaaaaacggtatcgtgctgtgcccacgttgttcgtcggaactaaaaattggcaacggcgtttcgattccaatttacccccaccgcgctcaacaacatgcacgacggtcgcgttaagacgcaagcgcttcgagttttggcccgctcgctacctccgctgtacgactcgaccgtcgatcgacacggctgcaaggtgttcacggtgcggcgctacaacagacgcgtaatcgactttgcgggcattcgcaacaaaacgctggaaatcattaaaacggatagaaacttgccgctcaacacagaatgcaatgtgaaagttgtcgacagtgcatgcatgcgttgcagaaaaagtttcgcagtttaccccgccgttacctatctgcattgcggacattcgtgtctgtgcaccgactgcgacgaaacggtaaacgtggacaacacgtgtcctaaatgtaaaagcggcattagatataaattaaaatacaaaactttgtaacatgttgccctacgaaatggtgattgccgtgttggtttacttgtcgccggcgcagattctaaatttaaaccttccttttgcataccaaaaaagtgtgctgtttgccagcaactctgcaaaagttaacgaacgcatcaggcggcgagcgcgtgacgacaacgacgacgacccctatttttactacaaacagttcataaagattaattttttaactaaaaaaataataaatgtttataataaaactgaaaagtgtattagagcgacgtttgatggtcggtatgtggttacacgcgacgttttaatgtgctttgtaaacaagagttatatgaagcaattgctgcgcgaggttgacactcgcattacactacagcaacttgttaaaatgtatagtccagaatttggtttttatgtaaatagcaaaattatgtttgtgttaactgaatcggtgttggcgtctatttgtttaaaacactcgttcggcaaatgcgagtggttggacaaaaatataaaaactgtgtgtttacaattaagaaaaatttgtatcaataataagcaacattcgacatgtctatcgtattgattattgtcatagttgtaatatttttaatatgttttttgtacctatcaaatagcaataataaaaatgatgccaataaaaacaatgcttttattgatctcaatcccttgccgctcaatgctacaaccgctactactaccactgccgttgctaccaccactaccaacaacaacaacagcatagtggcctttcggcaaaacaacattcaagaactacaaaactttgaacgatggttcaaaaataatctctcatattcgtttagccaaaaagctgaaaaggtggtaaatcccaatagaaattggaacgacaacacggtatttgacaatttgagtccgtggacaagcgttccggactttggtaccgtgtgccacacgctcatagggtattgcgtacgctacaacaacaccagcgacacgttataccagaaccctgaattggcttacaatctcattaacgggctgcgcatcatttgcagcaaactgcccgatccgccgccgcaccaacaagcgccctggggcccggtcgccgattggtaccatttcacaatcacaatgcccgaggtgtttatgaacattaccattgtgctaaacgaaacgcagcattacgacgaagctgcgtccctcacgcgttactggctcggcttgtatctgcccacggccgtcaactcgatgggctggcaccggacggcaggcaactcaatgcgcatgggtgtgccctacacgtacagtcaaatgttgcgcggatattcattggcgcaaattaggcaagagcagggaatacaagaaatcctaaacacgatcgcgtttccgtacgtgactcaaggcaacggcttgcacgtcgattcgatatacatcgatcacattgacgtgcgcgcttacggctatttgataaattcatactttacgtttgcctattacacgtactattttggagacgaggtaatcaacacggtgggtttgacgagagccatcgaaaacgtgggcagtcccgagggagttgtggtgccaggcgtcatgtctcgaaacggcacgttgtactctaacgtgataggcaactttattacgtatccgttggccgtccattcggccgattactccaaagtgttgaccaaactttcaaaaacatattacggttcggttgtgggcgtaacgaataggttggcttactacgaatccgatcccacaaacaacattcaagcgcccctgtggaccatggcgcggcgcatttggaatcggcgcggcagaattatcaactataatgccaacacggtgtcgtttgagtcgggtattattttgcaaagtttgaacggaatcatgcgcatcccgtcgggcaccacgtccacgcagtcgttcagaccgaccattggccaaacggctatagccaaaaccgacacggccggcgccattttggtgtacgccaagtttgcggaaatgaacaatttgcaatttaaatcgtgcacgttgttctacgatcacggcatgttccagctatattacaacattggcgtggaaccaaactcgctcaacaacacaaacgggcgggtgattgtgctaagcagagacacgtcggtcaacaccaacgatttgtcatttgaagcgcaaagaattaacaacaacaactcgtcggaaggcaccacgttcaacggtgtggtctgtcatcgcgttcctatcacaaacatcaacgtgccttctctgaccgttcgaagtcccaattctagcgtcgaactagtcgagcagataattagttttcaaacaatgtacacggccacggcttcggcctgttacaaattaaacgtcgaaggtcattcggattccctgagagcttttagagttaattccgacgaaaacatttatgtaaacgtgggcaacggcgttaaagccctgtttaattatccctgggtaatggtcaaagaaaataacaaagtgtctttcatgtcggctaacgaagacactactataccatttagcgttataatgaattccttcacctctatcggcgaaccagctttgcaatactctccatcaaattgctttgtgtatggaaacggtttcaaattgaacaacagcacgtttgatttacaatttatttttgaaattgtgtaattatatttagggagaatgtgatattcaaaagactgactgttaacacaaaagactgatattgttgttgttacaaaatagataataaaacaaaaaataaattaaatattatttatttattaaactgtttaattttaatgctaacgcgtacaaatcacgctgttccgacgtggacatggaattgcgcagaaaagtcttgatagtgtcgatttcttcgccgtcatccacttccatatatttgatttcttcctcgatttgcatttccaagtttgcgtattcttgcaaataataatctagtcgttgggcgacctcgccaattttaaataatacattatccgacaccaaatgccagcgagtgactgtgcgctccatcatcctggcactttttaatgtgaatattaaaaggttgttgcatatatatcgttaaacgtttatgtttactttcacgttagctcgtttcattgatgtaaacatttagttttataacagcgtcggtaattttattttttaaagtaaacagaccaaaatcaaaggtgtcttcgacaggtacgattattttcccattgacactgttttcgtgcacagatataattttatcaccgtttattattttgcccaaacacacgtactcgtttcttctcaagccaactatttctaaacaattcacttttctattatcgtgtacgcaattaaaagtaaacgaagcgctacaattgtcgtattctattacaattctgcggcatttataaaatttattaatgttgacgcaaattccatgcagcgcatccatttcgtactgcaaatgcggcgcaattaaaaaatttcctcgtcgttgttaacaatcttgggcgctaaaaagcacgccaacacgcccacgtctttaatgcaatattccaatttgaacggcagttcctcggacatgtatattgtcacggtgggcgccaaaggagcggctttagcaaaatgacacaagtaatcgcccgcaaaagtgtgcgttacggtttgctttgctttgagaacggaaaagttttcgttgtccgcgctcatctgcacgtccgccgagccaatgtcgccatttgctctaaactgcagacccttcttggaacacgacacaataatatcgtggtcgaattgcgtcatgtctttgcacacctgcgcaaactcgacgctcgacatgtggacgacgcaatcgtaatcgctatccggaattcccaaatgttccacgtcgatgcacatcaacttgagcgtgtacgtgcagattctattgtcgttgttgaacacgaacgccatcacatcgccctgatcttccgctttcatcagtacagagctgcgctcgttaacgcatttgacaattttacttaaactgtttatggacacgttgagcgtcacgttgcggtcacatctatattttttgaaaccctcggcgtgtagttgcaacgacacgagcgcgacatgcgaggtgtccataacctgcatgcttacgcctcgattatcacaatcaaaagtagcgtgcggcagcagatccttaaaagtttccaccagcctcttcaaaactgcgccggttttaaattccgcttcgaacatttttagcagtgattctaattgcagctgctctttgatacaactaattttacgacgacgatgcgagcttttattcaaccgagcgtgcatgtttgcaatcgtgcaagcgttatcaatttttcattatcgtattgttgcacatcaacaggctggacaccacgttgaactcgccgcagttttgcggcaagttggacccgccgcgcatccaatgcaaactttccgacattctgttgcctacgaacgattgattctttgtccattgatcgaagcgagtgccttcgactttttcgtgtccagtgtggcttgttttaataaattctttgaaaatattgtcgggtgtattattaaatagcatgtatggtatgttgaagatgggataacgcttggcgtgcgggtcgtcatgatttccaccgcgcaccacatatttgcgctcaattttatcaaaattggactggcgagacaaaaacgagacgggcgacaggcatatttgggcgtgcgtaccatcttcggccatccactcggtcaggtcttcgctgcggttaaacacacctttctgaccgtgaatgccacatatttttattccttccaaatcgttggtggacgtgactatgactattttaagcataacgttgtcgccgttaaccaccatgctggcgtcgagtttttcaattttttgatttttaatttgtctaaagtaaacgtacactttgtaaacgttaaaattgccgttggtgcacgtttcaattttgtaccgtcggccgtcgtacacccaattaatctttgcgttgctcaccaacacaccggccatgtacagcacaagtccgtcgtctagcgcaacgtaatttttgtcgctactattcgtaaactttactaaacacgactgcttggggccgaccacaagcttgcccttcaatttgttcactttgttgttgtataaacaaatgggcagcgcaatatgcggaatgtacggatcttcggcggtcatgagtttattgtctcgcaccaacgtccacaatttaaacattttattgttgagcaaaatggacttgtttaccgccacagagtagccatttggtaaacccgatacgcaattttcctctttgtactcaaacacgggcatggcattctttagattggttagggacacaatcaatttgggtacgggcgtggtatgaaataaatgtataaaattacgataataatactgctccaacttggacatgagcgatttgacgtcatcgttttctacgatcgtacactgaataatgggattatagtatatagaatgtttatagtggtattcgtagggtgtcaacaatacgttaatgtcggcttcgttgttcacccgcaacttttttttgatgcatatcattccttcgtgatgattaacgtaaagtattctgtctgtaatcttcaattcgatgggcgccatgtttcttttcatagtgtacacgataaacgacgtgtttgattttaaacattttaaatttgtgggtctatcattaaacgcgatcagcaacgagtcgtcttgaacgtcgttgaggtcgtccacgaacgcgaccagattgtgttttagcaaatattgaaatttttgcgcaaccatttcgtagtccacgttgggcaaacatgcgttgcggcaaaggaaaaactttttgcccgccacggtcatttcgccgtgaaaaaaactgccaataaatttcacaaaatcctttttttgcttcaacattttctggcgcatgctgtcgttggtgattcgcgccacctcgttgccgacgcgatattttaacacgggcaacgaaatttcaatattgttattgctgctgttgtcctgttgattgggaaagactttgcgttgcttgctaaaagttttcgatacgcaatatatgagacgcccgttgactatacaatcgacaatctttttcgactctttgttgtacaagacgctttgaattttacgacgcttgttcgccaccgtgtacgcgtcgtcgtcggccgtcttgtcgagaactcgttgatagttttgcaaaattgtcgaagttaataacagttctatcaaataggcgtgcttgtatacaattttgttggccaaactgtctatagaatagtttatgtcgtgattcataataatttttatgtgttccacgagttgttgcttgtgaagcgtgttgtattcgaagagaaaatcgagcggtttccatttgccgctgttggccagatatgtttccagcacagaatttaaatcttccgtcactacgtaatcgctagcgtacacgtctcgagcaaacaggacgtcgtcttgtttgtcgtaaactagttggattgcgcgattgatgtgcttctcttgatccacgttgccgtacaaaaacatgcgtttgcaatgtttggcgtatagcttgtcgtagaaattgtgcaccaaaacgttgttgttcatcattatgttgggaaaactcaaaaatctgccgtccagcataaaagttccgttaatattgttgtttgcgtcgacatcgtccgtttctctaaattgcttgtctaagcgcgtgccgaatataacgggcacacatttatgcattacgcaactgagctgttcattaagagcgcaacacaaataagacttgcgttcttgaatagcgcaaaaaagcatacgttcattgctgtttgtagcgcaatcaaaagtatattttaatttgtatttattttcaattctatcgtacaactcgttgaaatcttgaaccacgtccgtcatcgtgaagcgattactgcgcactaattatgtctaaacgtgttcgtgaacggtcggttgtttcggatgaaacggccaaacgcattcgacaaaacgaacactgtcatgccaaaaatgaatcttttttggggttttgcaacttggaagaaattgattattatcaatgtttaaaaatgcaatacgttccggaccaaaagtttgacaacgattttattttaacagtgtacagaatggccaacgtggtgacgaaacaagttagaccgtataacagtatcgacgaaaagcaccattacaacacggtgcgtaacgtgttgattttaataaaaaatgcgcgtttagtgcttagtaatagtgtcaaaaagcaatactatgacgatgtgttaaaattgaaaaaaaatacagacttggaatcgtacgatccattgattacggtctttttacaaattggcgaatctgtaaatgaagaaatacaaaaactcagaaaagctttggtcaatttttttactaataaacccgacaagtcggatataaacaacccagatgtagtttcgtatcaatttatttttggcagagtacaaaaattgtataacagggcaattaaacaaaaaactaaaactataattgtaaaacgtcctacaactatgaacagaattcaaatagattggaaaactctttccgaagacgaacaaaaaatgactagacaagaaattgccgaaaaaattgtaaagccttgttttgagcaatttggcactatattacacatatacgtatgtcctttaaaacacaaccgaattattgtcgagtatgcaaactcagagtcggtacaaaaagccatgactgtaaatgacgacactcgatttacagttacagagttttccgtggttcagtactacaacgtggccaaaacagaaatggtgaaccagcgaattgacataataagcaaggacattgaggatttaagaaacgctttaaaatcttacacataaattaaaatatcgaacaaaggaaaaaaacaattgtaacaaaaataatttacattaaaatttacaagtttttttctagtgtcgtacttttttacaatgcgtctgttgtccgtcgagcattgcaaacatattgtggacggcgcaaaatagcaaacaaaaggcacgtccgcgctctcccacgctattctaaaacgatgaatccatattaatttttcattgtcgccaaacgtcgctccgctgcctccttccaataacaaatactcagaaacacaaacatgtacaattgctgtcgcggcgttaattgtcgctgtttttccaaatagtctattatgggaaacaaacacttgtcacaacacaaatactcgttaattgtcacaaccgacaagcacatttggcaaaatgcgtcgcaatttttgtacggacgagattctatgcgaagttcgttgtccatgacgtcttgggtccactttttcaacaagacacttttatatttgtgatttgtacaactttggtacgtgttagagtgtttttgataagctttgataagtttaaaactgttggagtaaggccacgtcattatgttctgcaccttttgtttaaaagacagaaattactatatgttcaaactatttaaagattattggccaacgtgcacgacagaatgccagatatgtcttgagaaaattgacgataacgggggcatagtggcaatgcccgacactggcatgttaaacttggaaaagatgtttcacgaacaatgtattcagcgttggcgtcgcgaacatactcgagatccctttaatcgtgttataaaatattattttaactttcccccaaaaacactagaggagtgcaacgtgatgcttcgagaaactaaagggtttataggcgatcacgaaattgatcgcgtttacaaacgcgtttatcaacgcgttacacaggaagacgccctggacattgaactcgattttaggcatttttttaaaatgcaatcatgacgaacgtatggttcgcgacggacgtcaacctgatcaattgtgtactgaaagataatttatttttgatagataataattacattattttaaatgtgttcgaccaagaaaccgatcaagttagacctctgtgcctcggtgaaattaacgcccttcaaaccgatgcggccgcccaagccgatgcaatgctggatacatcctcgacgagcgaattgcaaagtaacgcgtccacgtaacaattattcagatcccgataacgaaaacgacatgttgcacatgaccgtgttaaacagcgtgtttttgaacgagcacgcgaaattgtattatcggcacttgttgcgcaacgatcaagccgaggcgagaaaaacaattctcaacgccgacagcgtgtacgagtgcatgttaattagaccaattcgtacggaacattttagaagcgtcgacgaggctggcgaacacaacatgagcgttttaaagatcatcatcgatgcggtcatcaagtacattggcaaactggccgacgacgagtacattttgatagcggaccgcatgtatgtcgatttaatctattccgaatttagggccattattttgcctcaaagcgcgtacattatcaaaggagattacgcagaaagcgatagtgaaagcgggcaaagtgtcgacgtttgtaatgaactcgaatatccttggaaattaattacggcgaacaattgtattgtttctacggacgagtcacgtcagtcgcaatacatttatcgcacttttcttttgtacaatacagtcttgaccgcaattcttaaacaaaacaatccattcgacgtaattgccgaaaatacttctatttcaattatagtcaggaatttgggcagctgtccaaacaataaagatcgggtaaagtgctgcgatcttaattacggcggcgtcccgccgggacatgtcatgtgcccgccgcgtgagatcaccaaaaaagtttttcattacgcaaagtgggttcgaaatcccaacaagtacaaacgatacagcgagttaatcgcgcgccaatcagaaaccggcggcggatctgcgagtttacgcgaaaacgtaaacaaccagctacacgctcgagatgtgtctcaattacatttattggattgggaaaactttatgggtgaattcagcagttattttggtctgcacgcacacaacgtgtagcatcgccagtatttaacagctgacctatttgttaaacaagcattcttatctcaataattggtccgacgtggtgacaattgtatccacaatcatgaaaaaagtagcgcttggaaaaattatcgaaaacacagtagaaagcaaatataaaagcaacagtgtgtcgtcgtcattgtcaacgggcgccagtgcaaaattgagtttaagcgaatattacaaaacttttgaagcaaataaagtgggccagcacactacgtacgacgtggtcggcaagcgagattacacgaaatttgacaaattggtgaaaaaatattgacatgctgcgatcaatcatgcgacgtttcaagagtacaaacaatctcagcaaaaaaccctccgattattatgtagtgttatgtccaaagtgttattttgtgacgtcggccgaagtgagcgtggctgaatacatagaaatgcataaaaattttaacacgaaattcgccgatcggtgccctaacgattttattgtgaccaactctaaaagttggaataatcatgaaaattgttctgccctattttaccctctgtgttaataaagtttgttgtttgtattttgtggttttatttatttacgctagatattgggtttaaggttcttagaaatagagttgtattttccctaccaaaagggatttgagcttcatataaatacaattttcgctcgacaagcggtttatttcactcggaggtattatatcaggcagtcgaacgtgcgcgatgaaacatcccgtttacgctagatatttggagtttgatgatgtagtgttagatttgactagtttaatatttttagagtttgataacgctcaaaatgaagagtacattatttttatgaatgtaaaaaaggcgttttacaaaaactttcacattacttgtgatttgtcgcttgaaacgctgaccgtgttggtgtacgaaaaagctcgcctaattgtgaaacaaatggagtttgagcagccgccaaactttgttaattttatcagtttcaacgcgaccgacaacgacaactccatgataatagacttgtgttccgacgcgcgcataatcgtggccaagaagctgacgcccgacgaaacgtatcatcagcgcgtgtccggatttttggattttcaaaaacgtaactgcatacctcggcccccaatcgagtcggacccaaaagtgcgagacgccttggatcgtgaactagaaataaaactatacaagtagaaaaaaattaatttattaatagttgtaataattatcttcgtcctcatcttcgctggtgtcataatgcggtggtgtgtttgtgttttgttttaatcgtttgcgcgtcgacaccacttcgccgataggaaattttttggatttcgcattaaatgcccgcttagcgacgcgccgtttacgactactaaacatgttgacgcgctcgtcgtcttcagtgtcataatccgtgctagtgttttcgttgttattttctatgagacgatcgtttgatttagttttcgtagaattgtccgcgttatcgtcgctttcgtcgatgtcgtccctaactatctcgtaggcggctttgcgcggaatccaagattttgcaatgtatctattttaacgtacttttcttcgagcgcttttctagctttatgcatagcaatgtcttcgtcgccgccgttcattttatgatactttgtaaacgtctcgacgaataactttttggcgcgaggaggcattttttcattgtataacatatcgggaatttgatacattgtaattagaattaagcaagttcgtcttcggttgtactgtattcggtttctgtatctgtagtggaatcctctgtactagtagtagtgtcgctattgttggcgtcaggccttggctgccatttaccgtctatcaacatgtattttttcctaacagcacaacatgctagcttggtagctatctgtgtcgacttatatttttgtaaactacgatcgtagaatttttcaaatatcctcttaccgttatagggaaggttttgataatatttaggcaacatatcaataaaagacaatataaaaactttgtgtttgtgttttatttatcacataaaatggacgtctggcaagaatcacaaccaatattagtgttttttttcttacattacgagattcaacttgatactaaaattaattattaattaaattaaattaaattttgaagcattttttcgctatcgttttcagactcaaaattatcgacgctatcgctatgaaaagcgtaatatttgttggctttgagatattctatattttgctcatttttaacaataaacacgcgactcttttcgtcgcgtctcaccataacaccgtttttacaaatggaaatgtatttgtaaaacggcaacagagcgtcgcgagtttttttaagtaacagcttttgctccgctgtggcggccacaaatatttttacgggcccgtcgtaattaatgtttaaattaaaatttttaagtcgacgctcgcgcgacttggtttgccattctttagcgcgcgtcgcgtcacacagcttggccacaatgtggtttttgtcaaacgaagattctatgacgtgtttaaagtttaggtcgagtaaagcgcaaatcttttttaaataatagtttctaatttttttattattcagcctgctgtcgtgaataccgtatatctcaacgctgtctgtgagattgtcgtattctagcctttttagtttttcgctcatcgacttgatattgtccgacacattttcgtcgatttgcgttttgatcaacgacttgagcagagacacgttaatcaactgttcaaattgatccatattaactatatcaacccgatgcgtatatggtgcgtaaaatatattttttaaccctcttatactttgcactctgcgttaatacgcgttcgtgtacagacgtaatcatgttttcttttttggataaaactcctactgagtttgacctcatattagaccctcacaagttgcaaaacgtggcattttttaccaatgaagaatttaaagttattttaaaaaatttcatcacagatttaaagaagaaccaaaaattaaattatttcaacagtttaatcgaccaattaatcaacgtgtacacagacgcgtcggtgaaaaacacgcagcccgacgtgttggctaaaattatcaaatcaacttgtgttatagtcacagatttgccgtccaacgtgtttctcaaaaagttgaagaccaacaagtttacagacactattaattatttaattttgccccactttattttgtgggatcacaattttgttatatttttaaacaaagctttcaattctaaacatgaaaacaatctggttgacatttcgggcgctctgcagaaaatcaaacttacacacggtgtcatcaaagatcagttgcagagcaaaaacgggtacgcggtccaatacttgtactcgacgtttctcaacacggcctcgttctacgccaacgtgcaatgtttaaatggtgtcaacgaaattatgccgccgcggagcagcgtaaagcgctattatggacgtgatgtggacaacgtgcgtgcatggaccacgcgtcatcccaacattagccagctgagtacgcaagtctcggacgtccacattaacgagtcatctaccgactggaatgtaaaagtgggtctgggaatatttcccggcgctaacacagactgcgacggtgacaaaaaaattattacatttttacccaaacctaattccctaatcgactcggaatgccttttgtacggcgaccctcggtttaatttcatttgctttgacaaaaaccgtttgtcgtttgtgtcacaacaaatttattatttgtacaaaaatattgacgcaatggaggcgttgtttaaatctacaccattggtttacgcgctgtggcaaaaacataaacatgagcagtttgcacagaggctagagatgttgttgcgtgatttttgcttaattgccagttcaaacgctagttatttactttttaaacagcttacacagctcatagctaacgaagaaatggtgtgcggagatgaagaaatattcaatttaggcggccaatttgtagacatgattaaaagcggtgctaaaggcagtcaaaatctgattaaaagcacgcaacaataccgacagactttaaatacagatattgaaactgtgtcttcacgagccaccaccagtttaaatagttacatatcttctcacaataaggtaaaagtgtgtggcgccgacatatatcataacacggttgtgttacagagcgtgtttattaaaaataactatgtttgttacaaaaacgacgaacgtacaatcatgaatatttgcgctttgccctctgagtttctgtttccagaacatttgctcgacatgttcattgaatgataatataaatagagcgcatttgattgcatgcaatcagtgttttattaattttagagcaacatgtacgataaatttatgatctatcttcacttgaatgggctgcacggagaagcaaaatactacaaatatttaatgtctcaaatggattttgaaaatcaagtagccgatgaaatcaagcggttttgtgaaactcgtctgaaaccggcaatcagttgcaacactttaactgcggaaagtctcaatacgctcgtagacagcgtagtctgcaaaaatggactgttaaatccttacgccaaagaagtacagtttgctttgcaatatctttttgacgatgacgaaatatccaaacgagatcaagatggctttaaactatttttattacataattatgacaggtgtgaaaatatggaagaatattttttaattaacaattttagcatagcagactacgaatttgaagacatgtttgaaattgttcgtattgattgtagagatctgttattacttcttgctaaatataatatgtaattaaaattttgtttgttttattaaaatcctggattaaaaaatgacgaataatttgatttgcgtgcacgccaacaagattcttcgtcattatgatcaatgcgtgcatcaagtttatgcttttgtaattggcttctgaccactttagccatttgagcgtatctgcattcgtcgtctagagtttcaaacaccagatcggcgcaattataaaatccttcacccacgggatctatgcgctgccaacgcacatacattacaaattgatttgacctgtacggtattactacgggtatagaatagactagactgttgtcacataatgaatcgcccggatttggaattaaatttgaatcgttaccacctatgtattctaattcgttccaagttattggattgcgacgatcccagtttgatttagtaataaacacttcaaaataactgggctcgtgtatggctgttggacaaaaatgaacattcatctgataaaccggttgatagcgatttaaatatagcgtatttggcctccagttgttaaaaggttcgtccattccgcttttatcaccaaacacagaattgcgatcgtttgaaccggcaccgcaaagtgtgtgcggcacaaccctttgtttgattaggtcaaaatcgtcataattaggaccggccacagccgcgtattccatatactgttgaaacatgtattgcgctgtggaagcggccgccccggattctaaatcgagagctcgatatttataatagactgatttgtaagcattgcggcacgcggcgtcgggaatgttatcgccattgtcgggccaataaaagtttccatctttaaaacatttatattgacgggccgtcggcacggacaaatagccgtgagagcgcactgccggcgcgtgaatcgcagcaaacaatgcaattaataatgcaatcattatgattatacttatagaacactaatcggaataataaccgctgtcgtaatcttggtcaaaaacgttatgttgaaacataataacaccttacagtaacatacaataaaacaacatagtatcgtatataattataaactttattttttcattttatacaaacaaaatttatacgtattgttagcacattgagtgtcattttcgctgtctgaactatcacaatcatcgtcatcatcatcatcattgtcatcgtcgtcgtcacgtttgcgtttgacactgcattttttttggttaattttcactaacactggttcttttcgatcgtacaattgattctgcatgtacttttgcatgatcgcggtaaaacactttgcaattttatccttttgttcgtcgccaaatatttccagcaactcgttcataaatgtgcacaaaatgcccatgtgttttatccagctgattcgcattttcactggatcgaacaaacgcaaggggtacgctttttctgttaccttgccttcgatgtctatcaaaaggtacgggatacgatctccgttgccgggcacaaaatccgtgcctttgttaaccaaaatttctctacaatgcctagccaccgtaatcacgcgtcttttgggtgacggaccctcattatcgtcagttgatttgcgttttttgcccgggttatcgttataggtcatactaaagctgtagtcggtcaacgattttgatttggcaaactcatcatagtattcataaaaactagtctgtaaactttgcaaacatttgtccatgtccaaatgacgcaatatttgttccactgccgtcctaaacgcgattctcataaaaacgggcatatcctttttaactaaccaacccttgtatacgattttattctcactgttgagatagcaatattttttcttttttaatagtattaaaactttcattaaattttcaaatgccattttgtaaccgtccgtgaatgagttattaacgcgtgtctcaacatgtgtgcatatttgttttaatgtgtcggtttcgttggatatttcgttatagttaaatgtgggcaaaacaaatgtagaatctgtgtcgccgtacacaactttaaaagtgatgctgcccagattgaatttttctaaaatctcagggtcgttgctcaaaccttcaatcagagaaatggccagccgcaactgattgcgaccaactctagtgatgtagtttgcaagcactttgtaaaaaatgccataataaccgtatatgctattggcggtgcgcttcacggaattttgtttttgatcgtacagatcgtacaagaatgccgattcgctttgattgtcgcgattctttttaaatttgcacctttcgcttaacaattttaatagcaatttaacaactattgcacgcgaattgtggttcaaatacacgttgccgtcttcgcataaaattaaattggacaaacaagcacaaatggctatcattatagtcaagtacaaagaattaaaatcgagagaaaacgcgttcttgtaaatgcctgcacgaggttttaacactttgccgcctttgtacttgaccgtttgattggcgggtcccaaattgatggcatctttaggtatgttttttagaggtatcaattttcttttgagattagaaatacccgctgcggctttgtcggctttgaattggcccgatattattgacagatcgtttttgttaaaaaaatacgggtcaggctcctctttgccggtgctctcgttaatgcgcgtgtttgtgatggctgcgtaaaagcacgccacgctaatcaaatgcgaaatattacatatcacgtcgtctgtacacaaacgatgcaatatacattgcgaatatacagaatcggccattttcaatttgacaaacaattttatcggcaacatgcaatcctgcacgttgtacttggcaatcacgtccagccgtcgagtgttgtacatcttgaccatttcggtccaaggcaaatcgattttgttttcacccaaatagtaactactgattgtgttcaattgaaagttttcaactttatgctgattagaatcgctgctgaaaaatttatacaaatcaatgtgaatgtaatagttaaaataatacgtgtccactttgttgcccaacttgtttataaacagctttgtcgtcggcgccgcagccggcaaatcgtaacgctttaatagcattttggttttattcaatcgtccaagtatatagggcagatcaaatacgtctccgttaaaatccaaaatcacatcgggatttgtaatttttatcatgtcaaaaaacgctgtaatcatgtcgatttcattttgaaacatgaccacatacgtgtcatcgtcataggtctctggaatctgggtcggcagcttgtgatacataaaacaaaattttgcatactcgtcgtttttgtacaccacaaatcctatagacattatgcaatcaaccgatgctttcgacatgttgtggccgtccgaatgagtctcaatgtcatagcacgacaaaacgggcatgatgccgctggttaaagtcatttcatcgaccaactcaaagtcttcattaaaatgttgcaaattaaacatgcgcgtcgtcgatccaccgacatagttattttggcagcgttgtgttttcttgaatcgcatataggcgccttccacaaacggcgtttgcatgtgtacgcgattaacgttgtgaagaaacttgtccaaacacgccgcgttgtccgatggcgctgctttgtttctttcgtatttaatcacgtttatcttgttcaaataatttccttccacgcccggcgccacaaacgtggtgtagctgatgcacttgttgcggcaagacggaaatatgtgcttgtcgtagcattgtttgtaagaatacaaatttagttttactttaaagtaaaactgcagcactcgttctttgatatttgtattacaaaatgcaaacaagcaaccttgtttttcatcgtaatgcaaacgaatgatacgaaacgtatcggctgaagtaatattgaattctcctggttttgcatattctgcaaagcgcgttttgagttcattgtaaggatatattttcatttttaaatatgcagcgatggcccaaatatggaggcacagacgtcaacacgcgcactgtacacgatttgttaaacaccataaacaccatgagtgctcgaatcaaaactctggagcggtatgagcacgctttgcgagagattcacaaagtcgttgtaattttgaaaccgtccgcgaacacacatagctttgaacccgacgctctgccggcgttgattatgcaatttttatcggatttcgccggccgagatatcaacacgttgacgcacaacatcaactacaagtacgattacaattatccgccggcgcccgtgcccgcgatgcaaccaccgccaccgcctcctcaaccccccgcgccacctcaaccaccgtattacaacaattatccgtattatccgccgtatccgttttcgacaccgccgccaacacagccgccagaatcgaacgtcgcgggcgtcggcggctcgcaaagtttgaatcaaatcacgttgactaacgaggaggagtctgaactggcggctttatttaaaaacatgcaaacgaacatgacttgggaacttgttcaaaatttcgttgaagtgttaatcaggatcgtacgcgtgcacgtagtaaacaacgtgaccatgattaacgttatatcgtctataacttccgttcgaacattaattgattacaattttacagaatttattagatgcgtataccaaaaaacaaacatacgttttgcaatagatcagtatctgtgcactaacatagttacgtttatagatttttttactagagtcttttatttggtgatgcgaacaaattttcagttcaccacttttgaccaattgacccaatactctaacgaactttacacaagaattcaaacgagcatacttcaaagcgcggctcctctttctcctccgaccgtggaaacggtcaacagcgatatcgtcatttcaaatttgcaagaacaattaaaaagagaacgcgctttgatgcaacaaatcagcgagcaacatagaattgcaaacgaaagagtggaaactctgcaatcgcaatacgacgagttggatttaaagtataaagagatatttgaagacaaaagtgaattcgcacaacaaaaaagtgaaaacgtgcgaaaaattaaacaattagagagatccaacaaagaactcaacgacaccgtacagaaattgagagatgaaaatgccgaaagattgtctgaaatacaattgcaaaaaggcgatttggacgaatataaaaacatgaatcgccagttgaacgaggacatttataaactcaaaagaagaatagaatcgacatttgataaagattacgtcgaaaccttgaacgataaaattgaatcgttggaaaagcaattggatgataaacaaaatttaaaccgggaactaagaagcagcatttcaaaaatagacgaaactacacagaggtacaaacttgacgccaaagatattatggaactcaaacagtcggtatcgattaaagatcaagaaattgccatgaaaaacgctcaatatttagaattgagtgctatatatcaacaaactgtaaatgaattaactgcaactaaaaatgaattgtctcaagtcgcgacaaccaatcaaagtttatttgcagaaaatgaagaatctaaagtgcttttagaaggcacgttggcgtttatagatagcttttatcaaataattatgcagattgaaaaacctgattacgtgccgatttctaaaccacagcttacagcacaagaaagtatatatcaaacggattatatcaaagattggttgcaaaaattgaggtctaaactgtcaaacgccgacgttgccaatttgcaatcagtttccgaattgagtgatttaaaaagtcaaataatttctattgtaccacgaaatattgtaaatcgaattttaaaagaaaattataaagtaaaagtagaaaatgtcaatgcagaattactggaaagtgttgctgtcacaagtgctgtaagcgctttagtacagcaatatgaacgatcagaaaagcaaaacgttaaacttagacaagaattcgaaataaaattaaacgatttacaaagattattggagcaaaatcagactgattttgagtcaatatcagagtttatctcacgagatccggctttcaacagaaatttaaatgacgagcgattccaaaacttgaggcaacaatacgacgaaatgtctagtaaatattcagccttggaaacgactaaaattaaagagatggagtctattgcagatcaggctgtcaaatctgaaatgagtaaattaaacacacaactagatgaattaaactctttatttgttaaatataatcgtaaagctcaagacatatttgagtggaaaactagcatgcttaaaaggtacgaaacgttggcgcgaacaacagcggccagcgttcaaccaaacgtcgaatagaattacaaaaatttatattcattttcatcttcgtcatacttcaacagtcccaacacgttcatgttgtgattctcgccgttctcgacagttacgtaaatagttactttgattaaattatcttccagcagcattgagatttgattgaaatccgcacatagcttttgtagcgaatccgcttcggtttttttatttgtgttgacgtagaaaacagatttgttccatttgcccaagtcggaagaggtagaacagtcatccgaatcggcaatgttcaactcgtcgcttttaaactgcacaataaacttgttatcgcccatgtcattttcttccaattcgctttttaacacatttacattgtacgaagcaacgtgtttgttcgatcgactaatgttgatctttgcgtttgtgcaattttgcaaatttgaatatgcttcgctttctttagcctcgcacaattcgatgcgcgtagagttgaccacgttccaattcatgtacacgtttgatccattaaaaatttgttgacactttatactgtaaatggtaaagatttggttttcattgtcttttaaatatttaaacacctcattgatgtcgtcagacccctttatattgttcttgaatagatttattagtgttttcgcattgacagaacattccacttgaaccacgtcgggatcgtcgttgagatttttgtacacaacctcaaaaacaactttgtacaaaccgctgttgattttcttgtagataattttgtactttacaataatattgacgccatcttcattttcaaaatgtttgttagtcaaatagtcgctcatgggggttgcagtttcaatttccatttcacattctttgtattcgttgatctgaatcatttgactaaactttgttttcacataatttaaactaatgtcatagcacttgccttcttccatgtctttgaaagattgcgaatcgccgtagtattcttgaattttgttgtcggacattattcgaaaagtgtaatggtattcattatcgatactcaacgtcattttgctcatcaatttaccactaatccttttgtaattttctctaatcttcttggggctactggccatagccatgcgttttataagcggctcaccgctactttctccagacaaagatcttttggtcgccatattgctgttgtcgatatgtgggaatctatccgatggcaaatactgaatggcgacgaaatcgaagtgtcgccagagcaccgttcgttagcgtggagggagttgattataaacgtggccagcaacacgccgctcgacaacacgttcagaacaatgtttcaaaaagccgattttgaaaatttcgactacaacacgccgattgtgtacaatttaaaaacaaaaactttaacaatgtacaacgagagaataagagcggctctgaacagacccgtccgatttaacgatcaaacggtcaatgttaatattgcgtacgtatttttgttctttatttgtatagttttgctgagcgtgttggccgtctttttcgacacaaacattgcgaccgacacgaagagtaaaaatgttgcagcaaaaattaaataaactcaaagatggtttgaacacgttcagcagcaagtcggtggtttgcgctcgctcaaaattatttgacaaacgcccaacgcgcagacctagatgttggcgaaaactatcagagatcgacaaaaagtttcacgtttgccgacacgttgacacgtttttggatttgtgcggcggaccgggcgagtttgccaactataccatgtcgttgaacccgctttgcaaagcgtatggcgtcacgttgacaaacaactcggtgtgcgtgtacaaaccgacagtgcgcaaacgcaaaaatttcacaaccattacggggcccgacaagtcaggcgacgtgtttgataaaaatgttgtatttgagattagcatcaagtgtggcaacgcgtgcgatctggtgttggcagatggctcggttgacgttaatggacgcgaaaacgaacaagaacgtctcaactttgatttgatcatgtgcgagacgcagctaattttaatttgcctgcgtcccggcggcaattgcgttttaaaagttttcgacgcgtttgaacacgaaacgatccaaatgctaaacaagtttgttaaccatttcgaaaaatgggttttatacaaaccgccttcttctcggcctgccaattccgaacgctatttaatttgtttcaataaattagttagaccgtattgtaacaattatgtcaacgagttggaaaaacagtttgaaaaatattatcgcatacaattaaaaaacttaaacaagttgataaacttgttgaaaatataacgtgtgtataaaaagccagcggcttcaaatcaggcatcattcaacatggattcgctagccaatttgtgcttgaaaaccctgccttacaagtttgagccgcctaagtttttacgaacaaaatattgcgacgcatgtcgctacagatttttaccaaaattttctgatgaaaaattttgtggacaatgcatatgcaacatatgcaacaatccaaaaaatatagattgtccatcatcatatatatcgaaaattaaaccgaagaaagaaaacaaagaaatatatattaccagcaacaagtttaataaaacgtgcaaaaacgaatgtaatcaacaatcaaaccggagatgtttaatttcctattttacaaatgaaagttgtaaagagctcaattgttgttggtttaataaaaactgttacatgtgtttggaatataaaaagaatttatacaatgtaaatttgtatacgattgatggtcattgtccttcgtttaaagccgtttgtttttcatgtataaaaagaatcaaaacgtgccaagtttgcaatcaacctttattgaaaatgtacaaagagaagcaagaagagcgtttgaagatgcagtcgctgtacgcaacgttggccgatgtagatttaaaaatattagacatttacgatgtcgacaattattctagaaaaatgatattgtgtgctcaatgtcatatatttgcacgctgtttttgtaccaataccatgcaatgtttttgtcctcgacagggttataagtgtgaatgtatatgccgacgatctaaatattttaaaaataatgtattgtgtgttaaaagtaaagcggcttgttttaataaaatgaaaataaaacgtgttccaaaatggaagcatagtgtagattatactttcaaaagtatatacaagttaataaatgtttaattttaaggatattgttatggaataaactataaaatgaatttgatgcaatttaattttttgatactttccacagacggtagattcagaacgatggcaaacatgtcgctagacaatgagtacaaacttgaattggccaaaacggggctgttttctcacaataacctgattaaatgtataggctgtcgcacgattttggacaagattaacgccaagcaaattaaacgacacacgtattcgaattattgcatatcgtcaaccaacgcgttgatgttcaatgaatcgatgagaaaaaaatcatttacgagttttaaaagctctcggcgtcagtttgcatcacaatccgtggtcgttgacatgttggctcgtcgcggcttctattattttggcaaagccggccatttgcgttgttccggatgccatatagtttttaaatataaaagcgtagacgacgcccaacgccggcacaaacaaaattgcaagtttctcaacgcaatagaagactattccgtcaatgaacaatttggcaaactcgatgttgcggaaaaagaaatactggctgccgatttgattcctccgcggctaagcgttaaaccttcggcgccgcccgccgaaccgctaactcaacaggtctccgaatgcaaagtttgttttgatagagaaaaatcggtgtgtttcatgccgtgccgtcacctggctgtgtgcacggaatgttcgcgtcggtgcaagcgttgttgtgtgtgcaacgcaaaaattatgcagcgcatcgaaacattacctcagtaaacattgcaaacgactacgacattctttaaaaataagctatatataaatattgcattgtatgacaaaaaaattattaacctactgcaaagtaaaacttgtaaaaggcttttcaaaaaaatttgcgagtttattttgtcgctgcgtcgtgtcgcatctaagcgacgaagacgacagcgacggtgatcgctattatcagtataataacaattgtaatttcatatacataaatattgtaaaataaaagacatattattgtacataatgttttattgtaattaaattaatacaccaatttaaacacatgttgatgttgttgtgaataatttttaaatttttacttttttcgtcaaacactatggcgttgctttcgattagttttttcgttagcatttcatctaaaaaatcaaactgtttgcccggcgcgtttagggattctatggtgtagtcgggcgtgtcgctgtttagatattggtccacttcgcgcattatgtccaagacgttgttctgcaaatgaatgagctttgtcaccacgtccacggacgtgttcatgtttcttttttgaaaactaaattgcaacaattgtacgtgtccactatacaattcggcttaatatactcgtcggcgcaatcgtatttgcaatccaattttgtgttcaacaaattggtgatgatatctttgaacgtgcacgttttcaatttgtccttatcggccaacgcaagtttcaattcgctctgtaaagtttctaaaattttgtctttattgttgtcaaattcgtgcgtgttgcgttccaaccacaatttgaacggctcgtcgacaaaaatgctgcgcaacacctcgtacaactgtctgcctaacgtgtacacttgctcgtattctttcatgctgacctctttgctaacgtacattactaaaaaatctacaagtattttcaaacatttgtaataggcgacgtattttgatttaagttttaaaccgtccaccgtgtattcgtccacgttcgcatcgaccacttttcgattattatcgccgcttgttgccggcgcgtcggcctgttcggttttaactatatccggttcaatatttaaagtttcaaaagatttaatggcattcataaaatcatctttttgctttggcgtggtcaatggtaaatctatcgaggagttgtcgtccgtgtgctcttcgggcacgctgttcagacgtaacgtaatctttttgggatcgtcttcatcgggtatcaaatcggctttaattttattagaattgagcaacgacatggtggtcgcttgtaaatttaataaattaattaaagactgaaattgtatattgcacaaatttattttcatttttattgatcttactattaatacgctggcagttggtatgcttcatccatttttgtgactagaaaatttgctaaaaaactgagctcgtcctgtgttaaaacgttgtcgtccacgaatctatgcaatgtaaatgttacactgacattgtttaacaatgcatgtattaaaaaatcaacctgtcgcctactgagtttattagaagagtcgaccgtttctactagtttgtagattttgttattttcaatttcattgtttaaaaacatgttaactactcgtttgagtttaagcgaaaaatccttgtccggatagacttgttcgcacagccaattgctaagagtggttttgaccacggacaccttggtggtgaacgtcgtcgatttgaccagttcggtgaaaaagtttttcattaaattggacattttaacaaacacttatcaatctattgagctggtatttttgtttagaatcgcatcaagcgcttgctcgatctccaatttttttcggacgctcttagctttatgactcggtatgtcttctacggtagactcggtgttcttacttataatggccgggctgacgataataaacacgagaaacaatatgagcagatacaaaaagatgctgttttcctttttgtcatacactaggctaaatatggccagtgcgcccaacaacaaatataaattcatttttattcccttactctattcgttgcgatagtacaacaacgattctcccgacgaaccggacgaattgcgattatgctgcgcgtcgtcgtcgtcgttgttgttctcctcttcgctgctcgtttcgtctaaacctatattgtatttgttcaagtaatgtttggtgcttgcggaggattcgtggttcattaatttggccactttttgtaaaggcacgccgctattgtataggttactgctcaaataatgtcttatcatgttgctgcgcggccgttccatctcgacgcccgactcttcaaggagtcgcctgaaatctttgaagggcgtcgaggtgtttttagatatttgcaaaatggtcgggtttcgtgaataaatctcgcgtgccaattccaacggtttcattttgatgttgttgagtgtgttattacgactgcgttttcgctttaaattaatcgtgtcgctgtgcagttttcctcttttaattagcacgttgagatcgtccacgctgagttggcgcgcttcgttgattcgcatacccgtccctaacatgatgcaaaacactatcgcgcccctaattagaccgcggtcgtgaacataatcgctgttgagcattttaattttatcattaataaaatttaatatggtatctattacgtttttaagcattaaattcttttccttttccctgatatttttgagctccttgtcgcgcggcagcataaccatgcggggaattttgtattcgggcaagttcatcatgttggtgtaaaagtttatagtcaactgtagtgtttctttggtgaccgagcgaagttcgagcatgcgcctgcacagttcttggggatcaatgagaagtgtttggttttctatcgagtcaaactccttgtccaacgagtacgacatgtcttccaggtgaacatcgtctaccgagcagtacacaattttaatgaatcgagacttgtaactttttaaagtggtgggcgcaaacggtttggggaacatgtacttgctccacagactgttgtttttcacctcgtcgggcgtgcatcgttgccgatcggtggccaaatcgaacacggactcgaaccggggagcggattgaatttttattttccaagaattaaaattgttttcgttgcgaacattaaaaccgttcattgtggttaatcaaatttattaaaaacaaaaggagaatcggtgtcaatactatccgaatattgttgttgttctcttaatattacgaaataatatattacatacagcagtaagaataaagctataaaagcgactacactaattaaaattataattcccgccgacacgttgctcgtcgtgttgtcatagcccaccatgtcgtttattggcattttgtgaacgggctcgctaaattgttgcggttcgctggcagtatcgtcgttgagcgccaatttcaacgggatgtattccaccttttcgtggttgcccaaccgatagtagggcacgtccaaattcatgtttacaacttatttgctaacaggaatttatgcaacaaaagtggtttggctttgatgagacgcaatttgaaatacttgctgcatttacgcttaagattgtattccatgcgggcggcggtcttgtagtcgtacgcgctcgcgctgtgatacacgagccgtaaattggttgcgttgcgcaaacacttggcgccttgtttgttcgaatgctgttttatgcgtctgttaagattgctcgtgatgcccgtgtacaattttccattgtcttgccgcagaatgtacacgcaccacaccttgttggtgtacagagtcgtcgccatgattatgcagtgcgccctttcgtgttcggccgagtggcgttaggcgcagccgcggcaataatcgcgttggcgtccttgttgtaatttatttgttgaaaaataaaacgtcttagagtttcgttttggaacgccaattcggtcaagctctcctggcaagcgcttttggtcaaatgagcggccggcgaattgaccgcgttggcggccgacgttaagaaggtggcgttctggaacatgctgggctgcttgccggctcgcgtcgccagctcggccatgtaattgaatatgttggcagacgcagatagcggcgccaaaaacgcaacgttctcttttaaactcatgactcgcgccctgtttttttcgttcagcacgtagtggtagtaatcgccgccgccggcaaacagatcgtcaatcacggcgttgatcagatcgttgatcatgttgatgtgcggaaagcgacgcgactcgactgcgctctgtatgtttggcggcagagtggcgtgcttgagcaacagagtcatgtaattgttggccagctgctgattgaaaggtaacggaatgggaatgttgcacgtcaccgcttccgccaccatgtactggacggccagactgagttgtttggcggcctcggccaaagcgtctttgcccaacatatcagcgccaccgttgtaaaacttttgcgcgtacgccggcagcgaatttagcacaaacgatggctgaaatatatttgaatcgctcgacagggactcggccgcgttgctctgtcccaactctttttgcaaccgaatcaggtggcgtatcatggtttcctccgattcaaaccgctttaccacgtttacgctgattgggttcgtgtcgatgcacatgtcacgaatagtgtttataaaaagaatcatgagaggactaagttctgacatgtcattgcacctgtaatatctaataatcttttgaacaaaatccacacatttgttgtaccaaatagattcaccggcgtcgagcgtcggttctttgctcttgttgtacggtgcaatcgctaccgagtttgtgctgttgctgcggctcgtgtaatccatcctgttgtcgcgcgtggcgacggtcgtaggcaccgtcgccggcggcacgtacccgggcgcgttgtaagtttgcgcgctggtgaatatggccgttgccggattagagggatacctcagcggcggaggggtgttgtaataaaaattgccacgttcatctgtcatactttttatttgtactcttatgattacaaaactcaatatacggattacttataatatagttgttgtgacaaaaaagcgataataaaattaacaaaattatcaacaagttaatcatggaaaatttttcaacgttgaataacaacaacaaaatggcgcaggtcaacagcaccgtttgaaaactgacgcgccgacacaaaatgctttcgcaatttctaaaagccacattaaacgaattttcacctttgatataatcacgcagttcttttttacaacattcgtcgcacaaaattaacacctttataatgaggccgtcggtgtgtatcgtttgaaatgtccgcggttgactgcctggatgaaattcaaacgagtacccagtggacacgtgtatctgtgcaaaataatgggctaatatcgaggcgcccgtttttttaacctttacttttgatattttaataacattaatgttgttatttgcgtaatcagagtttttattgtggtgatcatcgtacaaataatgaagcaacagttcactatcgtatttaatcttgtttagcgttgtcaagtttttgtttcttaggcgttggagcgtctccgtcgtcgatattttcttcgaaatcgagtccaacaacgtcggcgtttccttcttgctcatcgatagcggcggcggaggcggcctctccgtcgtcgtcattctcggtttctacagtgcgtttgggcgacgacgtgtgtacagcagcgtccgtcttactattatcggaccgccaaatttttgtttgaaataacatttggcccttgttcaactttatttcggcgcagttaaacattattgcattaagatcatattcgccgttttgcaccaaattgcacaaaacaccatagttgccgcacgacactgtagaataggcgtttttgtacaacaatctgagttgcggcgagctagccaccttgataatatgggcgccaacgccccgtttttttaagtaatattcgtcttcaattataaaatctagtacgttttcatcttcactgttgatttgggcgttcacgatgatgtctggcgtaatgttgctcatgcttgccatttttcttataatagcgtttactttaatgtatttggcaatttattttgaatttgacgaaacgactttcaccaagcggctccaagtgatgactgaatatgtgaagcgcaccaacgcagacgaacccacacccgacgtaataggctacgtgtcggatattatgcaaaacacttatattgtaacgtggttcaacaccgtcgacctttccacctatcacgaaagcgtgcatgatgaccggattgaaatttttgatttcttaaatcaaaaatttcaacctgttgatcgaatcgtacacgatcgcgttagagcaaatgatgaaaatcccaacgagtttattttgagcggcgacaaggccgacgtgaccatgaaatgccccgcatattttaactttgattacgcacaactaaaatgtgttcccgtgccgccgtgcgacaacaagtctgccggtctttatcccatggacgagcgtttgctggacacgttggtgttgaaccaacacttggacaaagattattctaccaacgcgcacttgtatcatcccacgttctatcttaggtgttttgcaaacggagcgcacgcagtcgaagaatgtccagataattacacgtttgacgcggaaaccggccagtgtaaagttaacgaattgtgtgaaaacaggccagacggctatatactatcatactttccctccaatttgctcgtcaaccagtttatgcagtgcgtaaatgggcgccacgtggtgggcgaatgccccgcgaataaaatatttgatcgcaacttaatgtcgtgcgtggaagcgcatccgtgcgcgtttaacggcgccggacacacgtacataacggccgatatcggcgacacgcaatatttcaaatgtttgaataataacgagtcacaactgataacgtgcatcaaccggatcagaaactctgacaaccagtacgagtgttccggcgactccagatgcatagatttacccaacggtacgggccaacatgtattcaaacacgttgacgacgatatttcgtacaacagtggccaattggtgtgcgataattttgaagttatttccgacatcgaatgtgatcaatcaaacgtgtttgaaaacgcgttgtttatggacaaatttagattaaacatgcaattcccaactgaggtgtttgacggcaccgcgtgcgtgccagccaccgcggacaatgtcaactttttacgttccacgtttgccattgaaaatattccaaaccattatggcatcgacatgcaaacctccatgttgggcacgaccgaaatggttaaacagttggtttccaaagatttgtcgttaaacaacgacgccatctttgctcaatggcttttgtatgcgagagacaaagacaccatcgggcttaacccgttcaccggcgagcctatcgactgttttggagacaacttgtacgatgtgtttgacgctagacgcgcaaacatttgtaacgattcgggaacgagcgttttaaaaacgctcaattttggcgatggcgagtttttaaacgtattgagcagcacgctgaccggaaaagatgaggattatcgccaattttgtgctatatcctacgaaaacggccaaaaaatcgtagaaaacgaacattttcagcgacgtatattgacaaatatactacagtcggacgtttgtgccgacctatatactacactttaccaaaaatatactacactaaactctaaatatactacaactccacttcaatataaccacactctcgtaaaacggcccaaaaatatcgaaatatatggggcaaatacacgtttaaaaaacgctacgattccaaaaaacgctgcaactattccgcccgtgtttaatccctttgaaaaccagccaaataacaggcaaaacgattctattctacccctgtttaacccttttcaaacgaccgacgccgtatggtacagcgaaccaggtggcgacgacgaccattgggtagtggcgccgccaaccgcaccacctccaccgcccgagccagaaccagagccagaacccgagccagaacccgagccagagttaccgtcaccgctaatattagacaacaaagatttattttattcatgccactactcggttccgtttttcaagctaaccagttgtcatgcggaaaatgacgtcattattgatgctttaaacgagttacgcaacaacgttaaagtggacgctgattgcgaattggccaaagacctatcgcacgttttgaacgcgtacgcttatgtgggcaatgggattggttgtagatccgcgtacgacggagatgcgatagtggtaaaaaaagaagccgtgcctagtcacgtgtacgccaacctgaacacgcaatccaacgacggcgtcaaatacaaccgttggttgcacgtcaaaaacggccaatacatggcgtgtcccgaagaattgtacgataacaacgaatttaaatgtaacatagaatcggataaattatactatttggataatttacaagaagattccattgtataaacattttatgtcgaaaacaaatgacatcattccggatcatgatttacgcgtagaattctacttgtaaagcaagttaaaataagccgtgtgcaaaaatgacatcagacaaatgacatcatctacctatcatgatcatgttaataatcatgttttaaaatgacatcagcttatgactaataattgatcgtgcgttacaagtagaattctactcgtaaagcgagtttagttttgaaaaacaaatgagtcatcattaaacatgttaataatcgtgtataaaggatgacatcatccactaatcgtgcgttacaagtagaattctactcgtaaagcgagttcggttttgaaaaacaaatgacatcatttcttgattgtgttttacacgtagaattctactcgtaaagtatgttcagtttaaaaaacaaatgacatcattttacagatgacatcatttcttgattatgttttacaagtagaattctactcgtaaagcaagtttagttttaaaaaacaaatgacatcatctcttgattatgttttacaagtagaattctactcgtaaagcgagtttagttttgaaaaacaaatgacatcatctcttgattatgttttacaagtagaattctactcgtaaagcgagtttagttttgaaaaacaaatgacatcatcccttgatcatgcgttacaagtagaattctactcgtaaagcgagttgaattttgattacaaatattttgtttatgatagcaagtataaataaccgaacaaagttaaatttttttcatttacttgtcaccatgtttcgaatataccctaataacacaactgtgcccggttgtttagtgggtgacattattcaagttcgttataaagatgtatcacatattcgctttttgtcagattatttatctttgatgcctaacgttgcgattgtaaacgaatatggacctaacaaccagttagtaataaaacgcaaaaacaaatcgctgaaaagcttgcaagatttgtgtctggacaaaatagccgtttcgctcaagaaaccttttcgtcagttaaaatcgttaaatgctgtttgtttgatgcgagacattatattttcgctgggtttaccaattatttttaatccggctttgctacaaagaaaagtgccgcagcgcagcgtgggatatttcatgaattcaaaattggaaaggtttgccaattgtgatcggggtcatgtcgttgaagagaaacaattgcagagtaatttgtatatagattatttttgtatgatttgtggtttaaatgtttttaaaataaaagaataacaatttacacattgttttattacatggataatgttgtttgtttgacattaaaggttatcatggtgcaatgattaataataaaacaatattatgacattattttcctgttattttacaatataaaatcacaccaattgtgcaaagttttattatttgtttgtcgacggtcgaggggtcagcggcgtgtgcaacaataaaaaacatgaagctgttaacaattttgattttattttattcattttttatgaatttgcaagcgctaccagattaccatcaagcaaataggtgtgtgttgctgggaactcgcattggatggaacgatgacaatagccaagatcccaacgtatattggaaatggtgttaaataaaagtgaatatattttttataaaattttttatttaaaattccaagtaatccctgcaaacattaaacactgtaggtatttttaaatcttgccacatgcgaacaacgcacggcctgtcgtcgaacaccgctattacattatattttcctctgatatagttgttaaacaattttaattttaataaataatctttacaagtatcgtctgaaggcctcataaacaatttatatgatttaatatcaaaatacttttcaatccagtttcgagtgggctgttcacaaattacgcttctcccgctcataaacacgataattgcgtcgtggcaatttgccaaatacttaacgcaagtaataacgtctaagcgggcttcatcttgagcaactctattatcaaaatcataaaacgatctatttgtgggcaaagctactgtaccgtctaaatcacataatacagcgcggggaaatttgtcgccgacaggaacgtaatattcgaaattatttacctttagaaactttttatattgctttttaatagtttctggatttaatggaaatttatcagagcgtttataattgcgttcaagagccgtttccaaagaaacgtccatcaaacgcgttaaaaaatggtaattatgcgttgcggccattttttgccacatgtccaccgattgagtgttcaaattagtgtcgctgacaaccacgttggcaccacattttgcggcttttaaaaactgttcaatgcacattttggtaatttgttcttctttagtttgtctacatttccgcgattggttatagaaagcgttcagttttgtataatcgccgtttaaaaacaacttaacgcgcacgtcgtctctgttgatttctgtatagccttttaaacttttggcatacgtgcttttgcccgaacccgaaatgcctatcaacaccaacaattgttttgaagaaggcaatttaattgttggagcaagtttattatttaatgcctgcttagtcgatacaaattttataatatttttgatcattttaattttttcaggctcggttaattttaaaaattcgctctccacatcgattgtttgtgctttacgacatctgtacgctaaacatttccacggcaaagtttgcaccagttcgttgaaacgctgttgattcaaagtcaaacccgacaccataatatttattgtagactcgttggtgaacgtgtttctagcatcaacgtacggtttaatgacactttttaaatgcgggaaaagagctagaaagtcatcgtgttcgccatttataacaagctgcgccaatttagtaggattttcagcacggctctgatttttgtgcatgttcaaatacacgtcgcttttaatcttgcatagtggcgcgttgtttttatcgtaaactacaaatccttcttccaaatttttcaactgggccgcgtgttcgacacattcttgcacagacgtaaactcgtaacatttggggtatttgcaaaacggcaaattggaacagtaaaaataatcgcccgtttcgttgtttctgcttgccaaataccacaacgttggctgttcatcgtaaacggttacaattctgttgtgtttgcttgttaactcaaacatgtgagtcgacgcgcagtctaaatattcgttacacaacgcttgaaattgattgtgggcctcgtcaagttgaagagcttgcaaaactaaacgtttaaacgtcacgtctgacacgcaaaggttttctgcaaaagcacttcctcgggtgctggcatgccattcgccgttgtacttgtagattttaattaaacttccgtcgattttttcgtaaaacttaaaattctccttcgattggaacagtttgtgatgagcatcttcgccgccgatattttgtagcaattcttgaaaattaaagaaacgatcgaaagaacgcgacacaacggcgtacgtgcggctgttaagaattaaaccgcgacattccacgaccacaggatgatctcgatcgcgttcaaacgattcgtaattaagaaccatcaaatcgtgttcggtataatttttaattttgactttaaacttgtcacaaagatttttcactccgccgtttgcaagtagacgcgaaacgtgcaacatgattgctgtttaataatgcataccaatgctaaactgtctattatataaagtgcagtgataactttgttatcaacgcgttcgatgccgacatatataaacgcaatgtaacagtttttgctagtaccatcgcatacaacattatgaatacaaggggttgtgttaataataataaaatgatatttatgaatgctttgggcttgcaacctcaaagtaaattgaaaattattgcacataaaatactagaaaaatgtaaacgtgacgcgtacacgcgtttcaagggcgtaaaggcgatcaagaatgaactaaaaacatacaatcttacgttgcaacaatacaacgaggcgctcaatcagtgcgctttaaacgatagccgatggcgcgacacaaataattggcatcacgatattgaagaaggtgtgaaaataaacaagagacatatatatagagttaattttaattctaaaacccaagaaattgaagaatattattacattaaagtagaatgttatgtaaacagttaattaatctacatttattgtaacatttgtggtaatagtggcgttggttatacatttatatgattgtaatgttgtgtactcgttttgtaataaatttttgtgtttaatcaattcaatatttttatttgataaaaccttattttcgctactcaatttggcgtttttagacgcaagttttgcgtaatcgtcattgagcgattttagcgccttttcagttgtaattcgtttcagttgcaattctttaaaagatttatgcatgttgttgtagtcgcttttaattttgtctaacttttcttgcatagaaacgcttgtttgttgtaatttgtctaaatctaattgttgtttaatgttgagctgcgtttgttcggcaatgtctacctgtagtttttttagtatcgcttgtgcttcagacagcatagtgtcgtcggcatttgcgttgttgtcttctgcgtcgtccaacagacttttttcaaacaacacactggccaaagaggccgcatcaaaattagcgtttattttattccattgtgcgacactcgacgcgctgcatttaatcacatccacaacgtttcggtttacgctgtaaacgttgaaatgcaaactttcaaccctacacaagggacatggtactttttttcgttttctaatcttgcgtatacacattgagcataattgatgtttgcacgtgtctagttctaatacgggtattatagtcaatctgtctattggttgcagaaaataatttttaatttctgcaaccgaaaaacaaatgttgcattgcaatttaacaaactccatttttagacggctattcctccacctgcttcgcctgcaacaccaggcgcaggacctgccactgcgccgccgcccagagtagcgttaggatttgctcttggtataaagtcgttgcgcaaaaagttgttttctgaattgattatttggtatcccaaaaacagcggaacgtacgtcgggtattcttcgtatccgctaagcgttctgtccagctcacgtgtgtcgccttcaaatttcaaaacgtttctaatttgcaaacgattgggttgacttctcataatgtcactgcttcttatcgggttgtacaactcggggccgtcgggcacagacgcgaccagacccgtttcgtcaattatacacgtggcgcaatttctaaacctcaattcctccgtgtcgatttgcaagtactcgggcgctactgcgcgtcgaatcaaattttgcaaaaatccactgtaattgttaaataattgatcgccagcaccgcctcgaagcgctcgggcgttggtcacgtcaaagaaacgcaattcgtctcgcgacacccgcgaacaaaacgtgttcgggtttgtggtgtccagaatgctttttgtagttgcgtaaacgctgtgtataacgcgttgcgtgttgcttgtgaaaccttcggtatattttagattgtcgcatatagtgttaactgcgttttcgttgttatatatcaaatgaaagattagctgttcggcttgcatcatactgtttagattaaacacgtcttggtaattggttgcgcttggaattaaaattcgcttgatacctctttctttatttccaactaaatgcctagcgatcgtcattttgaattgattgtcgtcttcgtcgaaaatgggcaaaaccatttttgacattttaaaacgttttatgaggtggttgttgcaaataaaccatccatcgtcatgatacgcgtcgggcgaacacggcgatttgtatgttatgcacgcgtcgaacgacacgatggacgcgaaaatgcagcgattaactctcatttgtcgcggcgccatacccacgggcactagcgccatattgttgccgttataaatatggactacggcgattttgtgattgagaaagaaatctcttattcaataaattttagccaagatttgttgtataaaattttaaattcttatattgttcctaattattcgctggcacaacaatatttcgatttgtacgacgaaaacggctttcgcactcgtatacctattcagagcgcttgcaataacataatatcaagcgtgaaaaagactaattccaaacacaaaaaatttgtttattggcctaaagataccaacgcgttggtgccgttggtgtggagagaaagcaaagaaatcaaactgccttacaagactctttcgcacaacttgagtaaaataattaaagtgtacgtttaccaacacgataaaattgaaatcaaatttgaacatgtatatttttcgaaaagtgacattgatctatttgattccacgatggcgaacaagatatccaaactgctgactttgttggaaaatggggacgcttcagagacgctgcaaaactcgcaagtgggcagcgatgaaattttggcccgcatacgtctcgaatatgaatttgacgacgacgcgcccgacgacgcgcagctaaacgtgatgtgcaacataattgcggacatggaagcgttaaccgacgcgcaaaacatatcaccgttcgtgccgttgaccacgttgattgacaagatggcccctcgaaaatttgaacgggaacaaaaaatagtgtacggcgacgacgcgttcgacaacgcgtccgtaaaaaaatgggcgctcaaattggacggtatgcggggcagaggtctgtttatgcgcaatttttgcattattcaaaccgacgatatgcaattctacaaaaccaaaatggccaatctgtttgcgctaaacaacattgtggcctttcaatgcgaggttatggacaaacaaaagatttacattacagatttgctgcaagtgtttaaatacaaatacaacaatcgaacacagtacgaatgcggcgtgaacgcgtcatacgctatagatccggtgacggccatcgaatgtataaactacatgaacaacaacgtgcaaagcgtcacgttgaccgacacttgccccgcaattgaattgcggtttcagcaattttttgatccaccgctacagcagagcaattacatgaccgtgtccgtggacgggtatgtcgtgctcgacaccgagttgagatacgtcaaatataaatggatgccaacaaccgagttagagtatgacgccgtgaataagtcgtttaacacactcaatgggccattgaacggtctcatgattttaaccgacttgccggagttactgcacgaaaacatttacgaatgtgtaatcacggacacgacaataaacgtgttgaaacatcgtcgcgaccgaatcgtgccaaattaaagcacgttaagcggatacaacgggcagtccgagctgttaaagtcaatacaaccatcgttaacaaacgaatacgcattgttgtgacagctgaggatataaaaaggaatagagaagtaattgcaatgaaatatcccgttacaattccacggcacagcgtatgttgctcgagttctatcagttgcacacaacggcctaagaaaatttattaatgcttcatttgtatctatattagaaggataatacataggttcgcccaaaggactgggagaaggcggcggcgaaggtgtaggtgtaggaggaataggagaaggcggcggcgaaggtgtaggtgttggaggaataggagaaggcggcggcgaaggtgtaggtgtaggaggaataggagaaggtggaggtgtaggtgtaggtgttggaggtataggtgttggaggaggtgtaggtgtaggtgttggaggtataggtgttggaggaggtgtaggcgaaggtggagaaggtgtaggagtaggtggaggtgtaggtaacggtacaattggtggagatgtaggtggtggtacaattggtggatttggatacaattcctgaatgtcgtctaatatttttaaagttaataaaattattataaataaatttaatattattattattattattatcacaataatgtaccacatgttgcttaaatataaaaattaaacaaagaatgttgtattattgcaaatttaacaattttttgtattctccccatgtcatgcgttcgtaatgagcgggcggttttttatttctttgtatccacttgtaatcgttaatgtggttgtgaaaagtcatactgacgtaggccattaaatttttcatgagcatattatttgacacaactgcaacatctgcgcctgccgtttcttgctggtacgaatcgacaaacgtaatgtctgtgccgtatttttctttgtcaagtgcaatttctataagctcaatgtggtaaatgatgaaacctttgacgttcatataatgatcgcggcacatggcgcactgtagtatgaaaaatacgttgtaaaatagcaccttcattgttttcaactgctgcatgacaaaatctaaactgcttttgtctcgcgtatacaccatatcgtcgatgatgagactgagaaagtgcatggtgtcccatatggtagtaaacgtgtaagtaaaactcttgggctggcacgaacgcaaattgagttctgtggttttgtccataaattctatgcgaaactgttgcaagtccatgtcgggggatgcgttaatggcccattcgatcaactgctgcacctcgtacttttgaatgtctttgtatttcatcaaacacgcaaaatggtataagtaagttgcttgcgaagacaacagtttggtgaggtgcgtcgatttagaggctcgcaaaaggtctatgagacgaaacgaatacaacagatagctgtctttgtaacgagaaaaaagcggcgtcagcggtatcatggcgactagcaaaacgatcgtgctgtacttgtgtcaggcgccggccacagcgtcgttgtacgttagcgcagacacggacgccgacgagcctattatttatttcgaaaatattacagaatgtcttacggacgaccaatgcgacaagtttacttattttgctgaactcaaacaggagcaagccttatttatgaaaaaagtatacaaacacttggtgcttaaaaacgagggtgcttttaacaaacaccacgtattgttcgatgcaatgattatgtataagacatatgtgcatttggtcgacgagtctgcgttcggaagcaacgttatcaactattgcgaacagtttatcacggccatttttgaaatttttacgctcagcagtaaaatcgtcgtggccgtgcccgtcaattgggaaaacgataatttaagtgtacttttgaaacatttgcacaacctaaatctcattggaattgaaattgtaaattaaaacaaatcatgtggggaatcgtgttacttatcgttttgctcatactgttttatctttattggacgaatgcattaaatttcaattccttaaccgagtcgtcgcccagtttagggcagagcagcgactcggtggaattagacgagaacaaacaattaaacgtaaagctgaataacggccgggtggccaacttgcgcatcgcacacggcgataataaattgagccaagtgtatattgccgaaaaaccgctatctatagacgacatagtcaaagagggctccaacaaggtgggcactaacagcgtttttctgggcaccgtatacgactatggaatcaaatcaccaaacgcggccagcacatctagtaatgtaaccatgacgcgcggcgccgcaaactttgatatcaaggaattcaagtccatgtttatcgtattcaagggtgtgacgcccactaaaactgtagaggacaatggcatgttgcgattcgaagtcgacaacatgattgtgtgtttgatcgaccccaacacggcgccgctgtccgaacgagaggtgcgcgaattgcgcaaatctaactgcactctggtgtacacgaaaaacgcggcagctcagcaagttttattggaaaataactttaccgtcattaatgctgaacaaaccgcctatctcaaaaactataaatcatacagagaaatgaattaataaaacaaaaagtctatttatataatatattatttattaacatacaaaatttggtacactagtgttcaaatcgtttctgttcaacgccattgtcatgttataaaacacatttgtagttttattgtaattatttttaaatttatttttaatttgctgtaataaaacttgttcattaaatacaaaagactttgaactacttgcgtttatattttttttataattgtactgaacaaacgaagggtgcaaaaagttttttaaatgctgaacggcaatacctatcatttcctccattttgtcctctcctattgtaatagtagcactgcgcaccgttttaatgtttagaatgtaaatgagcgcatacagcggactattgttggtgcttaaacacattagattgtgcttatgcatagggtcgttgctcagcagcgttttgtatactacaaagcccgttttgggatcgcgtctgtacattaatacgtgcgacaaaaacaaacgcactggcgtcacaagcgattcgtaatacatgctttctattggaaactgtttcgacttgatgtgttcgtacacggagccggcaaacttgacgctgtctacaaacttatggttcgtgtaaacaatcaaaaatctgtcttgtacaccgtcgtcataatcgtccacgtacagcggcttgttgttaacaattaacattttgtagttggcttcatactttagcagcccttggtattttctgctcttggaatcgctcttgctcgaatcggcatgcttcttaaagtacgactcgctgcattgtttcaactcgttgatagtgtacaactgcgagttgagtttgctcacttccttgtcgctcgtttccttgttggactctccgctgtggttgtcatcgtcaaacttgtgcatcaacaccaaatagtccaacagctcaaaaaacgacgacttgcccgaacccggttcgccgggcatgtaaatagccttctttccgtaatctacgggaatggccaaactagcggcgaaatgcatcaacataatcgcgttcgcgtgattaaaattggtgaagcgtttaaagtacaaatagccttcgacaatctttttcaaataattgtacgagtactccttcaagtccactttggacatgatgatgcgcatgtagaatcgagtcagccaagtgggcaaatcgtccgtgctgcgcgccaatatgattttgtcccaccacacattgtacttcttcaagatcattaacgcgtcggcgtggtgcgtgtaaaatttggaaatgttatccgattcttcaaactgaacatcgggttcacgtgcaacatcatcgcgcaattcggttaaaaacaaacgtttatcattaaacttgtccatcaacatgtcgacatattcgattttgtgaattgttcgatacaagtactgaataattttgttgtgttctttggaaaaaaactctccgtgttggttaacaaattcgctgttcgtgcgaatcaacgtggtcgacacgtacgttttgttagtaaaaattagcatccaaatcaattcgctcaattctgcatcgttaccgaacatgtccgccatcaagcagacttttagcgcttttctattgatctttattttcttgtagcatttgcattttggtcgagatcccgataccgttgaccgacacggtttgcattttaggttgtgcaacatgtcggaaaccctgttcttgtttacgtacagagcgagcgtaatcagattttcatcgtccaaattccacaaatcgcgaaacaggttgtttaacgcgactcgcatatcggcttggcatgtgttgcaattgcccatgtagttaactatggccgtgttagtttttagcatttttacatctcggcacattttggcgatgtgataagttctataaatgctgagctcgtcggcgctagtagatagcatgtaattaaacgcgtcctcgggcaaatacttttcgtcggtgggcttcttgaatgtctgcggcaacgtggtgcccaacaaaaatggacagctcgaatgaaagctgttggtgaacacgttgtacacaccgtgcgttgtcaagtacaagtatttccaattgttaaattttatgttgctcaacttgtaacaattgcttttggtcaatttgaataggtcatcctctttctttacaatttgataatgtttgccgttgaaaaccaaattgactccggtcactacgttttccaattttctaaagaatcctttacacacaatgtcaggcggcaagtttagcgccatcacattctcgtacgtgtacgcccacaattcatcgtgatccaaaatttcgtttttagccgactgagtcaaatatatcatgtagtgtatgccaaaataatagcccaacgatacgcacaatttggtatcgtcaaagtcaaaccaatgattgcaggccctattaaacactattttctcttgttttttgtaaggctcacatcgcttcaaagcttcattcaaagcttctttgtcgcaggcaaataatgattcacacaaaagttccaaaaacagtttgatgtcggtttctctgtacgagaaattttcgttcttggtcaatatcttccacagtacatagattaaaaaatcaaaatttttaaatttgcttttttcaaagtattgttgtagaaggtttggatcgttggctcgttcgtgggtcgccaaaactttaaccatgttctcgtgaattgctataagccccaaattgatttgcgtttgaatgtagtctgcattttcgctgctcgccgatataatgggtacgatgcgcggttttctggaacgcgtgtcgctcaagtccacgtcgtttttgtcaaaattgttgttctcgaacactctgaggcttttgaggttgacgttgacgatatgcttgtacttgggcaccgtaatgcattcctccaaattaatgtcgtccctaatgtaattgaaaaaatttttatccgaattgaccagctcgccattaactttgcacgtggccacagtgccgtcggccattttgagtataaacaagtcttcgtgagaatcgtcaaacttggtttttccatttacaaacagcgtttgcggcggatcgtgattcgtgcgcaggctgagctcgacgttgagaaaacatttagggtcaaacacaaacaaatccacagggcctagttttttgttgtgtatgattggtatcgtgggttcgatgacaattccaaattttatatttaaaaacagctgccatccgttaaaagagaaagcttgctttttgggccagttgggccaataatagtaatcgcccgcttgcacgcatttgttaatgtatccagggtcggtgctcttgaaaaaatcttcaaaattaatatacttttgtatgatgtcatagtgcttcttcaaaatgaaaggttttacaaaaatgcaaaaatcgttactttccaacacccagtcgtggccgtctaatgtttgagctgcgtgtttctctgcaggttcttcggtgtcttcgcaagatgcgcccatgtcgtgtttcgcgcacggaccgttaaagttgtttctaattgtgtttaagaactgttgaaagttgttgacgtactcaaacaatctacgtgttcctgttcgcgtgtttctaatgattaaatgatttgcatcttgcaagttgttaatctcgtacgttttgtcttgaggcacgtttttcaaaaaaaattgtaaaatgttgtcaatcatgttggctatcgtgtttgtacttttcgtgttaatttatttaataatttcgatcaaaaatcaccatccattcttacatagaatagaaacgctaatacaagatttcaacaacacattgttgtttggcgcgtatgtacagatttacgatttaagcacgcccgcccgcaccgaacgattgtttattattgcgcccgaaaatgtggtgttgtataattttaacaaaacgctctattattacttggactcggcgaacgtgttttgtcccaacgagtttagcgtgaccacgttcacgcaatccactattaaaacgatcaacgagacgggaatatatgccaccgcatgcacgccggtcagcagcttgacgctaattgaacattttgcaacattaaaaaataacgtgcccgatcacacgctcgttctcgatgtggtcgaccaacagattcagttttcaatactcgacattatcaattatttgatttacaatggctacgtggatttgttggccgaataacgcgtatatagacgcttgtacgttcatcgtagtaatcattttaatacatttgattgaactaaacatacatctgcaatgggtgaaagagtcactaaattttgcaatggaaaacggcgataaagaagacagcgacaatgaatagagtttatatttttatttaataaaatattgttcgtaatccataatgttttgtattatttcattgtgataatgttcccaatcttgcacgggggtggggcatcgtttgactttgacgtagaaatcgtacgcgtagttattagttggcagatcgtcgacaagtgtgatcgacttgaaaaagtttacatttttatcgctcaaatatttaattacaatttttggcgatttgggtatattgttgtcggatcgatgattgtgaatgtcaaaaacaaatttattttcaatgaaacgcttttttaaattgtaatctacaatagcgttgtgtgaattttgaactaaatcagagcgttcttcttgaacggtggaaccttcgctgataatgatatcaaaatagccttccaaatcgacgtctcgcatcgagtgtgctacatgatctctactgccatacgaccacaagactaaaacgcaacccatctcgtgcaactcctgcaagctgtcatacacaaacggatctcgaatctcaacttgctcctcttcggttatgagagtgctgtccaaatcaaacacgaccacgtgcggaaatccccacgtcaaagattcgcttttgagagagaccactttgtagtgtggcaatagaaaccattctttaagaaacgaatacattggcggtttgttgctaagcacgcacatgtggcccaacactggcgttttgaatgcgcgtttaatattgtgcctgatgtcgcgcatgtcgtcggcgggcgctttgaatatttgcatacagtaattgtaattgttttctatgatcttgcacagctgcgggtcgttgcaaaattgaaatattacatattcaaaaaatttatacttttcaaagccaaggtatttgaggtcggcgtactcgcttaaaacgagaacatgtcgtttgatgatggcgtcgttaaggcgcaaacagatccatttgctttgaagcgaggaggccataatgtacaaaaatggaccagttacgccttatttaaactgtttaaagagtttcgtataaacaaaaactactctaaactaatagatttcttaacagaaaattttcccaacaacgtcaaaaacaaaacgttcaacttttcgtctaccggccatctgtttcactcgttgcacgcgtacgtgcccagcgtcagtgatttggtgaaagagcgcaaacaaattcgattgcagacagaatatttggcaaagctgttcaacaacacaataaacgatttcaaactgtacactgagctgtacgagtttatcgaacggaccgaaggcgtcgattgctgttgtccgtgccagctattgcacaagagtctactcaacaccaaaaattacgtggaaaacttaaattgcaaactgtttgacataaagccgcccaaatttaaaaaggaaccttttgacaacattctttacaagtattccctaaattacaaaagtttgttgttgaaaaaaaaggaaaaacataccagcactgggtgtacacgcaaaaagaaaatcaaacacaggcaaatattgaatgataaagttatttatttacaaaacagtaataaaaataaactatttgagcttagcgggcttagtttaaaatcttgcagacatgattttgtaacagtcgaaagccaaacgagggcaggcgacgaaatcgcttcgttcattcgctactgtcggctgtgtggaatgtctggttgttaatagtagcgtgttctgtaacttcggcgacctgtcgatgaacggctcctggatcttctgtatgtgcggggtctacccgggcggcgtctgtaacccgagcttctgcgcctgcgtgtcgaaccatatgtggtaccggttgaagaacggcgacggcgacgataaaccatgtttaaattgtgtaatttatgtagctgtaatttttaccttattaatattttttacgctttgcattcgacgactgaactcccaaatatatgtttaactcgtcttggtcgtttgaatttttgttgctgtgtttcctaatattttccatcaccttaaatatgttattgtaatcctcaatgttgaacttgcaattggacacggcatagttttccatagtcgtgtaaaacatggtattggctgcattgtaatacatccgactgagcgggtacggatctatgtgtttgagcagcctgttcaaaaactctgcatcgtcgcaaaacggaatttcggtaccgctgttgatgtattgttgcggctgcaacatttgtatcttttcgccgcgctcgatcaacaattcttcaagagtggtgcgtttgtcgcgctgtaaagccacgttttgtaacagcactattttcgcatatctcataatcggactgttgaaacagcgtgcaaacgacgaccgcataatatcgacggtcgtcaagtcgattgtggtcgaaggcatctccaacagagatcgcacggcgtccaacagcgtgtccgtttgaacctgcgtcatttgcggtctgcacgtgtagtcgtcaaacgtggtttcgagcagtttgaacaacgaatgatacttttccgatcgcagcaaaaatatcatggtcatgaccacgtcgctgattttgtattctgtagaactggtgctgttcaacgaatagtgatggattagtttgcgagcagcatttctgtatcggcgcatgttgatcaactcttcggaaggctgcgcgggcgcggcggcgttggctcgcgcaaacaaatttattacgggacgcggcgtaggctgcgcggacgctggcgcggcgacgacgtccgcgtttcccgccgcgtactgagacgctatggcagcgttgttatttaaaattgtgttttgcgatttgcgagccacgtgcatcataaaatttatcaacacgtcggtgttcaactgcacgctttgatgttcgtcgcagagcaaaggaaatagctggggccatatcgccaattgcataggctcgtctatttttaaccgcaatttgtttatttccaaatacaacgcgatagcgctcatcgtgaccgacgacgcacacttactctgtaactatcacttggatcgtgttgtcgtaaacgcttcccaaaaagtctaacacgttgaccgtttcgattctattcaacttaattgtggacgcgttggcttgcatcggttccaacagactgcgcgctccgacagattgagtagacaaaatttttaaactttccgtcttattgggcgtaatgtcgttgattaacaacgacgcagccgtttgagaggccgcagtgttgatggtttgcaacatgtcgacggccgccatttgcgtttgcgccgaaggtcttgctggcggcctgttgcggcggtttcttcgtgcttgcgacatgttgtcgtcagtgtccatatcggtatcatttattgaagcaatcatggttgagttcgataagcagagatatttcgttgtccaattggtacttggtaatgatgtgccttataaatgtttcgggcacaatcatttctgtcattagcacgttacaaatatctattttgatcaatttcaatttatgaattaacagattaatgttttcgtccgagtacttgctcatgatgaaacgacaaacgttgcggagttccaactccgctaccggatacgctttgttgggcaaactctctaaatagtgtctcaaataaaagccgatcaatacggtggacgctattttgttaacctttttcattttagtattgcggcccatttctatcatgaagtttttaaacggtagcaacagcctgtctccgttagcaacagtggagcagccgttgcattgcgcgctcaaaatactcaacacgcgctcgtgatcttcttggcgcaatccgacggttgcttttttgcattctttgacaaatggcacgcacatgtcgcgtttcgtgtacaaagaatacgctttgtcgcaaatcaagttatagaaaaattgcacaaatatctgcgtaatcaagttgttttcgttaataatgtcactttcgtttttgtaatcggttcgaagcaacacgtacaacatcagaggcatgccgaacatgggtcttaaaaaaatgtcccaaccattttgcaagcccgcgtcgagggtgctcagcgaggacgccaagtatttgcatttgcactcaaaacattgaattttgtttgcgggcttgcacgactgacacatgatcgcatccacgtcgggtgccggcgtcggattgtaatatttttgcaagtattgcataatggtcctaaaatggggtacctgtttgataaactcgtcgcgcaaaaatatcgaaaaaatgttttttacattgtgtatgttgtctgtgttgttggcttgattctcaaaactactctttatggaaacaatacatttgttaaattctgtgaaaaaagtaagacctttactgtccacgatcaagctttggttgaaatattttgaaaataaaaaacacaacgaatcgatttcatcttgtaacaattgcgcttcaaaacacacgttttcaaagcggtcgtaaatgttaaaccttaaactgtattgtaatctgtaagcgcacatggtgcattcgatataaccttataatatgaacgattccaattctctgttgattacgcgtttggcagcgcaaatactgtccagaaacatgcaaacggtggatgtgattgttgacgacaaaacgctcagtttggaagaaaaaatagacacgttgaccagcatggtgttggctgtaaatagcccgccgcaatcgccgccgcgggtaacatccagcgacctggccgcatcgatcattaaaaataacagcaaaatggtgggcaacgattttgaaatgcgatacaacgtgttgcgtatggccgtcgtttttgttaagcattatcccaagtattacaacgagacgaccgccggtttagttgccgaaatagaaagtaatctgttgcaatatcaaaattatgtaaaccaaggcaattatcagaacattgagggttacgatagtttattaaataaggcggaagagtgttatgttaaaattgatagactatttaaagagagcattaaaaaaatcatggacgacacggaagcgttcgaaagagaacaggaagcggagagattgagggccgaacaaactgccgcaaacgctcttctggagaggcgagcgcagacgtccgcagacgatgtcgttaatcgtgccgacgccaatattcccacggcatttagcgatccgcttccaggccccagcgcgccgcggtacatgtacgaaagttcagagtcggacacgtacatggaaaccgcccgacgtaccgccgaacattacaccgatcaggacaaagactacaacgcggcgtacactgccgacgagtacaattccctggtcaagacggttcttttgcgtttaatcgaaaaggcgctggccactctaaaaaatcggttgcacataacaactattgatcaattgaaaaagtttagagattatctgaatagcgatgctgatgctggagaatttcaaatatttttaaaccaggaagattgtgtgatactgaaaaatttgtcaaatttagcgtcaaagtttttcaacgttcgttgcgtggccgacacgttagaggtaatgttggaagcgcttcgcaataatattgagttggtgcagcctgaaagcgatgccgtacggcgaatagtcataaaaatgacgcaagaaattaaagattcgagcacgccgctgtacaacattgccatgtacaaaagcgattatgacgccataaaaaacaaaaacattaaaaccttgttcgacttgtacaacgacaggctgccaatcaatttcttggacacgtccgcaaccagtccagttcgcaaaacttccggcaagagatctgcggaagacgacttgttgccgactcgcagcagcaaacgtgccaatagacccgaaattaatgtaatatcgtcagaagacgagcaggaagatgatgacgttgaagatgtcgactacgaaaaagaaagtaaacgcagaaaattagaagacgaagattttctcaaattaaaagcattagaatttagcaaggacattgtcaacgaaaagcttcaaaaaattattgtggtcaccgacggtatgaaacggctgtacgaatactgcaactgcaaaaattctttagagactttaccgagcgccgctaactatggcagcttgctcaaaaggctaaacctgtacaatctcgatcatatcgaaatgaatgtaaatttttacgagttgctgtttccattgacactgtacaatgacaatgataacagtgacaaaacgctttctcatcaattggtaaattacatatttttggccagtaactattttcaaaactgcgctaaaaacttcaactatatgcgcgaaacttttaacgtgtttggcccgtttaaacaaatcgactttatggtcatgtttgttataaaatttaactttttatgcgacatgcgtaattttgccaaattaatcgacgagctggtgcccaacaaacagcccaacatgagaattcacagcgtgttggtcatgcgggataaaattgttaaactagcttttagtaatttacaatttcaaaccttttcaaagaaagacaagtcgcgcaacacaaaacatttgcaaagactaataatgttgatgaacgcaaactacaatgttatataataaaaaattataaaatatttttaatttttatttatattcagtacatttacacatattaacatattgtttatacaaattcttataatcattatgatttaaattgaattgttgtctaaacaaattaaacactttattaaacaataacttttcgttgtaattttttactttgcacatgttataacaaaaaattaaaattttcatcatgtctgatttgtctatggcgtcacagttgcttttaatgtaatcgcaagttaaccactcaaaaggacccttttctatttttaatttgtttaaatctttataatcagacttcagtttgtaaattagatttccacatcgaataataaatccttccagcgggctttggggaaacattaaagacttgaaatttaacctttctacaaaatcgttgtacaaatatttgtgacacggaatagtattaaaccccacgttagtcaacaactcttgcgcctccacaaagggcacaaactccccgccgtataattgaatttcgtaagcgtagtatttcaaactctctttctggtccacgtagttaattacgttaatgggtgtcgtttttgcgtcgtctttccaacccattaattcgccgtagacaataaaaccgtcattgaaccgcgcctgaagcgatcgcatgcacgtttctaaatcttttcgaatgcggtaataattcataaaattgccgtccggtctgtaagtgtttcttgacccgtacgtaattttattttggttgcaaatgattctgaaattacaaccgtccaacttttcttgaacaataatttctttgtcggccaacgtaccttttttaccttgatctagatgcgacacagatggataaatttgatacacaattttattctcatcttcgggcattacgggtccgcgttcatttaacgcgtacatgacaatgttgtggcgaatgtcggtgcgctccggcggttctggcacgtggtgcagtctgtcctgcaattgttgcttccattgttgaaaatattcggtccattcttgttgatactcgccgcgttgcatgagttttacgtacagttttaaaagtttgacattctttacaaataacgttagagtttcgtcgattttgtatcctccattatttttgtttaaatccaatacatttaaatcgttcactaccagttgattgtttttatccatcgtaatttttatctcatcgcccacgttgaacaacatgtttaaaattttggtggatttcggcgcacgtttataatctaaataatattcaacgtacacgtaattgaacatgagctgcaacaatcctttggcattgttcaaaattttgtatctcatcaaagtataaataattttcaccatcgacaccgtcatcaacttggttacaaactcgtacaattgcaagttttcaataccgtatttgtctttaaaatcttcacgtttactgaacatgcttaattcgggagattttccagtcaaaatgccaattaatcccgtgtacaagtcaacgtatttgacatcgttgcccgattcatcttttgcatgtcgatttttcaaaagctctttattgtcgataaatttttcaaaggtctctcgatcacatttagtgtaaatatggtagtcagtgtcgctgctttcgaccgcgtatcccttggcatggctgcccgtatcaatgcaaatgtacaccatgttagaatgtgctgcttactgtgcctgtatcaagccttatatacctcaaaatatttcacatttttgcatcatcgtaaaatatacatgcatataattgtgtacaaaatatgactcattaatcgatcgtgcgttacaagtagaattctactggtaaagcaagttcggttgtgagccgtgtgcaaaacatgacatcataactaatcatgtttataatcatgtgcaaaatatgacatcatccgacgattgtgttttacaagtagaattctactcgtaaagcgagtttaaaaattttgtgacgtcaatgaaacaacgtgtaatattttttacaatatttaagtgaaacattatgacttccaataattttgtggatgtggatacgtttgcaagacaattgattacagataaatgtagtgctctaatcaaagtgcggatctgttgccggcaaacattttagagattgtagagaaggccagagacaagtattttgagggccaactcaaaaaaactatgaatacattaaaaaattatttttacgaaaaaatatatggacgattcgatagattataaagattttaacagacgcatcctattgatagtttttaaattcgctttaaacaagagcacaatactttccatcgtacaaagagatcatcgagtggccattaaacgtttaaacaaaattaaccccgatttaaagagttctccgcgcaatgcttcagcattacaatgaatgtttggaaaatctagacaatccagtcacggacgaacatcatttgttaacaaaagagttgctacaaaaatatttatcgaagcgtttgaatacagttacaccaacactaatgccatcagcatggacaaaacagatgaatttgattttattaaaccggcattgaaacctttgccagatgcaagaccgccatcgcttttggccaacgtgatgaacgaacgtaaaagaaaattacaaaacaccaactcaacggcaaaatgtttgctaccagcaccaccgccacaattgcgtaaacttgaaaaaaagaatcatttattgcctttgttttctttgtaattatattgttgcatttctatttctaatatcatagttttctaataaagtagtttcatatttttgtttttgtacagtaattgtttcttggtttaacaagatcacaaccaataacataaagaataacacaatcataacaaaaattaaaaagccgcatactactagaacaaattctttaattagcgatcggtttctatttacaaattggccgagctgatcgccttcagtcggcgagttgtgggcttggatgatgtcgacgatattgttgccggcgcgaccgcccgtcgctctcgatataatgtcggccgccgtcggtttcatgatgtgcttaactacaaataatagttgtacttgacgggcgtcaccgtgatgccgctgctaaaacctccgtccgttaagacgcgttgcgttacaaaattaatgtttgtccgattagcgtagtcggaataatcaaacgtgttgggcggactaaaatcgggcatgttgatgggcacaatgccgctggagctgatagcaatgctgtcgttcttgcaaaacagccgaatttttttgtagggctctgctttattcggcgcagacgacaccatctggtcaaagttgttcaattttatgattacgttgggtaccaattgataggggaaaattattttctggaacattttgacaaagtccacaaccgtttggctatagtcgggaatgccgagcaaagactgcgcctgtttaatgtatttgagactggagcggtttactgtagcgcaattggatggcacgtcgcccttcataagccggcgcgttctctcccaattcaatttgttgtacaaattatcaatctcctcgtgcggcagattgattacatagcgcgcgggctgtttgcgatattgaaagatgcaaaaaatgcgtttcaacgacaatatcttcaccatggtggacgtttccagattgaaacataacaaaaagtcattgctttccaccaattctttaaaatgagacagcggaatttcacaagcgatcggtcgcaaattgctttttattggaggcggaacgctttgaccgttgcggttttttagtaacgcgctgcacgcagattgcatgtccgtttcgggatacgtaaactcgatgggacatttggggttttcatggtgaacgatcatagtgttgcaataaaacaagttgttggtcaggagcacgctaaaaacgcgcgtctcgcccgcaccgatttcggtgatgggcaccaacgggttccagtacactatggtggcggacgctgttttttttggcgatcgactgtctatgttaacatcatgctcgtgcctgtacactagcacagaattgaattttggaaattgttttttgtcaatgtacaaccggtcgtcgtctgtgggcacgtacacgatcaagttttcgattaatttgttgcctacgtcgctttgcggttccaccaaattgtgagggaacgcaaaaaagcgatcgctaatacaaacttgaatctgaaacgggcactccatcgcgatgtatatgtcttacttcattagactttagattattttaatttgtgaactcgtaccgtattcaatagggtgtcgggcacgtaattgtaatggtaaaacagatcctgttgaacatgtgcgttgttcactacgattgaaatgcaaaaatacatcaagtacataaacactatgattagaaaggtagcagatagaaaatatttcatctttaaatcttatgctagttgaataaaatacatagtacttttatacgtttatttatatttgttttctttgttataaccgtaattgtaaaacttgtgatcgtgctcgccaggcataatttctttgcacatcagcttgcgaatatatgtgacatcttcgtacaccgatttcttgatgttaccatcgtgaagcgttgtcggcttgagaggtttgcggtcgttgttgtaaaaattttgcaccgaataattatccatagtgcagcataggcaatgtcactgatgcatatgctttaattttttattgcattcagttattatatgatttaataaacgtacacaatagcacgtttatcggttaaagataactttcaatatataaaagcgtttgaattgcgagaccgtcaacataacgtttatcaacgcgatgactaaacgacaatttgctttgctgtttgtgtggcaccacgacaaccaatttgtttgcaacacggacgaatacccgttttggcacaacattgaataccatgcacggcgctataaatgcatcgttttgtactgtgtggaaaacgacggatcgctacaactgcccgtttgcaaaaacataaatctcataaattataaaaaagcgtatcctcattattatggaaactgtgttgacagtatagtgaaacgtgctggcaaaaattgattatatgaaagtaactgcaatgttaaacccccacctgttggacgtcgcgtacaattatttgctgttgatggacatggattgtgtggtgcaaagcgtgcaatggaaacaattgtcaaccgacacgtattgttttgagccgttttacgactctcaaattaaatggttgtacgcgcccaaaagcgggcaaagttttgatagttatcttgaaaactatgcaactctaattcgagtcaaacaagtgcagcaacatcgaaaagaattaatactgcattgtgtggattttcttacaatgaaagcaaatgacaattttatggtgttcaaaaattatattaacatgattataaaagtgtatttgcaattttacaattacagatttcctatcaattttgaggacaacacgatgaaaccttgtgtaaatttaacttttagacgtggcggcagttggaaaactcaactgcaacccgtatgcaattatgtttacaaaagtaaaaatatgccaaaatttattaaataaaacaaattaatttaaacaagcgtttttattgacaatactcacatttgatattatttataatcaagaaatgatgtcatttgttttcaaaattgaactggctttacgagtagaattctacttgtaaaacacaatcaagaaatgatgtcatttttgtacgtgattataaacatgtttaaacatggtacattgaacttaatttttgcaagttgataaacatgattaatgtatgactcatttgtttgtgcaagttgataaacgtgattaatatatgactcatatgtttgtgcaaaaatggtgtcatcgtacaaactcgctttacgagtagaattctacttgtaaaacacaatcgagggatgatgtcatttgtttttttaaaaccgaactcgctttacgagtagaattctacttgtaaaacacaatcgagggatgatgtcatttgtagaatgatgtcatttgtttttcaaaaccgaactcgctttacgagtagaattctacttgtaaannnnnatcgagggatgatgtcatttgtagaatgatgtcatcgtacaaactcgctttacgagtagaattctangtgtaaaacacgattacagcacttcgtagttgtatcgaaaattgttcaatggctctttgttaatgtcgtaattgattaatatgtcgtacaatttggcggcgttgtgtttgcacacgaccgtttttagttcttgaaacattttttcgtgtatgtttagcatgttgtatttcagagtgcgatgtgtaatgctggtgacgagcatcaaaatgataaaatctaaagcggctaatttgtaatcccgttcatacgctctgtaatcgccaacgactctgtggccagatctttttagattttgacaggcgttatggtacgaattgataatatttactatagtttctcttgttatcggtttgtcgattaaactgttaacaaacatcacgttgcccaagcgcgacggtttagacaccgacttgttttttgtctgttcaaatttgtacaaattaaaaacgctcatagactggtcgtcaggcagtgtgtcattatacaaacaaaatggtaaaacgtttaattcgacaaacgacgagcacattaaagtttgttggctgttaacgtcctggggatgtaaactgttattcataacgtaacacacttcaatgtcggaatgcttgttttcaaatttgtccttgtctacagtttcaatggtgattgagcgaggtttgagtttattttgtaaattcatttggatattttcaatatggtataccaccgacacgttgtgagccagcgatccttgattggttttaatcatattcaaaatattcatgatatggttgaaaaaagagtctgtcaaaacgtttgtgtcgttgttaaatatcgctttccagggtttactgttgcgtgactcaacgacggccgtgtaacataacaagcgcgccagttgcatgtgcgacaacttaatgttatcaatgtcggtgatgtttggcaccagattttcattgccgtcttccagtagcgtgctcagttcggtcgagtagttattcaacgatcgattgtgcgattcaaacaagtttactatcgcaggttgtacatagttttttatgtcgtcaaattgaattatatcgatcttgtccttgttctccagcataaacgacaaattttttaggtcgaatttaatatttggcgcgttttcgttggactttttgtaatttaacaacatcgccaacagtttgtgtaactcgccgttagcttgatctttgctaaacagtttattggtagcgtaattcacgttgtcgttcaaaaacagcaactcgttgatgatcattttttgtaaaagcgcgtacttgctcatgttgacagaatctcttacatttcagttgtaaacgcgtctgtacaaattggccatgcgattcggaatgcacacggggatcgtgcgagccagtgccgtttggcgaaatagcattttttcatagccgctcgaacaatcgcacgcgtccggcgaaaattgcaccgtgttcaaattcatattcaaccggccgtcgttgcatagataaggcctcggtgttcccgtatcgtccaccaagtctctgtacgtgctcacgcatgtttgagacacgacaaaatctccgccggcggagaaaacgtgaaccaaacccagtgcgggatcgcattctatcaagtccggagcctgcgcgtttaccaaagcgtcggaggcgttgcaaaagccatcctggcaggtcaactcgtttgcagcgctggagatcacgcagttgtctctacactgctgatccgtcacgcacggtaaccggttcaatgaacaatctacgcctcgattgcgctgaaacgtaaaatttaacggcggcgcttccaactcgttaatgtgcatgtatgcatcttgcaaaataaatttttgaacaaatttaaacgtgtacatgtacacgattagtataattaccagtagaataagtatttgccaaaagttcaacatgatcgtcttaactgagtgtgaaaagcgtggtgtgacgcacgaaatgactggttgcgcaaaaaataaaccggggtctatataactcggcgtcgaccgcgttcatttttaccgtcatgcatctgacggctaatgtattgctcgttcctaacgcgctcaaaaagcgggacgtgaaatacatttataatacctatttgaaaaattacagtgtaattgaaggtgtgatgtgttgcaatggcgattgtttggccgtggtggtgttggaccgaaatcagctgcaaaacacggacatggaagtgttggagagtttagaatacactagtgacaacattgaactgttatgcgaaaaaatatgtgtgatagttgataattacgacaagtattaccaaaaaaattgtgtataaataaaataccaaaatttattatatcattttgttttatttaataattaaagaatacaacgccacatctattcctagtacaacaaataatttgattattatttttgagtgcacattaaaaaataacaaacagtgtaaaaatactacagaataatacaatacataaatattatagtaaatagctgcaattttgatagcgtaatttatactttgatatttttcaacgtacaacgttaaatgttgatacgcattattcacaaataacaaaatttttctaatatgccatttgtccgcaattgtttttgcgatatcaaagcctttttcaaacaattgaaaaattgcaaacaaaaccacgtacatgacgttatacatagtgttaaagtttttacataacaattctataatgaagaaaattgctaaacacggcatgagcgcgcacataatcgcgttggccgcaaatatctcgtacgtacaaaaatactcggacattctccaataagtaaaatgcattttgctattatactgttgtttcttctagtgattattgcaatagtgtacacgtatgtagacttgatagatgtgcaccatgaagaggtgcgttatcctattacggtttttgacaacacacgcgcgccgctcattgaaccgccgtccgaaatagtaatcgaaggcaatgcacacgaatgtcacaaaactttgacgccgtgcttcacacacggcgattgcgatctgtgccgcgaaggattagccaactgccagttgtttgacgaagatacaatagtcaagatgcgtggagatgacggccaagaacacgagacgcttattcgagcgggagaagcgtactgcttggctttggatcgagaacgcgcccgatcgtgtaaccccaacacgggtgtgtggttgttggccgaaactgaaactggtttcgctcttttgtgcaactgcttacggcccggacttgttacgcagctcaacatgtacgaagactgcaacgtgcccgtgggctgcgcgcctcacggccgtatcgacaatatcaacagcgcttcgatccggtgcgtgtgcgacgacgggtacgtgagcgactataacgccgacaccgaaactccgtattgccgtccgcgcaccgtgcgcgacgtaatgtacgacgagagtttttttccgcgggcgccatgcgcagacggccaagttcgtctggatcatccggcgctcaatgatttttaccgcagacactttagactcgaagacatttgcgtgatcgacccttgctcggtggacccgattagcgggcaacgcacatcgggacgcttatttcaccaaccaaccgtaaatggtgtgggaatcaacggatgcaattgtccggccgatgacgggttactgcccgtgtttaatcgacacaccgccgacacgggcatggttagacaaagcgaccgcaccgtcgcgaacgcttgcttgcagccgtttaacgtgcacatgttatcgttgcgtcatgtggattacaaatttttctggggccgcagcgaccacaccgagtttgccgacgcggacatggtgtttcaagcgaatgtcaaccaactcagtcacgaacggtatcgagcgattttgtacccgttgctcgagtcgcacccggacgtaacagaaatcgtaacagtcaacatgggtgtcatgaaaatttccgtgtcatacgataccacattgaaaaatatactattaccatcttctgtttttaggctatttagatttaaagaaagtggcactgctcagccggtatgcttctttccaggcgtaggacggtgcataaccgtcaattccgattcgtgcatcaggcgacacgctggtggtcaagtgtggaccgcagaaacgttcaccaactcgtggtgtgtactgagtcgtgaaggtacgcatataaaagtttggagtcgcgcgtcacgatatccacgcggagacgcgcctgcagcgttaagattgcgcggcttctttctgaacaacgatcgcgaacgaaacacaataagagcggtcactacaggcgacatgacccaagggcaacaaatagacgcattaacccaaatacttgaaacttaccccaactactctgtataacaacatgagcattttaaaagttgtagaagcgtgcgatttggcacacacttttttgaaattgggttatttatttagggccaagacttgtttggatatcgctttagataatttggaactattgcgtcgaaagactaacataaaagaagtggcagtcatgttaaacaagaaaactacagagtgtttgcaattgaaacgaaaaatagataaaaaaattgcacaacgtgttttaataaaaatttacactatcaaatgatgacatcataacgggttcaatattctgtgtgcaaaaataaatgacatcatatttcaaacttgttttacgcgtaaaattctactggtaaaacaagtttgagatatgatgtcatcatcacaaataatagtatgtaataaaataaacatatttgtgtgtaaatataatttattacaaataaattttacattgaatcaatctgtcttcgtgtttgttgtaaggtcttcgaatcttgtgtttcagcccctcgggatggtcaaaatgcgccgtagtaattgttaatggatctttcaacgattttttgcccatggcgagtgtgacaaacgcggccacgacaaacagcaggataatcagtttcatggtgttctatattcgacaatatatgggtcgcttctaaatcaccttgtccccaaaagcctcttttatagttttttagaacacgttgtgtattccaacagtaattgttccatctctttcaacagccattcagcatccggtcgttgactgtaatcatgctgaattaatttacaaacaatttcggtcaatttaggatggccttgggataaacttgccggcatttgctgtacattgtttctaaagttagttagcgtagtttcgcgttccaaagcagtcttgaagggcattatcaattcgaataaaacaatgcccaaactatacatgtcatttttgggggtgtacacttttttgatttgttctggtgcagcgtacaaagttatattttgagggttgtttttgataaacgttttgtatagactgccaaacatgccgcccacatacaaatcaaagtcgggcccagtcatgaaaatatcttcgggattaatattgtggtgcacgatatttacggaatgaatcgctttcacggcgctcaccaaatcaacaaacttgctaatataaaagccaaaatccgccggaactttaatgttggtctttgcaaaagtttgcaaattgcgttgtttcaaatagtcgctcaacatgtactcgtttagaggcgacgcaaaatatatgcggtgctgccgcggattcaaataaaccaattgttcgggtttcatggtatacagttaagtgttaacgcgtcactaaattcagacacgagcgcacgccctatatacatacaatttatcgcacaagatgcttaacgcgatctgtttataaactaaaacgcactgcaataaattttagcaagcatttgtatttaatcaatcgaaccgtgcactgatataagaattaaaaatgggtttgtttgcgtgttgcacaaaatacacaaggctgtcgaccgacacaaaaatgaagtttccctatgttgcgttgtcgtacatcaacgtgacgctgtgcacctacaccgccatgttggtgggatacatggtaacattcaatgactccagcgaattgaaatatttacaatactggttgctgttgtcgtttttgatgtccgtggtgctaaacgctccgactctgtggacgatgctcaaaaccacagaagcccatgaagtaatttacgaaatgaagctgttccacgccatgtactttagtaacgtgctgttgaattatgtggtgtttttggacaatcaaatgggtacaaattttgtttttgttaacaatttaattcactgttgtgtactttttatgatatttgttgaattgcttatcctgttgggccacacaatgggcacgtacacggattatcaatatgtcaaatcgtgttatatggttatattgtttgtttcagttatgagtgttactattgttatgggtttagagtgtttgaaaacgaaactaattgataacagtttgatgtttaacgcgtttgtgtgcgctttgtacattgtgattgcaataatgtggtctttaaaaaataatttgactagttattacgtttcaaatttacaaagtattcaagttgttccgttttcatacaacgatccgccgccaccgttctctaacattgtaatggatgacataaaaaataaaaaataatttataaaaatgttttttattctttcacaattctgtaaattctaaacaaaaaatataaatacaaacttattatgttgtcgtctaaataaacatcaatttgtaaatctggacacctattcatatcattgatattacagtctactatacaacaattaaaactaaccaaattatctttacaacaattaaagcaattaaaacaatttaaataatcttcattgtcgtcgtataagtttatttgcactgtagacggtgttacacagcgatccattcgacgttcgtgttcgatcaactttctcgccaacttgtaccataaaaattgtttggacaaaaagttttccaacaatggtaacggccaattcaacgtgacgatgcgcacgtcctcgggtatgcatttgttaaaaaacacacagctcgctttaccaaacgaaagcaaaggtactaaatatggcgccattggctgatttgttattccaagataattacaaataaactgatccgtcgtggggtgataactggcaggtgtcagctttaaataatcttcaacgttgttgtcgcgcaaaagtctgcattttacacgcgttgttaatcccacgacttttgcatgtaaaatcggatccaaatactgcagaatcgtgtctataatttctaatggtaaacgtatgcgttttgctcgtgggcgctttgtaacgctcgacatcctaataacaactaacacaaaactaaaatgatactcaatatattgcttttacagttcatctttaggtttaaactgtgcgtttatcgcgttgagcaagtcgccgttatcggcatcaatctcccaagcaaacaggccgcccaatttatttcggtcgacatatttaacttttcctaacacagagtcgacgctgtcaaacgaaatcaaatcacctttacttttatcgaaaacgtacgacgcttgagcggcgctgtcaaacgtgtacacataattgttgagatctttttgaatttgacgataatctacaacaccgtcctcccacgtgcccgacaccggcccgttgccagtgccggaaaaatagttgtcattcgtataatttgttacgccggtccagccgcggccgtacatggcgacgcccacaattattttgttgggatcgacgccttgtttcagtaacgcatcgacagcgtagtgtgtagtgtatagctcttccgagttccaacttggcgcgtagactgttgtttggtagcccaaatccgttaccgttcgtatagcatacattatacgaacggtaggccctattcctatggccatagacgctgccgacattgttaactataaacagggtattataaaatattgtttcaacagcggtctaaaccatgcggttcttttagtgggttatggtgttgaaaacaacattccatattggacctttaaaaacacttggggcacggattggggagaggacggatttttcagggtacaacaaaacataaacgcctgtggtatgagaaacgaacttgcgtctactgcagtcatttattaatctcaacacactcgctatttggaacataatcatatcgtctcagtagctcaaggtagagcgtagcgctctggatcgtatagatcttgctagaattgtgagttcaagtctcgcctgagatattaaaaaactttgtaattttaaaaattttattttataatatacaattaaaaactatacaattttttattattacattaataatgatacaatttttattattacatttaatattgtctattacggtttctaatcatacagtacaaaaataaaatcacaattaatataattacaaagttaactacatgaccaaacatgaacgaagtcaatttagcggccaattcgccttcagccatggaagtgatgtcgctcagactggtgccgacgccgccaaacttggtgttctccatggtggttatgaggttgcttttttgttgggcaataaacgaccagccgctggcatctttccaactgtcgtgataggtcgtgttgccgatggtcgggatccaaaactcgacgtcgtcgtcaattgctagttccttgtagttgctaaaatctatgcattgcgacgagtccgtgttggccacccaacgcccttctttgtagatgctgttgttgtagcaattactggtgtgtgccggcggattggtgcacggcatcagcaaaaacgtgtcgtccgacaaaaatgttgaagaaacagagttgttcatgagattgccaatcaaacgctcgtccaccttggccacggagactatcaggtcgtgcagcatattgtttagcttgttgatgtgcgcatgcatcagctcaatgttcattttcagcaaatcgttttcgtacatcagctcctcttgaatatgcatcaggtcgcctttggtggcagtgtctccctctgtgtacttggctctaacgttgtggcgccaagtgggcggccgcttcttgactcggtgctcgactttgcgtttaatgcatctgttaaacttgcagttccacgtgtttttagaaagatcatatatatcattgtcaatcaaacagtgttcgcgtgtcaccgactcggggttatttttgtcatctttaatgagcagacacgcagcttttatttggcgcgtggtgaacgtagacttttgtttgagaatcatactcacgccgtctcgatgaagcacagtgtccacggtcacgttgatggggttgccctcagcgtccaaaatgtatacctggcactcgtccgtgtcgtcctggcactcgagcctgctgtacattttcgaagtggaaatgccgcatcgccacgatttgttgcacgtgtggtgcgcaaagtgattgttattctgccgcttcaccaactctttgcctttgacccactggccgcggccctcgttgtcgcgaaaacagtcgtcgctgtcactgccccaacggtcgatcagctcttcgcccacctcgcactgctgcctgatgctccacatgagcaaatcctctttgcccacattcagcgttttcatggtttcttcgacgcgtgtgttgggatccagcgagccgccgttgtacgcatacgcctggtagtaccccttgtagccgataatcacgttttcgttgtagtccgtctccacgatggtgatttccacgtccttttgcagcgtttccttgggcggggtaatgtccaagtttttaatcttgtacggacccgtcttcatttgcgcgttgcagtgctccgccgcaaaggcagaatgcgccgccgccgccaaaagcacatataaaacaatagcgcttaccatcttgcttgtgtgttccttattgaagccttggtgtgactgatttactagtagcattgaggcatcttatatacccgaccgttatctggcctacgtgacacaaggcacgttgttagattaataatcttatctttttatcttaattgataagattatttttatctggctgttataaaaacgggatcatgaacacggacgctcagtcgacatcgaacacgcgcaacttcatgtactctcccgacagcagtctggaggtggtcatcattaccaattcggacggcgatcacgatggctatctggaactaaccgccgccgccaaagtcatgtcaccttttcttagcaacggcagttcggccgtgtggaccaacgcggcgccctcgcacaaattgattaaaaacaataaaaattatattcatgtgtttggtttatttaaatatctgtcaaattacaatttaaataataaaaagcgtcctaaagagtattacacccttaaatcgattattagcgacttgcttatgggcgctcaaggcaaagtatttgatccgctttgcgaagtaaaaacgcaactgtgtgcgattcaggagagtctcaacgaggctatttcgattttgaacgttcatagcaacgatgcggccgccaacccgcctgcgccagacattaacaagttgcaagaactgatacaagatttgcagtctgaatacaataaaaaaattacctttaccactgatacaattttggagaatttaaaaaatataaaggatttaatgtgcctgaataaataataataagggttttgtacgatttcaacaatgaacttttgggccacgtttagcatttgtctggtgggttatttggtgtacgcgggacacttgaataacgagctacaagaaataaaatcaatattagtggtcatgtacgaatctatggaaaagcatttttccaatgtggtagacgaaattgattctcttaaaacggacacgtttatgatgttgagcaacttgcaaaataacacgattcgaacgtgggacgcagttgtaaaaaatggcaaaaaaatatccaatctcgacgaaaaaattaacgtgttattaacaaaaaacggggtagttaacaacgtgctaaacgttcaataaacgcttatcactaagttaatatactaaaaatcacatagtcactacaatatttcaaaatatgaagccgacgaataacgttatgttcgacgacgcgtcggtcctttggatcgacacggactacatttatcaaaatttaaaaatgcctttgcaggcgtttcaacaacttttgttcaccattccatctaaacatagaaaaatgatcaacgatgcgggcggatcgtgtcataacacggtcaaatacatggtggacatttacggagcggccgttctggttttgcgaacgccttgctcgttcgccgaccagttgttgagcacatttattgcaaacaattatttgtgctacttttaccgtcgtcgccgatcacgatcacgctcacgatcacgctcgcgatcacgttctcctcattgcagacctcgttcgcgctctcctcattgcagacctcgttcgcgatctcggtcccggtctagatcgcggtcacgttcatcgtctcccaggcgagggcgtcgacaaatattcgacgcgctggaaaagattcgtcatcaaaacgacatgttgatgagcaacgtcaaccaaataaatctcaaccaaactaatcaatttttagaattgtccaacatgatgacgggcgtgcgcaatcaaaacgtgcagctcctcgcggcgttggaaaccgctaaagatgttattttgaccagattaaacacattgcttgccgagattacagactcgttacccgacttgacgtccatgttagataaattagctgaacaattgttggacgccatcaacacggtgcagcaaacctgcgcaacgagttgaacaacaccaactctattttgaccaatttagcgtcaagcgtcacaaacatcaacggtacgctcaacaatttgctagccgctatcgaaaacttagtaggcggcggcggcggtggcaattttaacgaagccgacagacaaaaactggacctcgtgtacactttggttaacgaaatcaaaaatatactcacgggaacgctgacaaaaaaataagcatgtccgacaaaacaccaacaaaaaagggtggcagccatgccatgacgttgcgagagcgcggcgtaacaaaacccccaaaaaagtctgaaaagttgcagcaatacaagaaagccatcgctgccgagcaaacgctgcgcaccacagcagatgtttcttctttgcagaaccccggggagagtgccgtttttcaagagttggaaagattagagaatgcagttgtagtattagaaaatgaacaaaaacgattgtatcccatattagatacgcctcttgataattttattgtcgcattcgtgaatccgacgtatcccatggcctattttgtcaataccgattacaaattaaaactagaatgtgccagaatcagaagcgatttactttacaaaaacaaaaacgaagtcgctatcaacaggcctaagatatcgtcttttaaattgcaattgaacaacgtaattttagacactatagaaactattgaatacgatttacaaaataaagttctcacaattactgcacctgttcaagatcaagaactaagaaaatccattatttattttaatattttaaatagtgacagttgggaagtaccaaagtatatgaaaaaattgtttgatgaaatgcaattggaacctcccgtcattttaccattaggtctttagatttggtaaggctagcacgtcgacatcatgtttgcgtcgttgacctcagagcaaaagctgttattaaaaaaatataaatttaacaattatgtgaaaacgatcgagttgagtcaagcgcagttggctcattggcgttcaaacaaagatattcagccaaaacctttggatcgtgcagaaattttacgtgtcgaaaaggccaccaggggacaaagcaaaaatgagctgtggacgctattgcgtttggatcgcaacacagcgtctgcatcgtccaactcgtccggcaacatgttacaacgaccagcgcttttgtttggaaacgcgcaagaaagtcacgtcaaagaaaccaacggcatcatgttagaccacatgcgcgaaatcatagaaagtaaaattatgagcgcggtcgttgaaacggttttggattgcggcatgttctttagccccttgggtttgcacgccgcttcgcccgatgcgtatttttctctcgccgacggaacgtggatcccagtggaaataaaatgtccgtacaattaccgagacacgaccgtggagcagatgcgtgtcgagttggggaacggcaatcgcaagtatcgcgtgaaacacaccgcgctgttggttaacaagaaaggcacgccccagttcgaaatggtcaaaacggatgcgcattacaagcaaatgcaacggcagatgtatgtgatgaacgcgcctatgggcttttacgtggtcaaattcaaacaaaatttggtggtggtttctgtgccgcgcgacgaaacgttctgcaacaaagaactgtctacggaaaacaacgcgtacgtggcgtttgccgtggaaaactccaactgcgcgcgctaccaatgcgccgacaagcgacggctttcattcaaaacgcacagctgcaatcacaactatagtggtcaagaaatcgatgctatggtcgatcgcggaatatatttagattatggacatttaaaatgtgcgtactgtgattttagctcagacagtcgggaaacgtgcgattctgttttaaaacgcgagcacaccaactgcaaaagttttaacttgaaacataaaaactttgacaatcctacatactttgattatgttaaaagattgcaaagtttgctaaagagtcaccactttagaaacgacgctaaaacacttgcctattttggttactatttaactcatacaggaaccctgaagaccttttgctgcggatcgcaaaactcgtcgcccaccaaacacgatcatttaaacgactgtgtatattatttggaaataaaataaacctttatattatatataattcttttatttatacatttgtttatacaattttatttacgacaaatattgactcgttgttcagaaagtttaataagcttgtcaatttcttcggcttgcaaagggctgccaacgcgttcgttttgaatgcgcgtaatccggtttacggtattgttggcgcgaacaataaactcctcaactggcaaattaacaattttgtttgcgtactcattgtgcactgcggccaggttttgtagaatgttttcgggaaaaatggcaattctattaaatttgacatgtttttgattgtatacatagttttgatattcttccagcgtaggatatttgtttaaactcttgacgcattcaatgtacaatttgtgcagtgacaaaattctgttaaaatccaaacgagaacatttctcaaaagttatttcttgaccgttgaaatgtacactttgcaattgtttcaataaactgtcgtaaaaagtttttccttcttcaagcacaaacgcggggcgcatcgtgttatctacaacgcttatgtacttgtcaaaatcttcaattatatgatagaaatacaaatatctctccgcgtttatggacgtgtcgtttaaaacatgttcgtcaacaactccgttatgatttactttcaaaaatttcaaatcttgcaaagcgtccgcgttggtcaacttgttgataataaatttgtctttgcattcaaacgctctgtttgcaatccactccacagcgtccaaaacggacatgcgtttaaacatgttgatacgttttagacaatacgctcgtttttttaccgcctcaacgttcacgtccgtgtagtcgcaccattgcaggatttgcaacatgtcctcggcaaaatgcgcgaactgccgcagcttttcctttccaaaatgttgattgtcgtgtttaaaaagcaacgttgaaatttccgagacataccacaaagccgtgggcaattttactttgatcagcggctccatagccaggttgctgaacccgatcatgcattccgtgttgttaatgcggtaaatgacatagcgtttaaagtagtcctttacattatcgtcaatgtattctgcgtcgtttatgtgcttgtacagcaaatagtacataaggcccgcgttaaacgcgacctttttagcgtcaaaatacgtgcacgccaacacgtaatcgttgtattcgtcgaattgctcgttgggcactatggcgcccgtaaaagggcgtctgctgcgcggtgacaaacgcgttccatgctgaatcaactgcttcaaactttccaaattataacaatattcaattgaatttttaatctctttattttggctccataaaagaggaaactcgagtcggcttttaaacttggtcaaactgccctgaattgtttcaaacaagttgtaatgtgttaacaatatggccggcacaccgctatcgttggctaaaatacaatcggggaatcgaatattttctacgttgctgtaatcgtacgcttcgtcgtcgtcgttggcaacaacatcgtcggtttcggcgtccacgctcgctaacttgttctgatagtgtaaatttttcattacatcaaaagcgtatgacttgttgcgattgtgcaaataatttatggccgtgctaatggtgctgtcgataattttatcaaaattgagaacatcggcgttatacaacgttttataaaattctgttgacttgaacgtgtttacaaactcatttttatttttaatctggtcaaaattcatactagaattgttagtttgtttgatttcgctgaatagccgctggcggagacgcttcagcttgtccacctcgtttaacacgttggcgtccgtcggcatggaattgataaatttgaaccgaacaaaagacagcagttcatcttttttcgatataaaattttcggttgtaatgatatcgtagttaaattctttggttaaattgacccattcgaccatttcatcgttgcgataaatcttgcagtccgagttgttgacaaacgccgaggcaacggacaaatcaatctgttccgtgttattattgatggcataaaacacaatgcgttcgaaactaaacggtttttcgtttagcaaatttttgcaaacgtttgcctcatttttggaaatttggccgtcggtcaccatgtacaaaagtttcaacttgccgtcgagcaagtttatattcttgtgaatccactttatgaattcgctgggcctggtgtcagtaccctcgccattgcggcgcaaataacgactcttgacgtctccgatttctttttggcggcaataagcactccaatgcaaatacaaaactttgtcgcaactactgatgttttcgatttcattctgaaattgttctaaagtttgtaacgcgttcttgttaaagtaatagtccgagtttgtcgacaaggaatcgtcggtggcgtacacgtagtagttaatcatcttgttgattgatatttaattttggcgacggatttttatatacacgagcggagcggtcacgttctgtaacatgagtgatcgtgtgtgtgttatctctggcagcgcgatagtggtcgcgaaaattacacgcgcgtcgtaacgtgaacgtttatattataaatattcaacgttgcttgtattaagtgagcatttgagctttaccattgcaaaatgtgtgtaatttttccggtagaaatcgacgtgtcccagacgattattcgagattgtcaggtggacaaacaaaccagagagttggtgtacattaacaagattatgaacacgcaattgacaaaacccgttctcatgatgtttaacatttcgggtcctatacgaagcgttacgcgcaagaacaacaatttgcgcgacagaataaaatcaaaagtcgatgaacaatttgatcaactagaacgcgattacagcgatcaaatggatggattccacgatagcatcaagtattttaaagatgaacactattcggtaagttgccaaaatggcagcgtgttgaaaagcaagtttgctaaaattttaaagagtcatgattataccgataaaaagtctattgaagcttacgagaaatactgtttgcccaaattggtcgacgaacgcaacgactactacgtggcggtatgcgtgttgaagccgggatttgagaacggcagcaaccaagtgctatctttcgagtacaacccgattggtaacaaagttattgtgccgtttgctcacgaaattaacgacacgggactttacgagtacgacgtcgtagcttacgtggacagtgtgcagtttgatggcgaacaatttgaagagtttgtgcagagtttaatattgccgtcgtcgttcaaaaattcggaaaaggttttatattacaacgaagcgtcgaaaaacaaaagcatgatctacaaggctttagagtttactacagaatcgagctggggcaaatccgaaaagtataattggaaaattttttgtaacggttttatttatgataaaaaatcaaaagtgttgtatgttaaattgcacaatgtaactagtgcactcaacaaaaatgtaatattaaacacaattaaataaatgttaaaatttattgcctaatattattttgtcattgcttgtcatttattaatttggatgatgtcatttgtttttaaaattgaactggctttacgagtagaattctacgcgtaaaacacaatcaagtatgagtcataagctgatgtcatgttttgcacacggctcataaccgaactggctttacgagtagaattctacttgtaacgcacgatcagtggatgatgtcatttgtttttcaaatcgagatgatgtcatgttttgcacacggctcataaactcgctttacgagtagaattctacgtgtaacgcacgatcgattgatgagtcatttgttttgcaatatgatatcatacaatatgactcatttgtttttcaaaaccgaacttgatttacgggtagaattctacttgtaaagcacaatcaaaaagatgatgtcatttgtttttcaaaactgaactcgctttacgagtagaattctacgtgtaaaacacaatcaagaaatgatgtcatttgttataaaaataaaagctgatgtcatgttttgcacatggctcataactaaactcgctttacgggtagaattctacgcgtaaaacatgattgataattaaataattcatttgcaagctatacgttaaatcaaacggacgttatggaattgtataatattaaatatgcaattgatccaacaaataaaattgtaatagagcaagtcgacaatgtggacgcgtttgtgcatattttagaaccgggtcaagaagtgttcgacgaaacgctaagccagtaccaccaatttcctggcgtcgttagttcgattattttcccgcaactcgtgttaaacacaataattagcgttttgagcgaagacggcagtttgctcacgttgaaactcgaaaacacttgttttaattttcacgtgtgcaataaacgctttgtgtttggcaatttgccagcggcggtcgtgaataatgaaacgaagcaaaaactgcgcattggagctccaatttttgccggcaaaaagctggtttcggtcgtgacggcgtttcatcgtgttggcgaaaacgaatggctgttaccggtgacgggaattcgagaggcgtcccagctgtcgggacatatgaaggtgctgaacggcgtccgtgttgaaaaatggcgacccaacatgtccgtctacgggactgtgcaattgccgtacgataaaattaaacagcatgcgctcgagcaagaaaataaaacgccaaacgcgttggagtcttgtgtgctattttacaaagattcagaaatacgcatcacttacaacaagggggactatgaaattatgcatttgaggatgccgggacctttaattcaacccaacacaatatattatagttaaataagaattattatcaaatcatttgtatattaattaaaatactatactgtaaattacattttatttacaatcatgtcaaagcctaacgttttgacgcaaattttagacgccgttacggaaactaacacaaaggttgacagtgttcaaactcagttaaacgggctggaagaatcattccagcttttggacggtttgcccgctcaattgaccgatcttaacactaagatctcagaaattcaatccatattgaccggcgacattgttccggatcttccagactcactaaagcctaagctgaaaagccaagcttttgaactcgattcagacgctcgtcgtggtaaacgcagttccaagtaaatgaatcgtttttaaaataacaaatcaattgttttataatattcgtacgattctttgattatgtaataaaatgtgatcattaggaagattacgaaaaatataaaaaatatgagttctgtgtgtataacaaatgctgtaaacgccacaattgtgtttgttgcaaataaacccatgattatttgattaaaattgttgttttctttgttcatagacaatagtgtgttttgcctaaacgtgtactgcataaactccatgcgagtgtatagcgagctagtggctaacgcttgccccaccaaagtagattcgtcaaaatcctcaatttcatcaccctcctccaagtttaacatttggccgtcggaattaacttctaaagatgccacataatctaataaatgaaatagagattcaaacgtggcgtcatcgtccgtttcgaccatttccgaaaagaactcgggcataaactctatgatttctctggacgtggtgttgtcgaaactctcaaagtacgcagtcaggaacgtgcgcgacatgtcgtcgggaaactcgcgcggaaacatgttgttgtaaccgaacgggtcccatagcgccaaaaccaaatctgccagcgtcaatagaatgagcacgatgccgacaatggagctggcttggatagcgattcgagttaacgctttggcagtcacggtcagcgttttgatggcgatcacgttgagcgagtgcactaacgcggctttgtaagtctctcccaacatgcgcacggtcacgcgccgagtcgtgctaagcaacatgtgtttcatggccggaatgagagaagtgttaatttttttcaacatgcttttaaacccggacattagcatatcaaagccaatgtccgtagcaataccgaaaacgagcgcgtaatcttccaaaaacgatgttataattgactccaagtcttggtcgctgattgaacggtcgagcgcctcgaaatgttcgacacgtgcacgttcgttaccgcggtaattgtatgcgatcggagttttagtaaagccggtttcggccgtgtacgtgatctggacgggcgacccgttgacgatcatgcccaaatcgtttagtgttggatttttgttaaaaagtttttcaaattccaagtctgtggcgttatcgcgcacgctgcgccattgcgctagtattgcgttggagtccacgttgggtcgtggcggtagtatgctggaaggcgctttgtaatcaaaatcgcgcagttcgctaaaaatgttgttggccagcattttgaaagtgacaaagatcgtgtcgcccagcacgaatccgatgagcgattcccaccatctaaacgaacaaccgccgttgaatagctctctgccgaaacgtcgacagtaggcttcgttgaattcgcctttaaagcgttcgggaaacaaggggtcgggatcgggccgaacgttaaaagccggcacatcgtccacgcccatgatcgtgtgttcttcggtgcgcaagtatgggctgttaaagtacattttggacagcgagtccactaagatgcatttgttgtcgagcgtgtatctaaactcggcagactgaacttgggtttcggcgccttcacgcatggccgccgccctgtccaggtggtagcacgcgggctgcgcgtaacccacgctagtctcggaggtctgcgtgtacatgaacggcgtcgtgttggacacgacgccggtttcgtgaaacggatagcagctcatgctttcacacccgcgcttgctgaaagccagtttgacggccagcgctttgtcggccaatttcggcggcacataataatcgtcgtcacttgacgcgggacgcagcgtgtagtcgattagtatatgcggaaacctggtgcgccatctcgaaataaactcgagacgatgcatatgtatggcatacctactggcattagttaaatcgacggctgttaaaaccgccatgttatataggacttaaaataaacaacaatatataatgaaatatttattagattatattatagcaatacatttacatttattataacaatactttttatttaatctgattatattataacgatacatttttatttagacattgttatttacaatattaattaactttttatacatttttaaatcataatatataatcatttcgttgtgcatttcaaagcttttgatagcttcaaagtaatacatgaatttagagtattcaggaaaatgataaacgttggtaaacccgcatttggtacaatataacacgggatttttataatacagtttagtttttttacacaatttgcaatagttgttagttgtaggtttcaaaggaaacgtgattgcgccgtccaatacctgggtaaactttttgactttaacagtggcaaacacggttcctttgatacccgaaaatcggttgtcttgcagagcggccatcatttcgcttggctcttgaagtataaaacagttgacgtcatccaccacgtcgggtctggtgcacatgcttcggtagcgctgcaacactatattggtgtatgtttccctgagaacgagaccgccggtggtgctaagatcgattgtttgaatacgctcgttgggctctttgtgatttcgaattatgcgccgaattatttcaaacactttgcagttgtgatcgtcaattctcaattctttaacttccgtcgtgtgctctaaacttacagggaaaatgtattggtaaaaaaacctctctctggctaaatagctgaggtcgaccaaattgatagaaggatatatttcgtacgaggtttttggaacgttgtgatatagatagcatttttgacagcagatgtctatgcggtcaggatcgtccaacggcttttcgatgtgaaccacaacatacaaaaaccattcgcgcgtgttgtctttgaatctataattgcaagtggtgcatcgcgaatcgctcatgtgctccatagtcttcttgtatttcacaggcctgcttgcaaatttgcccgtcatgcgcatatctttgctgtttatgtagcccataatgtaattggtggaaaattttagcgtggctttcatgatgtcgcgttctaaatcgctcatgaaatgcatacgtagatcgcgctcttgtttgaaatccagtttgtcgctgtacgcgggcaaaccttcaaacttgttcccaaactcgggcggcacaaaatatccatcttttctgttgacgactggttttttacttacaatgctgctgtgctccaacggcttggccggagaggtgcgcgtaggctgtttaggcggagagatgcgcgtaggtggtttgatgttagattttggcggcggacgaacaggcgacggcggcgagttggcggcaggcgctggcaaagatttggcacgacccttgcccccggtccttggcgcgtcaaaaatgttattctctcgaaaaaaacggttcattgtaactgttagttagcactcagaaatcaacacgatactgtgcacgttcagccatcgagaggctttatatatggaaaccttatctatagagataagattgtatatgcgtaggagagcctggtcacgtaggcactttgcgcacggcactagggctgtggaggggacaggctatataaagcccgtttgcccaactcgtaaatcagtatcaattgtgctccggcgcacacgctcgcttgcgcgccggatagtataagtaattgataacgggcaacgcaacatgataagaaccagcagtcacgtgctgaacgtccaggaaaatataatgacgtcaaactgtgcgtcatcgccatattcgtgcgaggcaacgtccgcttgcgcagaagctcagcaggtaatgatcgataactttgttttctttcacatgtacaacgccgacatacaaattgacgcaaagctgcaatgcggcgtgcgctcggccgcgtttgcaatgatcgacgataaacatttggaaatgtacaagcatagaatagagaataaatttttttattactatgatcaatgtgccgacattgccaaacccgaccgtctgcccgatgacgacggcgcgtgctgtcaccattttatttttgatgcccaacgtattattcaatgtattaaagagattgaaagcgcgtacggcgtgcgtgatcgcggcaatgtaatagtgttttatccgtacttgaaacagttgcgagacgcgttgaagctaattaaaaactcttttgcgtgttgttttaaaattataaattctatgcaaatgtacgtgaacgagttaatatcaaattgcctgttgtttattgaaaagctggaaactattaataaaactgttaaagttatgaatttgtttgtagacaatttggttttgtacgaatgcaatgtttgtaaagaaatatctacggatgaaagatttttaaagccaaaagaatgttgcgaatacgctatatgcaacgcgtgctgcgttaacatgtggaagacggccaccacgcacgcaaaatgtccagcgtgcaggacatcgtataaataagcacgcaacgcaaaatgagtggtggcggcaacttgttgactctggaaagagatcattttaaatatttatttttgaccagctattttgatttaaaagataatgaacatgttccttcagagcctatggcatttattcgcaattacttgaattgcacgtttgatttgctagacgatgccgtgctcatgaactatttcaattacttgcaaagcatgcaattgaaacatttggtgggcagcacgtcgacaaacattttcaagtttgtaaagccacaatttagatttgtgtgcgatcgcacaactgtggacattttagaatttgacacgcgcatgtacataaaacccggcacgcccgtgtacgccacgaacctgttcacgtccaatccccgcaagatgatggctttcctgtacgctgaatttggcaaggtgtttaaaaataaaatattcgtaaacatcaacaactacggctgcgtgttggcgggcagtgccggtttcttgttcgacgatgcgtacgtggattggaatggtgtgcgaatgtgtgcggcgccgcgattagataacaacatgcatccgttccgactgtatctactgggcgaggacatggctaagcactttgtcgataataatatactaccgccgcacccttctaacgcaaagactcgcaaaatcaacaattcaatgtttatgctgaaaaacttttacaaaggtctgccgctgttcaaatcaaagtacacggtggtgaacagcactaaaatcgtgacccgaaaacccaacgatatatttaatgagatagataaagaattaaatggcaactgtccgtttatcaagtttattcagcgcgactacatattcgacgcccagtttccgccagatttgcttgatttgctaaacgaatacatgaccaaaagctcgatcatgaaaataattaccaagtttgtgattgaagaaaaccccgctatgagcggtgaaatgtctcgcgagattattcttgatcgctactcagtagacaattatcgcaagctgtacataaaaatggaaataaccaaccagtttcctgtcatgtacgatcatgaatcgtcgtacatttttgtgagcaaagactttttgcaattgaaaggcactatgaacgcgttctacgcgcccaagcagcgtatattaagtattttggcggtgaatcgtttgtttggcgccacggaaacgatcgactttcatcccaacctgctcgtgtaccggcagagttcgccgccggtccgtttgacgggcgacgtgtatgttgttgataagaacgaaaaagtttttttggtcaaacacgtgttctcaaacacggtgcctgcatatcttttaataagaggtgattacgaaagttcgtctgagttgaaatcccttcgcgatttgaatccgtgggttcagaacacgcttctcaaattattaatccccgactcggtacaataatatgatttacactgatcccactactggcgctacgactagcacagacgncgnccgtccacaaactatttaaacaggctaactccaaacatgttcttgaccatcttggctgtagtagtaattattgctttaataattatatttgttcaatctagcagtaatggaaacagctcggggggtaatgtacctccaaacgccctggggggttttgtaaatcctttaaacgctaccatgcgagctaatccctttatgaacacgcctcaaaggcaaatgttgtagataagtgtataaaaaatgaaacgtatcaaatgcaacaaagttcgaacggtcaccgagattgtaaacagcgatgaaaaaatccaaaagacctacgaattggctgaatttgatttaaaaaatctaagcagtttagaaagctatgaaactctaaaaattaaattggcgctcagcaaatacatggctatgctcagcaccctggaaatgactcaaccgctgttggaaatatttagaaacaaagcagacactcggcagattgccgccgtggtgtttagcacattagcttttatacacaatagattccatccccttgttactaattttactaacaaaatggagtttgtggtcactgaaaccaacgacacaagcattcccggagaacccattttgtttacggaaaacgaaggtgtgctgctgtgttccgtggacagaccgtctatcgttaaaatgctaagccgcgagtttgacaccgaggctttagtaaactttgaaaacgacaactgcaacgtgcggatagccaagacgtttggcgcctctaagcgcaaaaacacgacgcgcagcgatgattacgagtcaaataaacaacccaattacgatatggatttgagcgattttagcataactgaggttgaagccactcaatatttaactctgttgctgaccgtcgaacatgcctatttacattattatatttttaaaaattacggggtgtttgaatattgcaaatcgctaacggaccattcgctttttaccaacaaattgcgatcgacaatgagcacaaaaacgtctaatttactgttaagcaaattcaaatttaccattgaagattttgacaaaataaactcaaattctgtaacatcagggtttaatatatataattttaataaataattaaataatatacaatgtttttattaattatatttttaatattaattaaaagtattaatatttaaaaaaatgaatcaaattcatctaaagtgtcacagcgataaaatttgtcctaaagggtattttggcctcaacgccgatccctatgattgcacggcgtattatctgtgtccgcataaagtgcaaatgttttgcgaattaaatcacgaatttgacttggactccgccagctgcaagcctatcgtgtacgatcacacgggcagcgggtgtacggctcgcatgtatagaaacttgttactatgaagagcgggtttccagttgcacaacactattatcgatttgcagttcgggacataaatgtttaaatatatcgatgtctttgtgatgcgcgcgacatttttgtaggttattgataaaatgaacggatacgttgcccgacattatcattaaatccttggcgtagaatttgtcgggtccattgtccgtgtgcgctagcatgcccgtaacggacctcgtacttttggcttcaaaggttttgcgcacagacaaaatgtgccacacttgcagctctgcatgtgtgcgcgttaccacaaatcccaacggcgcagtgtacttgttgtatgcaaataaatctcgataaaggcgcggcgcgcgaatgcagctgatcacgtacgctcctcgtgttccgttcaaggacggtgttatcgacctcagattaatgtttatcggccgactgttttcgtatccgctcaccaaacgcgtttttgcattaacattgtatgtcggcggatgttctatatctaatttgaataaataaacgataaccgcgttggttttagagggcataataaaagaaatattgttatcgtgttcgccattagggcagtataaattgacgttcatgttggatattgtttcagttgcaagttgacactggcggcgacaagatcgtgaacaaccaagtgactatgacgcaaattaattttaacgcgtcgtacaccagcgcttcgacgccgtcccgagcgtcgttcgacaacagctattcagagttttgtgataaacaacccaacgactatttaagttattataaccatcccaccccggatggagccgacacggtgatatctgacagcgagactgcggcagcttcaaactttttggcaagcgtcaactcgttaactgataatgatttagtggaatgtttgctcaagaccactgataatctcgaagaagcagttagttctgcttattattcggaatcccttgagcagcctgttgtggagcaaccatcgcccagttctgcttatcatgcggaatcttttgagcattctgctggtgtgaaccaaccatcggcaactggaactaaacggaagctggacgaatacttggacaattcacaaggtgtggtgggccagtttaacaaaattaaattgaggcctaaatacaagaaaagcacaattcaaagctgtgcaacccttgaacagacaattaatcacaacacgaacatttgcacggtcgcttcaactcaagaaattacgcattattttactaatgattttgcgccgtatttaatgcgtttcgacgacaacgactacaattccaacaggttctccgaccatatgtccgaaactggttattacatgtttgtggttaaaaaaagtgaagtgaagccgtttgaaattatatttgccaagtacgtgagcaatgtggtttacgaatatacaaacaattattacatggtagataatcgcgtgtttgtggtaacttttgataaaattaggtttatgatttcgtacaatttggttaaagaaaccggcatagaaattcctcattctcaagatgtgtgcaacggcgagacggctgcacaaaattgtaaaaaatgccatttcgtcgatgtgcaccacacgtttaaagctgctctgacttcatattttaatttagatatgtattacgcgcaaaccacatttgtgactttgttacaatcgttgggcgaaagaaaatgtgggtttcttttgagcaagttgtacgaaatgtatcaagataaaaatttatttactttgcctattatgcttagtcgtaaagagagtaatgaaattgagactgcatctaataatttctttgtatcgccgtatgtgagtcaaatattaaagtattcggaaagtgtgcagtttcccgacaatcccccaaacaaatatgtggtggacaatttaaatttaattgttaacaaaaaaagtacgctcacgtacaaatacagcagcgtcgctaatcttttgtttaataattataaatatcatgacaatattgcgagtaataataacgcagaaaatttaaaaaaggttaagaaggaggacggcagcatgcacattgtcgaacagtatttgactcagaatgtagataatgtaaagggtcacaattttatagtattgtctttcaaaaacgaggagcgattgactatagctaagaaaaacaaagagttttattggatttctggcgaaattaaagatgtagacgttagtcaagtaattcaaaaatataatagatttaagcatcacatgtttgtaatcggtaaagtgaaccgaagagagagcactacattgcacaataatttgttaaaattgttagctttaatattacagggtctggttccgttgtccgacgctataacgtttgcggaacaaaaactaaattgtaaatataaaaaattcgaatttaattaattatacatatattttgaatttaattaattatacatatattttatattatttttgtcttttattatcgaggggccgttgttggtgtggggttttgcatagaaataacaatgggagttggcgacgttgctgcgccaacaccacctcctcctcctcctttcatcatgtatctgtagataaaataaaatattaaacctaaaaacaagaccgcgcctatcaacaaaatgataggcattaacttgccgctgacgctgtcactaacgttggacgatttgccgactaaaccttcatcgcccagtaaccaatctagacccaagtcgccaactaaatcaccaaacgagtaaggttcgatgcacatgagtgtttggcccgcaggaagatcgctaatatctacgtattgaggcgaatctgggtcggcggacggatcgctgccgcgacaaactgttttttctacttcatagttgaatccttggcacatgttggttagttcgggcggattgttaggcaacaaggggtcgaatgggcaaatggtaacatccgactgatttagattggggtcttgacgacaagtgcgctgcaataacaagcaggcctcggcgatttctccggcgtctttaccttgcacataataacttccgccggtgttattgatggcgttgattatatcttgtactagtgtggcggcgctaaacaagaaatagccgccggtggccaagagtatgcccgttcctcctacttttaagctttgcatgtaactatgtagacgggggttttgctgcagtgcgttttgaacaccttcgggcgtgcgcacgttggtttccgggaagttttgtttgactgcattggatcgcgtctgcttggtgtggtaattaaagtctggcacgttgtccacgcgccgcaattggctcaatgagtttatttgagggtctgaaatgccctgaaatactccgcgtatgttggggacatcattgttacgagtaattctgtttatgtctgaagtgctcacaaactggttgttagatagttgatagcccggctgaaatctgttgtttccaatgttgcgtacactgggcgcgttgagcacatttgtgaaaccggcgggagtgcttgttaaaagacgcgtattatcagtaataaaactggcctgattaggatacaatttattgactgcgcgaagatttgaaaaaaaactcattttaaagcaaacttatttaataaatatatcacagtaaaggttttgcaaaactgccgtcgtcaatacaacacggcagcggcgtcatgttggtaaaatctaatcttctccttgctttagattctgggcgagaaggcgcatttgttgtgtaagttatttcgacgtctgcattatttgttgtgtaaggtatctcgacgtatgaagcaactttaacattgttataattttttttaaatattgatgcgctccacggcgcgcgttgatacggatgatatctctccattgtatgatcgctaaatttatataccgtttcaataaatatgttaaaacccaacatgttaattataatattcataatagtttgtttgttttcaataattatttttactgttttgaaatctaaaagaggtgacgatgacgaatcagacgacgggttcagttgctataacaaaccaattggagtaaattttccgcatcctactagatgtgacgctttctacatgtgtgtcggtttaaatcaaaaattagagttaatctgccctgaaggatttgaatttgatccagatgttaaaaattgtgttcctatatcagattatggatgtaccgctaaccaaaactaaaaataaaataaaatttatatagattaatgaaataaaatttatatagattaataaaataaaatttatttaatatattatactatttatattatttacaacacttaacgtctagacataacagtttgtaacttagaaactaaatcagagttactgcgctcaaactctgaaaatttggcttgagactcggccacctgcttacgcaattgttcttgcagattattcacagtcgattgcaactcttctgatttcttggtagattcttgcaagtcatagtttgccttttgtaaatctaattcggcgacagcatgcttgtgtttaagcataatgtagtcgctgtttaacatggtcattttatgttcaacttggctggtcttggctcgcagctcggacagttctttttgcaattgctccacatagttcaagtccgtggtgtgattgttgaccgtgttattttctaaaagctcgcgccaatgctgtttgatggaatcctggttacgagtgacgttaatgggcataaattctacatacccgtgcttattgtacacgcgacaatctgatgaagtagcgctgcaaaaacatttgtacacagaattgtccataattatcttgacataacacttgaaacacacagcatggttacaatgaatcgaagtcacaaacgaggaatttacgtttttagtgtctttaaaagtagtaaaacaaatattacacgaaacctctacttcttcttcgggttctgattgctgctgctgctgctgctgcggctgcggagactgcggcgaggcaaacaaatctggcgactgtggtattacgtaattcggcgaataagatggactataagtgggagaccttggggcaatctcattcatcagctgagcctcaagatctaaacctcgttgcagagccctctgcgcagctgtctccgacgcaatgttatcctggtactgctgggcagtgatgtcgggaaaccgttcacgatccacattttcactattaattagtatgacgtcatcctcttgacttaatagcggatcgtcattgctaatgttaacctgaccgtgcacgtaatacgtgacaccctgacgatggtaggtgcgcgtcaacggctcgttgacgttcccgataatctgcacgttttcttcgctgacacgctgctcctgacgccgctcctgacggcgatggctgcgactgcttgaagacggctggctgcgactgcttgaagacggctgggcttcgggagatgttgtaaagttgatgcggcgacggctgagagacagcctgtggcggcggctgctgctgggagtggcggcgttgatttggcgactcatggctgggctggtaggatactgttcactaggctgtgaggcttgaactgtgcttacgagtagaacggcagctgtatttatactgtttatcagtactgcacgactgataagacaatagtggtgggggaacttgccaggcaaaaatgaacttttttgtaatgcaaaaaagttgatagtgtagtagtatattgggagcgtatcgtacagtgtagactattctaataaaatagtctacgatttgtagagattgtactgtatatggagtgtcaggcaaaagtgaacttttttgcattgcaaaaaaattcattttaaatttatcatatcacaggctgcagtttctgttatctgtcccccactcaggcgtgcagctataaaagcaggcactcaccaactcgtaagcacagttcgttgtgaagtgaacacggagagcctgccaataagcaaaatgccaagggacaccaacaatcgccaccggtctacgccatatgaacgtcctacgcttgaagatctccgcagacagttgcaagacaatttggacagcataaaccgccgagacagaatgcaagaagaacaagaagaaaacctgcgctatcaagtgcgtagaaggcagcgtcaaaaccagctccgctccatacaaatggaacagcagcgaatgatggcggaattaaacaacgagccggtgattaattttaaatttgagtgtagtgtgtgtttagaaacatattcccaacaatctaacgatacttgtccttttttgattccgactacgtgcgaccacggtttttgtttcaaatgcgtcatcaatctgcaaagcaacgcgatgaatattccgcattccactgtgtgctgtccattgtgcaatacccaggtaaaaatgtggcgttccttaaagcctaacgctgttgtgacgtgtaagttttacaagaaaactcaagaaagagttccgcccgtgcagcagtataaaaacattattaaagtgctacaagaacggagcgtgattagtgtcgaagacaacgacaataattgtgacataaatatggagaatcaggcaaagatagctgctttggaagctgaattggaagaagaaaaaaatcacagtgatcaagtagcttctgaaaaccgacagctgatagaagaaaatactcgtctcaatgaacagattcaagagttgcagcatcaggtgaggacattggtgccgcaacgtggcattacggttaatcagcaaattggccgtgacgacagtgcgccagccgagctgaacgagcgttttcgctcacttgtctattcgactatttcagagctgtttattgaaaatcgcgttcatagtattcaaaattatgtttatgccggaacttctgctgctagttcatgtgatgtaaatgttactgttaattttgggtttgaaaattaatgtgatatgaaatgtatatataaaaatgatggaataaataataaacatttttatactttttatgttttttttatttcatgtgattaagaaacttttaagatggatagtagtaattgtattaaaatagatgtaaaatacgatatgccgttacattatcaatgtgacaataacgcagataaagacgttgtaaatgcgtatgacactatcgatgttgaccccaacaaaagatttataattaatcataatcacgaacaacaacaagtcaatgaaacaaataaacaagttgtcgataaaacattcataaatgacacagcaacatacaattcttgcataataaaaatttaaatgacatcatatttgagaataacaaatgacattatccctcgattgtgttttacaagta
